# Prevalence and trends of multimorbidity clusters in Belgian assisted dying practice: a health registry study

**DOI:** 10.1093/ageing/afag153

**Published:** 2026-05-27

**Authors:** Jacques Wels, Natasia Hamarat, Juan González-Hijón, Luc Proot

**Affiliations:** University College London - Research Department of Population Science and Experimental Medicine, Unit for Lifelong Health and Ageing, Torrington Place 1-19, London WC1E 7HB, England, UK; Université libre de Bruxelles - Sociology Institute, Health & Society Research Unit, Av. Jeanne 44 CP124, Brussels 1050, Belgium; Université libre de Bruxelles - Sociology Institute, Health & Society Research Unit, Av. Jeanne 44 CP124, Brussels 1050, Belgium; Université libre de Bruxelles - Sociology Institute, Health & Society Research Unit, Av. Jeanne 44 CP124, Brussels 1050, Belgium; Federal Commission for Control and Evaluation of Euthanasia, Brussels, Belgium

**Keywords:** assisted dying, cumulative illness rating scale for geriatrics, severity, multimorbidity, Belgium, older people

## Abstract

**Background:**

Population ageing is accompanied by a rise in complex multimorbidity, i.e. the co-occurrence of two or more chronic or acute conditions. For a growing number of people, this cumulative disease burden results in unbearable suffering. Using 22 years of national data from Belgium, we investigated the prevalence and clinical profiles of such suffering at the end of life, as reflected in requests for voluntary assisted dying (VAD).

**Methods:**

We analysed all anonymised VAD cases reported to the Federal Commission for Control and Evaluation of Euthanasia between 2003 and 2024 (*N* = 6153). Conditions severity was assessed using the Cumulative Illness Rating Scale for Geriatrics (CIRS-G, 1–4 scale). Sex-specific multimorbidity clusters were identified and temporal trends were modelled with negative binomial regression incorporating population-adjusted offsets, testing interactions with sex, cluster, age group and language region and with and without spline to address nonlinear trends.

**Results:**

Patients had a mean age of 83.1 years, with 58.2% female, an average of 2.79 comorbid conditions and a mean CIRS-G of 3.43. Five sex-specific clusters were identified, with females exhibiting musculoskeletal, psychiatric and cardiovascular burdens, and males demonstrating cardio-respiratory and neurological profiles. Overall incidence increased over time, particularly among the oldest age groups. Cluster-specific trends revealed rapid growth in female musculoskeletal-psychiatric profiles and dynamic temporal changes in male cardiac-dominant clusters. Language region and age modified these trends, with Dutch-speaking males showing initially high but declining incidence in cardiac-dominant clusters.

**Conclusions:**

VAD for multimorbidity is heterogeneous, evolving and strongly influenced by ageing and cluster-specific disease patterns.

## Key Points

- Multimorbidity is the second-leading cause of assisted dying where legislated; this study provides its first long-term epidemiological profile.
- The rising incidence is concentrated in the oldest old (80+) and driven by distinct, sex-specific disease clusters.
- Women show sharp growth in a musculoskeletal-psychiatric cluster, while men exhibit increases in cardio-respiratory and neurological clusters.
- Regional disparities in cluster prevalence suggest medical culture and access influence end-of-life pathways for complex multimorbidity.
- Findings enable targeted geriatric care models aligned with the specific suffering profiles preceding assisted dying requests.

## Background

As populations age and chronic disease accumulates [1], voluntary assisted dying (VAD) increasingly involves patients with multiple morbidities rather than a single diagnosis—yet the epidemiological dynamics of this shift remain unexamined.

Multimorbidity—i.e. the presence of two or more health conditions—is the second leading cause of VAD [2]. In Belgium, between 2002 and 2023, cancers accounted for 65% of VAD cases, followed by multimorbidity, which concerned a total of 15% of patients [3], 71% of whom involved 80+ patients [4]. While cancers remain the leading cause of VAD, multimorbidity cases have increased more sharply in comparison [3]. The same trend is observed in other VAD legislations such as in Canada [5] and the Netherlands, [6]. Yet, multimorbidity remains a blind spot in epidemiological research on VAD.

In Belgium, euthanasia is the term used to qualify VAD in both the legislation and common language [7]. Under Belgian law, eligible patients must experience ‘constant and unbearable physical or mental suffering that cannot be alleviated’ resulting from a serious and incurable medical condition. The law imposes strict criteria [8] and the legal definition implies and permits euthanasia in cases of multimorbidity.

As multimorbidity arises from the interactions among diseases rather than their simple sum [9, 10], cluster analyses are often needed to identify clinically meaningful patterns of co-occurring diseases [11–13]. These clusters exert distinct effects on mortality, functional decline and quality of life [14–16], with prognostic relevance evident across conditions [17]. Visualisation techniques further help to interpret this complexity [18]. To quantify burden within clusters, severity scoring systems like the Cumulative Illness Rating Scale for Geriatrics (CIRS-G) provide standardised, nuanced assessments beyond mortality-based indices [19, 20]. Mapping disease clusters with graded severity offers a robust, evidence-based profile of total illness burden—crucial in the context of VAD, where suffering refractory to treatment is a core criterion [9, 21].

Understanding trends in cluster prevalence requires to address demographic change—such as population ageing, changing sex ratios and regional or language-related differences—rather than by shifts in disease patterns alone [22, 23].

The research questions of this study are three-fold. [RQ.1] What are the overall trends of multimorbidity over the study period, and how do these trends differ by sex, language, age, number and types of conditions, and severity? [RQ.2] What clusters of conditions can be identified among patients, and how do these clusters vary by sex? [RQ.3] What are the observed VAD for multimorbidity prevalence trends when accounting for demographic composition and change and how do trends vary by sex, region and age-group?

## Data and methods

### Data

We used data routinely collected between September 2002 (when the law entered into force) and December 2024 by the Federal Commission for the Control and Evaluation of Euthanasia (FCCEE), derived from individual reports submitted by euthanasia practitioners. These reports are fully anonymised and encompass all reported euthanasia cases since 2002, including information on the reasons for euthanasia, as well as the patients’ demographics. No descriptive statistics are shown for year 2002 because of low count.

This study focuses exclusively on patients classified as receiving euthanasia on the basis of multimorbidity. This classification is made by the FCCEE based on individual reports submitted by medical practitioners.

Population yearly counts as of the 1st January for each selected year (2003 to 2024) by age group, sex and region of residence were retrieved from Federal Agency for National Statistics (*Statbel*). Belgium’s federal structure includes three regions (Wallonia, Flanders and Brussels). While the patient’s place of residence was not consistently collected by the FCCEE until recently, the language used by the reporting medical practitioner was systematically included. This allows us to impute regional differences by distinguishing euthanasia cases reported in Dutch or French. The French-speaking population was calculated as the sum of the population residing in Wallonia and 90% of the Brussels population and the Dutch speaking as the sum of the Flanders residents and 10% of the Brussels population, reflecting the Belgian language repartition. We used population figures instead of the number of deaths by subgroup as done in previous studies [24–28] because a non-negligible share of euthanasia is performed on patients not expected to die in the foreseeable future [29].

The Belgian FCCEE granted ethical approval and data access on 14 May 2024 after we submitted a detailed research proposal to the FCCEE. This was done in full compliance with the 2002 Belgian law governing euthanasia and data protection. Consent to participate was waived by the FCCEE in accordance with Belgian regulations. This study received a positive opinion from the Université libre de Bruxelles (ULB) Data Protection Officer under the General Data Protection Regulation (RGPD/GDPR) (Avis n° R2025/023, 14 November 2025).

### Variables

#### Conditions and CIRS-G

In Belgium, the FCCEE defines multimorbidity (called ‘polypathology,’ a term often used as a substitute to multimorbidity in the French or German literature [30]) as ‘the co-occurrence of multiple chronic or acute diseases or medical conditions within one person’ [10, 31]. For the present study, conditions were categorised into the following systems: musculoskeletal, neurological, infections, COVID-19, haematological, respiratory, cardiac, vascular, endocrine/systemic diseases, dermatological, trauma, genitourinary, gastrointestinal, eye/ear/balance, psychiatric, dementia and memory disorders, symptoms and complaints, congenital disorders and neoplasms. Somatic suffering by conditions was evaluated using the CIRS-G [19, 32]. The severity of each condition is scored on a five-point scale from 0 to 4 [33], where 4 represents an extremely severe illness [34]. The Severity Index was derived by averaging the scores only from the CIRS-G organ system categories [33, 35] in which a pathology was identified and scored.

#### Patients’ characteristics

FCCEE include data on patient profiles including sex (female or male), language (French or Dutch) and age group.

### Methods

#### Descriptive statistics

We first generated yearly descriptive statistics on the total number of cases, average number of conditions per patient, mean severity index, average age, French-speaking to Dutch-speaking ratio, female to male ratio, as well conditions occurrence and average severity score by condition not adjusting for demographic offsets.

#### Morbid conditions clustering

We performed clustering analyses to identify disease patterns separately for male and female patients. For each sex, a subset of the dataset containing severity indicators of 19 disease categories was extracted. To determine the optimal number of clusters (k), we assessed solutions from 2 to 10 using the Hopkins statistic, average silhouette width, elbow method, gap statistic and bootstrap stability. Given the modest silhouette widths but high stability and clinical interpretability, we selected a five-cluster solution. To compute pairwise dissimilarities between patients, we applied the Gower distance, which accommodates mixed data types. Partitioning Around Medoids (PAM) clustering was then performed.

#### Negative binomial regression with demographic offset

We performed a sequence of negative binomial regression models. The offset for all models was the natural logarithm of the total population, stratified by age, sex and region. A descriptive baseline model (Model 0) examined the linear and nonlinear temporal trends for each of the 19 individual morbidities, interacted with sex. Subsequent models analysed the identified morbidity clusters. Model 1 assessed the overall temporal trend in the incidence of euthanasia for multimorbidity, separately for each sex. Model 2 introduced an interaction between time and the morbidity clusters to assess cluster-specific trends. Model 3 incorporated a three-way interaction between time, patient cluster and language region. Finally, Model 4 examined a three-way interaction between the temporal spline, patient cluster and age group; for this model, the dataset was restricted to the age groups 60–69, 70–79 and 80–89, where most cases were present. For each model, we assessed both linear and nonlinear temporal trends, the latter using a natural spline with four degrees of freedom. Results are presented as incidence rate ratios (IRRs). This IRR represents the yearly rate of change in incidence for patients with that condition: a value of 1 indicates no change over time, a value below 1 indicates a decreasing trend and a value above 1 indicates an increasing trend. Predictions from the spline models are expressed as incidence rates per 1000 to facilitate interpretation.

#### Software

All analyses were conducted using the R statistical software. The package ‘hopkins’ was used to determine the number of clusters [36]. The package ‘cluster’ was used for clustering [37]. Negative binomial regressions were made using the package ‘MASS’ [38].

## Results

### RQ.1. Descriptive statistics

As shown in Appendix 1 in the Supplementary Data section, the proportion of multimorbidity cases in Belgian euthanasia practice increased from 8.7% in 2005 to 17.3% in 2020, while cancer cases correspondingly declined from 80.4% to 64.1%.

**Figure 1:**
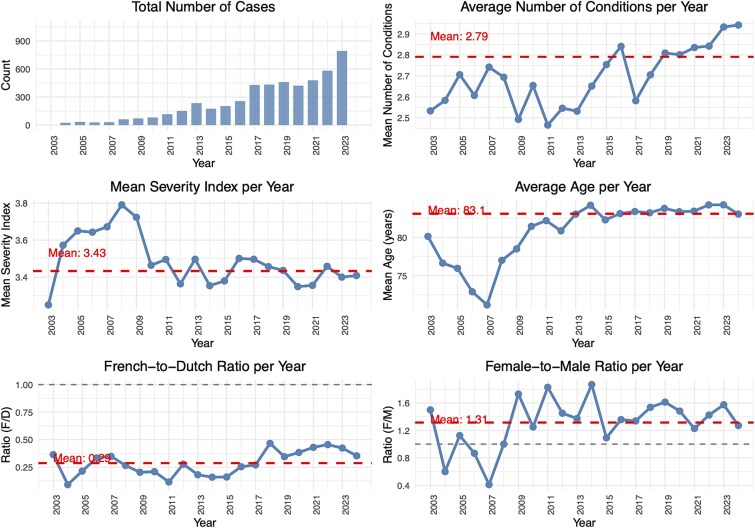
Descriptive statistics on the total number of cases per year, average number of conditions per year, mean severity index per year, average age per year, French speaking-to-Dutch speaking ratio per year and female-to-male ratio per year. Note: the French-to-Dutch and female-to-male ratios represent the ratio between the number of French-speaking or female patients relative to the number of Dutch-speaking or male patients per year.

Descriptive statistics on the total number of multimorbidity cases, comorbid conditions means, severity index, sex and language ratio, mean age are shown in Figure 1 (estimates are in Appendix 2 in the Supplementary Data section). This study analysed a total of 6154 cases spanning from 2002 to 2024. The annual number of cases demonstrated a substantial increase over the study period, rising from 15 cases in 2003 to 1070 cases in 2024. The distribution of cases across years shows a particularly notable >2017 acceleration, with the highest number of cases recorded in the most recent.

The study population had a mean age of 83.1 years (SD = 11.11) across the entire study period, with ages ranging from 22 to 105. The mean age remained relatively stable throughout the years, fluctuating between 71 in 2007 and 84 in both 2022 and 2023.

Patients had a mean of 2.79 comorbid conditions (SD = 0.93). The number of conditions showed a slight increasing trend over time, from 2.53 in 2003 to 2.94 in 2024.

The severity index showed consistent values across the study period, with an overall mean of 3.43 (SD = 0.41). Yearly means remained stable, ranging from 3.25 (2003) to 3.79 (2008). Missing severity data were minimal (52 cases, 0.85%).

Females outnumbered males throughout most of the study period, with an overall female-to-male ratio of 1.39. The total sample included 3582 females (58.2%) and 2570 males (41.8%). The female-to-male ratio varied across years, ranging from 0.41 in 2007 to 1.87 in 2014.

Dutch-speaking cases predominated (74.7%, *n* = 4596) over French-speaking cases (25.3%, *n* = 1556), with an overall French-to-Dutch ratio of 0.34. This ratio increased over time from 0.36 in 2003 to 0.47 in 2018.

Table 1 exhibits the prevalence and mean severity scores for the 19 disease categories in all years and by sex while the detail by year is shown in Appendix 4 in the Supplementary Data section. Musculoskeletal disorders were the most common (44.1%), particularly among females (54.9% vs. 29.0%). Females also had higher prevalence of eye/ear/balance disorders (31.6% vs. 19.9%) and psychiatric conditions (13.3% vs. 8.6%). Males more frequently presented with cardiac disease (44.7% vs. 37.9%), respiratory conditions (28.6% vs. 18.0%), vascular disease (10.1% vs. 7.2%) and neoplasms (38.3% vs. 23.9%). Average CIRS-G were similar between sexes, generally ranging from 3.0 to 3.8 across most conditions. The highest severity was observed for neoplasms (3.7–3.8).

**Table 1:** Conditions occurrence and average severity score, by sex.

	Overall (*N* = 6,153)			Female (*N* = 3,583)			Male (*N* = 2,570)		
Condition	Occurrence	(*N*)	Average severity score	Occurrence	(*N*)	Average severity score	Occurrence	(*N*)	Average severity score
Musculoskeletal	44.06	(2711)	3.40	54.86	(1966)	3.41	28.99	(745)	3.35
Cardiac	40.74	(2507)	3.51	37.91	(1359)	3.50	44.67	(1148)	3.52
Neoplasms	29.90	(1840)	3.74	23.90	(856)	3.67	38.29	(984)	3.81
Neurological	28.44	(1750)	3.43	26.91	(964)	3.39	30.58	(786)	3.47
Eye ear balance	26.70	(1643)	3.22	31.57	(1131)	3.20	19.92	(512)	3.27
Respiratory	22.44	(1381)	3.53	18.03	(647)	3.50	28.56	(734)	3.55
Genitourinary	14.90	(917)	3.29	13.90	(498)	3.24	16.30	(419)	3.35
Psychiatric	11.30	(695)	3.29	13.26	(475)	3.29	8.56	(220)	3.28
Gastrointestinal	10.17	(626)	3.27	10.69	(383)	3.22	9.46	(243)	3.35
Trauma	9.20	(565)	3.32	8.88	(318)	3.27	9.65	(248)	3.38
Endocrine systemic diseases	9.18	(565)	2.67	8.51	(305)	2.60	10.12	(260)	2.74
Vascular	8.39	(516)	3.43	7.15	(256)	3.39	10.12	(260)	3.48
Symptoms and complaints	7.85	(483)	3.05	9.24	(331)	3.07	5.91	(152)	3.00
Dementia, memory disorders	4.96	(305)	2.93	5.19	(186)	2.90	4.63	(119)	2.97
Dermatological	4.47	(275)	3.14	4.89	(175)	3.14	3.89	(100)	3.13
Infections	3.36	(207)	3.34	2.74	(98)	3.37	4.24	(109)	3.32
Haematological	2.13	(131)	3.16	2.09	(75)	3.17	2.18	(56)	3.14
COVID-19	0.75	(46)	1.74	0.75	(27)	1.74	0.74	(19)	1.74
Congenital	<0.20	(<10)	3.57	<0.20	(<10)	3.67	<0.20	(<10)	3.00

### RQ.2. Comorbid conditions clustering

We generate five clusters by sex (see Appendix 3 in the Supplementary Data section). The 1–5 female clusters represent a total of respectively 1161, 1967, 1140, 1704 and 1384 patients. The 1–5 male clusters represent a total of respectively 1125, 725, 1040, 714 and 878 patients.

**Figure 2:**
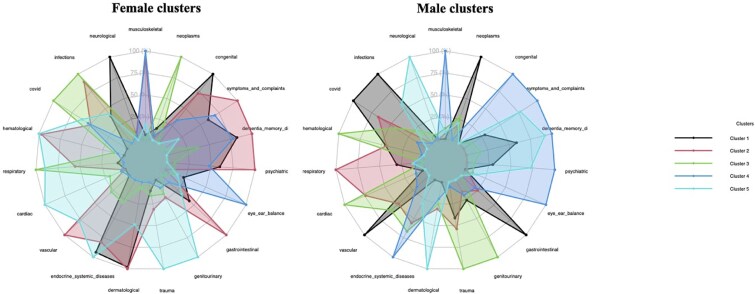
Radar plots of multimorbidity clusters by gender (female on the left and male on the right). Legend: the radar plots display the prevalence (in percent) of 19 chronic conditions across the identified multimorbidity clusters. Each axis represents a specific condition, scaled from 0% to 100%. The plotted area illustrates the condition profile of each cluster, with greater radial distance indicating higher prevalence within that cluster.

The radar plots in Figure 2 illustrate the mean values of each cluster across the included variables, shown separately for men and women. Each axis corresponds to a variable, and values are rescaled (0%–100%); the percentage labels on the axes represent the relative position of a cluster mean within the observed range of that variable. The shape of each cluster profile indicates how it differs across variables and in relation to other clusters.

In females, Cluster 1 was dominated by neurological disease; Cluster 2 by musculoskeletal conditions; Cluster 3 showed moderate elevations across respiratory, cardiac and musculoskeletal categories; Cluster 4 combined musculoskeletal and psychiatric burden; and Cluster 5 was characterised by high cardiac involvement. In males, Cluster 1 showed moderate respiratory and neoplasm involvement with additional musculoskeletal and neurological conditions. Cluster 2 was dominated by high respiratory and cardiac scores, while Cluster 3 was primarily characterised by cardiac disease. Cluster 4 exhibited elevated musculoskeletal and eye/ear/balance scores. Cluster 5 showed very high neurological scores alongside moderate psychiatric and musculoskeletal involvement.

### RQ.3. Demographic adjusted time trends in multimorbidity clusters

Table 2 presents the IRRs for each condition by year and sex (Appendices 4 and 5). We observe that, for females, the strongest annual decreases were observed for dementia and memory disorders (IRR 0.964) and cardiac conditions (IRR 0.981). A similar pattern was seen in males for dementia and memory disorders (IRR 0.936) and, notably, for psychiatric conditions (IRR 0.939). It is crucial to note that no condition in either sex demonstrated a significant increasing trend over the study period; the IRRs for all other conditions, such as neoplasms and respiratory diseases, clustered closely around 1.00, indicating generally stable incidence over time. Only strong variations are observed for COVID-19 but this is due to the first cases being observed in 2020.

**Table 2:** Negative binomial regression of each conditions, incidence rate ratio per year and 95%CI.

	Female			Male		
	IRR	95% CI		IRR	95% CI	
		Lower	Upper		CI lower	CI upper
Cardiac	0.981	0.964	0.999	0.999	0.978	1.020
Congenital	4.071	0.641	28.789	1.000	1.000	1.000
COVID-19	1.154	0.753	1.752	0.773	0.447	1.300
Dementia, memory disorders	0.964	0.931	0.998	0.936	0.893	0.981
Dermatological	0.964	0.934	0.994	1.037	0.991	1.085
Endocrine systemic diseases	0.989	0.964	1.014	0.993	0.965	1.023
Eye ear balance	0.994	0.974	1.013	1.004	0.974	1.034
Gastrointestinal	1.000	0.975	1.025	0.971	0.941	1.002
Genitourinary	1.005	0.980	1.030	1.014	0.988	1.041
Haematological	0.968	0.905	1.036	1.042	0.978	1.110
Infections	0.971	0.922	1.024	1.016	0.976	1.057
Neoplasms	1.006	0.987	1.026	1.008	0.986	1.031
Neurological	0.995	0.975	1.014	0.994	0.970	1.017
Musculoskeletal	1.006	0.989	1.023	0.997	0.971	1.024
Psychiatric	0.987	0.964	1.012	0.939	0.904	0.976
Respiratory	0.983	0.963	1.004	1.007	0.984	1.030
Symptoms and complaints	0.998	0.970	1.026	0.969	0.921	1.017
Trauma	1.016	0.978	1.057	0.997	0.950	1.046
Vascular	0.976	0.948	1.005	0.981	0.948	1.015

These condition-specific trends, however, provide an incomplete picture. The clinical reality at the end of life is predominantly one of multimorbidity, where the interplay of conditions likely dictates trajectories more than any single diagnosis.

Using the clustering approach, we first addressed overall temporal trends in all cases of euthanasia for multimorbidity by sex adjusting for demographics using linear and spline models.

**Figure 3:**
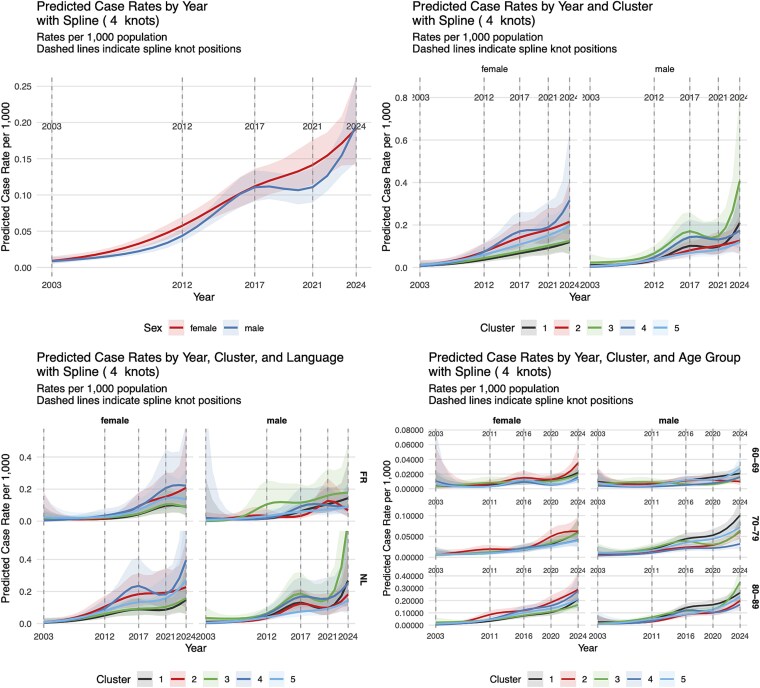
Predicted yearly prevalence (per 1000) of euthanasia for multimorbidity by sex, cluster, language and age group from 2003 to 2024.

Unlike what was observed in the descriptive section, the same increase is observed in both male and female patients. The linear model shows coefficients of respectively 0.140 (95% CI: 0.120, 0.160) and 0.132 (95% CI: 0.112, 0.152). Predicted prevalences in the model using splines are shown in Figure 3 (upper left panel) with full estimates in Appendices 6 and 7 for the linear and splines models, respectively. VAD for multimorbidity concerned between 0.15 and 0.20 individuals per 1000 in 2024, corresponding to at most 2 cases per 10,000 people per year. Prevalences are comparable across sex.

When looking a clusters trends by sex, linear estimates of the interaction between clusters and years show a non-significant increase in Clusters 4 in female of 0.029 (95% CI: −0.034, 0.092), relative to Cluster 1. This is shown in Figure 3 (upper right panel) where predicted yearly prevalences of Cluster 4 have sharply increased over the recent years in comparison with the other clusters.

In males, Figure 3 indicates increased predicted prevalences in Clusters 1 and 3. Predicted prevalence in the model with spline, however, show a prevalence of 0.211 (95% CI: 0.112, 0.398) and 0.408 (95% CI: 0.207, 0.808) in 2024 for Clusters 1 and 3. By comparison, prevalences were respectively 0.064 (95% CI: 0.043, 0.094) and 0.119 (95% CI: 0.079, 0.181) in 2014.

Results for Model 3 show different patterns by region. First, the linear model shows that, relatively to the French-speaking region, there was a reduction in cases in the Dutch-speaking region (Year x Language:NL: −0.018, 95% CI: −0.105, 0.069) but with higher total cases observed in the Dutch-speaking region.

Figure 3 (lower left panel) shows the predicted prevalence derived from the spline model.

In the French-speaking region, the most pronounced increases were observed in Clusters 3 and 4 for males, with predicted prevalences rising from 0.000 (95% CI: 0.000, 0.018) and 0.018 (95% CI: 0.000, 0.770) in 2003 to 0.186 (95% CI: 0.070, 0.493) and 0.083 (95% CI: 0.031, 0.224) in 2024. Among females, Clusters 2 and 4 showed the strongest growth, increasing from 0.019 (95% CI: 0.002, 0.215) and 0.004 (95% CI: 0.000, 0.075) to 0.207 (95% CI: 0.081, 0.534) and 0.223 (95% CI: 0.082, 0.606).

In the Dutch-speaking region, the most substantial increases were seen in Clusters 3 and 4 for males, with prevalences climbing from 0.035 (95% CI: 0.011, 0.107) and 0.003 (95% CI: 0.001, 0.012) in 2003, 0.623 (95% CI: 0.244, 1.594) and 0.249 (95% CI: 0.103, 0.601) in 2024. For females, Clusters 4 and 5 demonstrated the highest final estimates, reaching 0.382 (95% CI: 0.148, 0.984) and 0.265 (95% CI: 0.102, 0.687) in 2024, respectively, from much lower starting points.

Finally, results for Model 4 are shown in the lower right panel. The predicted prevalence demonstrated a positive association with age, with the most substantial increases observed in the oldest age group (80–89). For instance, in Cluster 1, the predicted prevalence for 80–89 year-olds rose sharply from 0.007 (95% CI: 0.002, 0.031) in 2003 to 0.212 (95% CI: 0.141, 0.317) in 2024. This age gradient was consistent across most clusters, with the 70–79 age group exhibiting a more moderate increase. In contrast, the 60–69 age group showed the most stable and lowest predicted prevalences throughout the study period.

## Discussion

The global rise in multimorbidity represents a major challenge for ageing societies [39], with implications extending to end-of-life decision-making [40, 41]. Multimorbidity is the second leading cause of VAD in jurisdictions where it is permitted, yet empirical evidence on its characteristics and evolution remains limited [32]. This study is, to our knowledge, the first to systematically describe long-term trends and clinical profiles of assisted dying for multimorbidity in Belgium or in any other country.

We found that the number of euthanasia cases for multimorbidity increased substantially between 2002 and 2024. However, this growth occurred without corresponding rises in the number or average severity of conditions per patient. Increases were concentrated in distinct clusters that differed by sex, age and region. In women, the sharpest growth occurred among those with co-occurring musculoskeletal and psychiatric conditions, or neurological and sensory impairments. In men, increases were dominated by cardio-respiratory and neurological clusters. These constellations likely represent divergent pathways to unbearable suffering: the former linked to chronic pain, loss of function and psychological distress; the latter to progressive organ failure and functional disintegration.

When demographic composition was accounted for, sex differences diminished, and the persistence of high rates among individuals aged 80–89 underscored that euthanasia for multimorbidity primarily concerns patients aged 80+. Here, suffering derives less from a single terminal diagnosis than from the cumulative, interactive burden of chronic conditions associated with dependency and declining autonomy. Regional contrasts further indicate that medical culture, palliative care infrastructure and societal attitudes may influence both thresholds of suffering or clinical responses.

Administrative data lack information on socioeconomic status, living arrangements and denied requests, restricting assessment of equity. Aggregating conditions into 19 categories reduces diagnostic granularity but enhances comparability, capturing the combined effects of coexisting illnesses generating refractory suffering rather than disease-specific pathways. Finally, direct harmonised measures of frailty, functional decline or loss of autonomy were unavailable in this dataset, although these factors often play a greater role in end-of-life suffering in geriatric populations than diagnostic burden alone. Our analysis, therefore, relies on chronic disease burden measured by CIRS-G severity scores, which correlate with but do not fully capture frailty [42]. Although analyses were stratified by age—a key correlate of frailty [43]—functional heterogeneity within age groups could not be assessed, potentially underestimating ageing-related vulnerability. Future studies should incorporate direct functional measures or validated claims-based frailty proxies when available.

These findings have implications for geriatric and end-of-life care. The prominence of musculoskeletal–psychiatric multimorbidity in female points to the need for integrative care models addressing pain, functional loss and mental health concurrently. Likewise, the high representation of cardio-respiratory and neurological clusters in male highlights the need for anticipatory management of advanced multimorbidity in later life. Finally, although the nature of the gap across Belgian region remains to be fully understood, differences in cluster prevalence across regions may raise questions about equity and consistency in the application of VAD legislation. Ensuring that comparable suffering leads to comparable care options should be a central policy goal.

## Supplementary Material

afag153_aa_25_3681_File002

## Research Data Transparency and Availability

Data access may be granted upon request to the FCCEE.
