# Supplementary material for: Prevalence and trends of multimorbidity clusters in Belgian assisted dying practice: a health registry study
Source: Age Ageing. 2026 May 27;55(5):afag153. doi: 10.1093/ageing/afag153 (PMC13213452; doi:10.1093/ageing/afag153)
Supplement: afag153_aa_25_3681_File002 [file afag153_aa_25_3681_file002.docx]

**Prevalence and trends of multimorbidity clusters in Belgian assisted dying practice: A health registry study**

**Supplementary data**

**Appendices**

[Appendix 1. Euthanasia cases by Reason and Year 2](#_Toc225179766)

[Appendix 2. Descriptive statistics (2003 to 2024) 3](#_Toc225179767)

[Appendix 3. Optimal number of clusters 5](#_Toc225179768)

[a. All cases 5](#_Toc225179769)

[b. Female cases 7](#_Toc225179770)

[c. Male cases 9](#_Toc225179771)

[d. Pairwise correlations across conditions and clustering visualisation, by sex 11](#_Toc225179772)

[Appendix 4. Negative binomial regression of each conditions (model 0) 15](#_Toc225179773)

[Appendix 5. Negative binomial regression of each conditions with splines (model 0) 17](#_Toc225179774)

[Appendix 6. Negative binomial regression of Clusters 33](#_Toc225179775)

[6.1. Model 1: Negative binomial regression of year, by sex 33](#_Toc225179776)

[6.2. Model 2: Negative binomial regression of year x cluster by sex 33](#_Toc225179777)

[6.3. Model 3: Negative binomial regression of year x cluster x language, by sex 34](#_Toc225179778)

[6.4. Model 4: Negative binomial regression of year x cluster x age groups, by sex 35](#_Toc225179779)

[Appendix 7. Negative binomial regression of Clusters with splines 39](#_Toc225179780)

[7.1. Model 1: Negative binomial regression of year, by sex 39](#_Toc225179781)

[Predictions 39](#_Toc225179782)

[Model estimates 41](#_Toc225179783)

[7.2. Model 2: Negative binomial regression of year x cluster by sex 43](#_Toc225179784)

[Predictions 43](#_Toc225179785)

[Model estimates 48](#_Toc225179786)

[7.3. Model 3: Negative binomial regression of year x cluster x language, by sex 51](#_Toc225179787)

[Predictions 51](#_Toc225179788)

[Model estimates 67](#_Toc225179789)

[7.4. Model 4: Negative binomial regression of year x cluster x age groups, by sex 73](#_Toc225179790)

[Predictions 73](#_Toc225179791)

[Model estimates 86](#_Toc225179792)

# Appendix 1. Euthanasia cases by Reason and Year

Note: Total data on all euthanasia cases were not fully available for the year 2024 at the time this study was written.

# Appendix 2. Descriptive statistics (2003 to 2024)

|  | **Total cases** | **Age** | | | **Sex** | | **Language** | | **Number Conditions** | | | **Severity Score** | | | | |
| --- | --- | --- | --- | --- | --- | --- | --- | --- | --- | --- | --- | --- | --- | --- | --- | --- |
| year |  | *Mean* | *SD* | *Missing* | *Female / Male*  *ratio* | *Missing* | *French / Dutch*  *ratio* | *Missing* | *Mean* | *SD* | *Missing* | *Mean* | *SD* | *Min* | *Max* | *Missing* |
| 2003 | 15 | 80.14 | 10.39 | 0 | 1.5 | 0 | 0.36 | 0 | 2.53 | 0.83 | 0 | 3.25 | 0.38 | 2.75 | 4 | 0 |
| 2004 | 24 | 76.65 | 12.13 | 0 | 0.6 | 0 | 0.09 | 0 | 2.58 | 0.72 | 0 | 3.57 | 0.29 | 3 | 4 | 0 |
| 2005 | 34 | 75.96 | 11.64 | 0 | 1.12 | 0 | 0.21 | 0 | 2.71 | 0.87 | 0 | 3.65 | 0.41 | 2.75 | 4 | 0 |
| 2006 | 28 | 72.89 | 13.89 | 0 | 0.87 | 0 | 0.33 | 0 | 2.61 | 0.88 | 0 | 3.64 | 0.33 | 3 | 4 | 0 |
| 2007 | 31 | 71.18 | 15.23 | 0 | 0.41 | 0 | 0.35 | 0 | 2.74 | 0.86 | 0 | 3.67 | 0.41 | 2.75 | 4 | 0 |
| 2008 | 62 | 77.01 | 12.37 | 0 | 1.00 | 0 | 0.27 | 0 | 2.69 | 0.93 | 0 | 3.79 | 0.31 | 2.75 | 4 | 0 |
| 2009 | 71 | 78.51 | 13.12 | 0 | 1.73 | 0 | 0.2 | 0 | 2.49 | 0.71 | 0 | 3.72 | 0.32 | 3 | 4 | 0 |
| 2010 | 81 | 81.46 | 11.00 | 0 | 1.25 | 0 | 0.21 | 0 | 2.65 | 0.76 | 0 | 3.46 | 0.44 | 2.5 | 4 | 0 |
| 2011 | 116 | 82.21 | 10.09 | 0 | 1.83 | 0 | 0.12 | 0 | 2.47 | 0.85 | 0 | 3.5 | 0.39 | 2.5 | 4 | 2 |
| 2012 | 152 | 80.87 | 11.58 | 0 | 1.45 | 0 | 0.28 | 0 | 2.55 | 0.93 | 0 | 3.36 | 0.43 | 2.33 | 4 | 5 |
| 2013 | 235 | 83.06 | 9.76 | 0 | 1.37 | 0 | 0.18 | 0 | 2.53 | 0.86 | 0 | 3.5 | 0.42 | 2 | 4 | 5 |
| 2014 | 175 | 84.22 | 9.52 | 0 | 1.87 | 0 | 0.16 | 0 | 2.65 | 1 | 0 | 3.35 | 0.49 | 1.6 | 4 | 3 |
| 2015 | 203 | 82.31 | 10.85 | 0 | 1.09 | 0 | 0.16 | 0 | 2.75 | 1.04 | 0 | 3.38 | 0.44 | 2.33 | 4 | 6 |
| 2016 | 257 | 83.13 | 10.82 | 0 | 1.36 | 0 | 0.25 | 0 | 2.84 | 1.1 | 0 | 3.5 | 0.42 | 2.5 | 4 | 2 |
| 2017 | 428 | 83.4 | 11.03 | 0 | 1.34 | 0 | 0.27 | 0 | 2.58 | 1.01 | 0 | 3.5 | 0.42 | 1 | 4 | 20 |
| 2018 | 434 | 83.23 | 11.33 | 0 | 1.54 | 0 | 0.47 | 0 | 2.71 | 0.88 | 0 | 3.46 | 0.4 | 2.33 | 4 | 1 |
| 2019 | 460 | 83.81 | 11.3 | 0 | 1.61 | 0 | 0.35 | 0 | 2.81 | 0.87 | 0 | 3.44 | 0.39 | 2.5 | 4 | 1 |
| 2020 | 422 | 83.37 | 10.39 | 0 | 1.48 | 0 | 0.38 | 0 | 2.8 | 0.82 | 0 | 3.35 | 0.41 | 2.33 | 4 | 0 |
| 2021 | 479 | 83.44 | 11.14 | 0 | 1.23 | 0 | 0.43 | 0 | 2.84 | 0.9 | 0 | 3.36 | 0.41 | 2.33 | 4 | 0 |
| 2022 | 582 | 84.27 | 10.41 | 0 | 1.43 | 0 | 0.46 | 0 | 2.84 | 0.89 | 0 | 3.46 | 0.41 | 2.33 | 4 | 1 |
| 2023 | 793 | 84.27 | 10.71 | 0 | 1.57 | 0 | 0.42 | 0 | 2.93 | 0.98 | 0 | 3.4 | 0.41 | 2 | 4 | 5 |
| 2024 | 1070 | 83.06 | 11.45 | 0 | 1.27 | 0 | 0.35 | 0 | 2.94 | 0.91 | 0 | 3.41 | 0.4 | 2.25 | 4 | 1 |
| **Overall** | **6152** | **83.1** | **11.11** | **0** | **1.39** | **0** | **0.34** | **0** | **2.79** | **0.93** | **0** | **3.43** | **0.41** | **1** | **4** | **52** |

| Metric | Value |
| --- | --- |
| Total Study Period | 2003 - 2024 |
| Study Years | 22 years |
| Total Cases | 6152 cases |
| Mean Age (Overall) | 83.1 years (SD# 11.1) |
| Age Range | 22.25 - 105.72 years |
| Female#Male Ratio (Overall) | 1.39 (F#M) |
| Sex Distribution | Female# 3582, Male# 2570 |
| French#Dutch Ratio (Overall) | 0.34 (F#D) |
| Language Distribution | French# 1556, Dutch# 4596 |
| Mean Number of Conditions (Overall) | 2.79 (SD# 0.93) |
| Conditions Range | 0 - 8 |
| Mean Severity Index (Overall) | 3.43 (SD# 0.41) |
| Severity Index Range | 1 - 4 |
| Year with Highest Cases | 2024 (n = 1070) |
| Year with Lowest Cases | 2003 (n = 15) |
| Missing Data - Age | 0 (0%) |
| Missing Data - Sex | 0 (0%) |
| Missing Data - Language | 0 (0%) |
| Missing Data - Conditions | 0 (0%) |
| Missing Data - Severity | 52 (0.8%) |

# Appendix 3. Optimal number of clusters

We first assessed clustering tendency using the Hopkins statistic, which was 0.18, indicating that the data were not uniformly random and that some clustering structure was present. To determine the optimal number of clusters (*k*), we applied multiple indices. The average silhouette width across *k* = 2–10 remained relatively low (0.20–0.24), suggesting that disease patterns in this cohort were only weakly separated, which is consistent with the expected overlap of multimorbidity profiles. The maximum silhouette width was observed at *k* = 10 (0.24), whereas *k* = 5 had a silhouette width of 0.20. Despite the modest silhouette widths, cluster stability analysis using bootstrap (b=1000) resampling showed strong robustness of the five-cluster solution (mean Jaccard similarity coefficients# 0.81–0.90 across clusters). Taken together, the balance of interpretability in the radar plots, moderate-to-high stability, and clinical plausibility supported the use of a five-cluster solution for subsequent analyses.

## All cases

| Hopkins statistic# 0.1829199 | |
| --- | --- |
|  |  |
|  | |

## Female cases

| Hopkins statistic# 0.1840787 | |
| --- | --- |
|  |  |
|  | |

## Male cases

| Hopkins statistic# 0.1910118 | |
| --- | --- |
|  |  |
|  | |

## Pairwise correlations across conditions and clustering visualisation, by sex

To visualize pairwise relationships among the 19 health conditions, we constructed sex-specific Pairwise Pearson correlation networks based on condition severity scores (range 0–4). correlation coefficients were calculated separately for females and males. Correlations with absolute values ≥ 0.03 were retained as edges connecting condition nodes. The figure bloew shows the pairwise correlation networks across conditions, by sex. These visualizations highlight the pattern and intensity of associations among conditions within each sex. In female patients, cardiac conditions demonstrated positive correlations with trauma (r=0.161) and genitourinary conditions (r=0.178), while musculoskeletal and eye/ear/balance conditions were positively associated (r=0.145). Male patients showed similar cardiac-trauma (r=0.146) and cardiac-genitourinary (r=0.159) positive correlations, with additional positive association between musculoskeletal and eye/ear/balance conditions (r=0.158). Notable negative correlations were observed between neoplasms and musculoskeletal conditions in both females (r=-0.256) and males (r=-0.235). Males exhibited stronger negative correlations between neurological and cardiac conditions (r=-0.256) compared to females (r=-0.204). COVID 19-19 showed minimal correlations with other conditions in both sexes, with the strongest being a positive association with infections in both male (r=0.099) and females (r=0.180). These pairwise correlations represent individual condition relationships, whereas the clustering approach captures broader multimorbidity constellations, suggesting that patient profiles are better understood as galaxies of conditions with multiple weak associations rather than tightly correlated disease groups.

**Conditions severity correlation networks by sex and cluster**

**Female correlations matrix**

|  | musculoskeletal | neurological | infections | COVID 19 | hematological | respiratory | cardiac | vascular | Endocrine systemic diseases | dermatological | trauma | genitourinary | gastrointestinal | Eye ear balance | psychiatric | Dementia memory disorders | Symptoms and complaints | congenital | neoplasms |
| --- | --- | --- | --- | --- | --- | --- | --- | --- | --- | --- | --- | --- | --- | --- | --- | --- | --- | --- | --- |
| musculoskeletal |  | -0.142 | -0.072 | 0.012 | -0.041 | -0.148 | -0.134 | -0.060 | -0.062 | -0.015 | -0.105 | -0.071 | -0.017 | 0.145 | 0.038 | 0.007 | -0.012 | -0.031 | -0.256 |
| neurological | -0.142 |  | -0.020 | -0.026 | -0.046 | -0.142 | -0.204 | -0.080 | -0.003 | 0.024 | -0.083 | -0.083 | -0.033 | -0.085 | 0.005 | 0.015 | -0.032 | 0.020 | -0.024 |
| infections | -0.072 | -0.020 |  | 0.180 | 0.023 | -0.014 | -0.037 | -0.032 | -0.015 | 0.010 | -0.032 | -0.038 | 0.027 | -0.057 | -0.033 | -0.022 | 0.008 | -0.007 | 0.016 |
| COVID 19 | 0.012 | -0.026 | 0.180 |  | -0.011 | -0.013 | 0.016 | -0.021 | -0.009 | 0.004 | -0.008 | 0.007 | 0.000 | -0.012 | -0.025 | -0.017 | -0.008 | -0.003 | 0.020 |
| hematological | -0.041 | -0.046 | 0.023 | -0.011 |  | -0.013 | 0.013 | -0.003 | 0.019 | 0.007 | 0.014 | 0.025 | 0.016 | -0.051 | -0.027 | -0.025 | 0.008 | -0.006 | -0.045 |
| respiratory | -0.148 | -0.142 | -0.014 | -0.013 | -0.013 |  | 0.033 | 0.004 | -0.019 | -0.007 | -0.021 | -0.069 | -0.031 | -0.155 | -0.068 | -0.082 | -0.084 | -0.003 | 0.059 |
| cardiac | -0.134 | -0.204 | -0.037 | 0.016 | 0.013 | 0.033 |  | -0.004 | 0.040 | -0.024 | 0.161 | 0.178 | -0.085 | -0.142 | -0.164 | -0.100 | -0.089 | -0.031 | -0.049 |
| vascular | -0.060 | -0.080 | -0.032 | -0.021 | -0.003 | 0.004 | -0.004 |  | 0.072 | -0.018 | -0.004 | 0.007 | -0.028 | -0.071 | -0.037 | -0.039 | -0.027 | -0.011 | -0.048 |
| endocrine_systemic_diseases | -0.062 | -0.003 | -0.015 | -0.009 | 0.019 | -0.019 | 0.040 | 0.072 |  | -0.020 | 0.027 | 0.044 | -0.017 | -0.077 | -0.038 | -0.015 | -0.024 | -0.012 | -0.047 |
| dermatological | -0.015 | 0.024 | 0.010 | 0.004 | 0.007 | -0.007 | -0.024 | -0.018 | -0.020 |  | -0.003 | -0.001 | -0.012 | -0.060 | -0.023 | -0.032 | 0.016 | -0.009 | -0.045 |
| trauma | -0.105 | -0.083 | -0.032 | -0.008 | 0.014 | -0.021 | 0.161 | -0.004 | 0.027 | -0.003 |  | -0.072 | -0.046 | -0.099 | -0.083 | -0.048 | -0.067 | 0.011 | -0.072 |
| genitourinary | -0.071 | -0.083 | -0.038 | 0.007 | 0.025 | -0.069 | 0.178 | 0.007 | 0.044 | -0.001 | -0.072 |  | 0.007 | -0.068 | -0.095 | -0.031 | -0.031 | -0.016 | -0.057 |
| gastrointestinal | -0.017 | -0.033 | 0.027 | 0.000 | 0.016 | -0.031 | -0.085 | -0.028 | -0.017 | -0.012 | -0.046 | 0.007 |  | -0.062 | -0.013 | -0.009 | -0.009 | -0.014 | -0.078 |
| eye_ear_balance | 0.145 | -0.085 | -0.057 | -0.012 | -0.051 | -0.155 | -0.142 | -0.071 | -0.077 | -0.060 | -0.099 | -0.068 | -0.062 |  | -0.013 | -0.015 | 0.012 | 0.002 | -0.138 |
| psychiatric | 0.038 | 0.005 | -0.033 | -0.025 | -0.027 | -0.068 | -0.164 | -0.037 | -0.038 | -0.023 | -0.083 | -0.095 | -0.013 | -0.013 |  | -0.012 | 0.016 | 0.033 | -0.107 |
| dementia_memory_disorders | 0.007 | 0.015 | -0.022 | -0.017 | -0.025 | -0.082 | -0.100 | -0.039 | -0.015 | -0.032 | -0.048 | -0.031 | -0.009 | -0.015 | -0.012 |  | 0.030 | -0.009 | -0.051 |
| symptoms_and_complaints | -0.012 | -0.032 | 0.008 | -0.008 | 0.008 | -0.084 | -0.089 | -0.027 | -0.024 | 0.016 | -0.067 | -0.031 | -0.009 | 0.012 | 0.016 | 0.030 |  | 0.044 | -0.098 |
| congenital | -0.031 | 0.020 | -0.007 | -0.003 | -0.006 | -0.003 | -0.031 | -0.011 | -0.012 | -0.009 | 0.011 | -0.016 | -0.014 | 0.002 | 0.033 | -0.009 | 0.044 |  | -0.022 |
| neoplasms | -0.256 | -0.024 | 0.016 | 0.020 | -0.045 | 0.059 | -0.049 | -0.048 | -0.047 | -0.045 | -0.072 | -0.057 | -0.078 | -0.138 | -0.107 | -0.051 | -0.098 | -0.022 |  |

**Male correlatons matrix**

|  | musculoskeletal | neurological | infections | COVID 19 | hematological | respiratory | cardiac | vascular | Endocrine systemic diseases | dermatological | trauma | genitourinary | gastrointestinal | Eye ear balance | psychiatric | Dementia memory disorders | Symptoms and complaints | congenital | neoplasms |
| --- | --- | --- | --- | --- | --- | --- | --- | --- | --- | --- | --- | --- | --- | --- | --- | --- | --- | --- | --- |
| musculoskeletal |  | -0.075 | -0.053 | -0.037 | -0.029 | -0.143 | -0.092 | -0.061 | -0.032 | -0.008 | -0.064 | -0.071 | -0.045 | 0.158 | 0.085 | 0.013 | 0.039 | 0.026 | -0.235 |
| neurological | -0.075 |  | -0.009 | -0.026 | -0.032 | -0.201 | -0.256 | -0.082 | -0.035 | 0.080 | -0.130 | -0.138 | -0.058 | -0.020 | 0.028 | 0.024 | 0.026 | 0.036 | -0.122 |
| infections | -0.053 | -0.009 |  | 0.099 | 0.019 | -0.048 | -0.056 | -0.021 | 0.033 | 0.005 | -0.030 | 0.004 | 0.008 | -0.064 | -0.012 | -0.034 | -0.008 | -0.004 | -0.013 |
| COVID 19 | -0.037 | -0.026 | 0.099 |  | -0.011 | 0.012 | -0.020 | -0.008 | -0.016 | 0.007 | -0.015 | 0.003 | 0.001 | -0.007 | -0.022 | -0.016 | -0.018 | -0.001 | -0.001 |
| hematological | -0.029 | -0.032 | 0.019 | -0.011 |  | -0.006 | 0.009 | -0.026 | -0.020 | 0.016 | 0.012 | 0.029 | 0.024 | -0.027 | -0.028 | -0.031 | -0.036 | -0.003 | -0.040 |
| respiratory | -0.143 | -0.201 | -0.048 | 0.012 | -0.006 |  | 0.032 | -0.055 | -0.040 | -0.019 | -0.030 | -0.127 | -0.050 | -0.158 | -0.099 | -0.104 | -0.071 | -0.012 | 0.041 |
| cardiac | -0.092 | -0.256 | -0.056 | -0.020 | 0.009 | 0.032 |  | -0.020 | -0.009 | -0.034 | 0.146 | 0.159 | -0.099 | -0.098 | -0.119 | -0.105 | -0.090 | -0.017 | -0.122 |
| vascular | -0.061 | -0.082 | -0.021 | -0.008 | -0.026 | -0.055 | -0.020 |  | 0.038 | -0.004 | 0.006 | -0.006 | -0.052 | -0.033 | -0.044 | -0.033 | -0.056 | -0.007 | -0.040 |
| endocrine_systemic_diseases | -0.032 | -0.035 | 0.033 | -0.016 | -0.020 | -0.040 | -0.009 | 0.038 |  | 0.010 | 0.012 | 0.025 | 0.014 | -0.026 | -0.016 | -0.015 | -0.010 | -0.006 | -0.114 |
| dermatological | -0.008 | 0.080 | 0.005 | 0.007 | 0.016 | -0.019 | -0.034 | -0.004 | 0.010 |  | -0.021 | -0.013 | -0.003 | -0.050 | -0.043 | -0.025 | -0.007 | -0.004 | -0.063 |
| trauma | -0.064 | -0.130 | -0.030 | -0.015 | 0.012 | -0.030 | 0.146 | 0.006 | 0.012 | -0.021 |  | -0.104 | 0.016 | -0.096 | -0.059 | -0.044 | -0.057 | -0.006 | -0.060 |
| genitourinary | -0.071 | -0.138 | 0.004 | 0.003 | 0.029 | -0.127 | 0.159 | -0.006 | 0.025 | -0.013 | -0.104 |  | 0.028 | -0.040 | -0.059 | -0.061 | -0.029 | -0.009 | -0.056 |
| gastrointestinal | -0.045 | -0.058 | 0.008 | 0.001 | 0.024 | -0.050 | -0.099 | -0.052 | 0.014 | -0.003 | 0.016 | 0.028 |  | -0.073 | 0.010 | -0.042 | -0.003 | -0.006 | -0.073 |
| eye_ear_balance | 0.158 | -0.020 | -0.064 | -0.007 | -0.027 | -0.158 | -0.098 | -0.033 | -0.026 | -0.050 | -0.096 | -0.040 | -0.073 |  | 0.030 | 0.017 | 0.028 | 0.035 | -0.183 |
| psychiatric | 0.085 | 0.028 | -0.012 | -0.022 | -0.028 | -0.099 | -0.119 | -0.044 | -0.016 | -0.043 | -0.059 | -0.059 | 0.010 | 0.030 |  | 0.013 | 0.085 | -0.006 | -0.131 |
| dementia_memory_disorders | 0.013 | 0.024 | -0.034 | -0.016 | -0.031 | -0.104 | -0.105 | -0.033 | -0.015 | -0.025 | -0.044 | -0.061 | -0.042 | 0.017 | 0.013 |  | 0.055 | -0.004 | -0.056 |
| symptoms_and_complaints | 0.039 | 0.026 | -0.008 | -0.018 | -0.036 | -0.071 | -0.090 | -0.056 | -0.010 | -0.007 | -0.057 | -0.029 | -0.003 | 0.028 | 0.085 | 0.055 |  | -0.005 | -0.107 |
| congenital | 0.026 | 0.036 | -0.004 | -0.001 | -0.003 | -0.012 | -0.017 | -0.007 | -0.006 | -0.004 | -0.006 | -0.009 | -0.006 | 0.035 | -0.006 | -0.004 | -0.005 |  | -0.015 |
| neoplasms | -0.235 | -0.122 | -0.013 | -0.001 | -0.040 | 0.041 | -0.122 | -0.040 | -0.114 | -0.063 | -0.060 | -0.056 | -0.073 | -0.183 | -0.131 | -0.056 | -0.107 | -0.015 |  |

# Appendix 4. Negative binomial regression of each conditions (model 0)

|  | Female | | | |  | Male | | | |
| --- | --- | --- | --- | --- | --- | --- | --- | --- | --- |
| **Main effects** | estimate | conf.low | conf.high | p.value |  | estimate | conf.low | conf.high | p.value |
| (Intercept) | -33.768 | -86.421 | 18.911 | 0.173 |  | -22.29 | -90.818 | 46.286 | 0.484 |
| musculoskeletal | -11.723 | -46.752 | 23.316 | 0.469 |  | 5.496 | -47.427 | 58.527 | 0.82 |
| Year | 0.012 | -0.015 | 0.038 | 0.349 |  | 0.006 | -0.028 | 0.04 | 0.72 |
| neurological | 10.377 | -29.091 | 49.875 | 0.577 |  | 12.753 | -34.194 | 59.779 | 0.557 |
| infections | 57.385 | -47.939 | 162.598 | 0.243 |  | -32.276 | -111.531 | 47.739 | 0.388 |
| COVID 19 | -289.325 | -1133.966 | 573.22 | 0.473 |  | 520.631 | -529.361 | 1626.546 | 0.296 |
| hematological | 66.026 | -70.087 | 201.773 | 0.297 |  | -82.757 | -210.253 | 44.3 | 0.18 |
| respiratory | 34.027 | -9.016 | 77.123 | 0.094 |  | -13.721 | -59.949 | 32.506 | 0.524 |
| cardiac | 38.909 | 2.366 | 75.501 | 0.022 |  | 2.086 | -40.527 | 44.766 | 0.915 |
| vascular | 47.896 | -10.462 | 106.498 | 0.081 |  | 37.371 | -31.543 | 106.612 | 0.239 |
| endocrine_systemic_diseases | 21.896 | -29.514 | 73.376 | 0.366 |  | 12.831 | -46.648 | 72.466 | 0.642 |
| dermatological | 73.884 | 11.743 | 136.527 | 0.008 |  | -72.753 | -164.946 | 18.91 | 0.103 |
| trauma | -33.013 | -110.766 | 44.845 | 0.364 |  | 6.38 | -90.788 | 103.652 | 0.884 |
| genitourinary | -10.514 | -60.902 | 40.026 | 0.655 |  | -28.393 | -81.224 | 24.598 | 0.249 |
| gastrointestinal | -0.238 | -51.457 | 51.134 | 0.992 |  | 58.162 | -5.276 | 121.779 | 0.046 |
| eye_ear_balance | 13.269 | -26.565 | 53.133 | 0.465 |  | -6.645 | -65.383 | 52.226 | 0.799 |
| psychiatric | 24.858 | -24.71 | 74.594 | 0.284 |  | 126.025 | 48.656 | 204.267 | 0 |
| dementia_memory_disorders | 74.342 | 4.447 | 145.069 | 0.02 |  | 133.402 | 38.392 | 228.954 | 0.003 |
| symptoms_and_complaints | 3.748 | -52.229 | 59.889 | 0.887 |  | 64.654 | -33.557 | 165.764 | 0.135 |
| congenital | -2841.206 | -6800.412 | 898.787 | 0.111 |  | -2.569 | -5.646 | -0.205 | 0.037 |
| neoplasms | -12.728 | -52.342 | 26.922 | 0.496 |  | -17.252 | -63.154 | 28.639 | 0.421 |
| **Interaction terms** |  |  |  |  |  |  |  |  |  |
| musculoskeletal x Year | 0.006 | -0.011 | 0.023 | 0.464 |  | -0.003 | -0.029 | 0.024 | 0.822 |
| Year x neurological | -0.005 | -0.025 | 0.014 | 0.563 |  | -0.006 | -0.03 | 0.017 | 0.549 |
| Year x infections | -0.029 | -0.081 | 0.024 | 0.24 |  | 0.016 | -0.024 | 0.055 | 0.393 |
| Year x COVID 19 19 | 0.143 | -0.284 | 0.561 | 0.474 |  | -0.258 | -0.805 | 0.262 | 0.296 |
| Year x hematological | -0.033 | -0.1 | 0.035 | 0.296 |  | 0.041 | -0.022 | 0.104 | 0.181 |
| Year x respiratory | -0.017 | -0.038 | 0.004 | 0.09 |  | 0.007 | -0.016 | 0.03 | 0.531 |
| Year x cardiac | -0.019 | -0.037 | -0.001 | 0.022 |  | -0.001 | -0.022 | 0.02 | 0.929 |
| Year x vascular | -0.024 | -0.053 | 0.005 | 0.079 |  | -0.019 | -0.053 | 0.015 | 0.235 |
| Year x endocrine_systemic_diseases | -0.011 | -0.037 | 0.014 | 0.354 |  | -0.007 | -0.036 | 0.023 | 0.63 |
| Year x dermatological | -0.037 | -0.068 | -0.006 | 0.008 |  | 0.036 | -0.009 | 0.082 | 0.103 |
| Year x trauma | 0.016 | -0.022 | 0.055 | 0.367 |  | -0.003 | -0.051 | 0.045 | 0.885 |
| Year x genitourinary | 0.005 | -0.02 | 0.03 | 0.661 |  | 0.014 | -0.012 | 0.04 | 0.248 |
| Year x gastrointestinal | 0.000 | -0.025 | 0.025 | 0.998 |  | -0.029 | -0.061 | 0.002 | 0.045 |
| Year x eye_ear_balance | -0.006 | -0.026 | 0.013 | 0.478 |  | 0.004 | -0.026 | 0.033 | 0.782 |
| Year x psychiatric | -0.013 | -0.037 | 0.012 | 0.274 |  | -0.063 | -0.101 | -0.024 | 0 |
| Year x dementia_memory_disorders | -0.037 | -0.072 | -0.002 | 0.019 |  | -0.066 | -0.113 | -0.019 | 0.003 |
| Year x symptoms_and_complaints | -0.002 | -0.03 | 0.026 | 0.88 |  | -0.032 | -0.082 | 0.017 | 0.134 |
| Year x congenital | 1.404 | -0.444 | 3.36 | 0.111 |  |  |  |  |  |
| Year x neoplasms | 0.006 | -0.013 | 0.026 | 0.507 |  | 0.008 | -0.014 | 0.031 | 0.424 |

# Appendix 5. Negative binomial regression of each conditions with splines (model 0)

#

|  |  | Female | | |  | Male | | |
| --- | --- | --- | --- | --- | --- | --- | --- | --- |
| Year | condition | Predicted rate | 95%CI lower | 95%CI upper |  |  |  |  |
| 2003 | musculoskeletal | 0.01140 | 0.00432 | 0.03010 |  | 0.01466 | 0.00385 | 0.05587 |
| 2004 | musculoskeletal | 0.01256 | 0.00543 | 0.02905 |  | 0.01514 | 0.00476 | 0.04817 |
| 2005 | musculoskeletal | 0.01382 | 0.00679 | 0.02813 |  | 0.01562 | 0.00584 | 0.04178 |
| 2006 | musculoskeletal | 0.01519 | 0.00842 | 0.02740 |  | 0.01611 | 0.00709 | 0.03661 |
| 2007 | musculoskeletal | 0.01667 | 0.01033 | 0.02692 |  | 0.01660 | 0.00847 | 0.03255 |
| 2008 | musculoskeletal | 0.01826 | 0.01246 | 0.02676 |  | 0.01710 | 0.00991 | 0.02951 |
| 2009 | musculoskeletal | 0.01994 | 0.01472 | 0.02702 |  | 0.01758 | 0.01127 | 0.02744 |
| 2010 | musculoskeletal | 0.02171 | 0.01694 | 0.02784 |  | 0.01806 | 0.01241 | 0.02629 |
| 2011 | musculoskeletal | 0.02355 | 0.01895 | 0.02928 |  | 0.01852 | 0.01323 | 0.02592 |
| 2012 | musculoskeletal | 0.02544 | 0.02071 | 0.03124 |  | 0.01895 | 0.01378 | 0.02605 |
| 2013 | musculoskeletal | 0.02734 | 0.02237 | 0.03342 |  | 0.01935 | 0.01424 | 0.02629 |
| 2014 | musculoskeletal | 0.02923 | 0.02410 | 0.03546 |  | 0.01971 | 0.01476 | 0.02632 |
| 2015 | musculoskeletal | 0.03107 | 0.02602 | 0.03709 |  | 0.02002 | 0.01544 | 0.02596 |
| 2016 | musculoskeletal | 0.03281 | 0.02806 | 0.03836 |  | 0.02028 | 0.01619 | 0.02541 |
| 2017 | musculoskeletal | 0.03436 | 0.02954 | 0.03996 |  | 0.02047 | 0.01648 | 0.02544 |
| 2018 | musculoskeletal | 0.03542 | 0.02979 | 0.04211 |  | 0.02054 | 0.01597 | 0.02642 |
| 2019 | musculoskeletal | 0.03563 | 0.02942 | 0.04315 |  | 0.02043 | 0.01543 | 0.02704 |
| 2020 | musculoskeletal | 0.03466 | 0.02895 | 0.04150 |  | 0.02008 | 0.01549 | 0.02602 |
| 2021 | musculoskeletal | 0.03252 | 0.02724 | 0.03881 |  | 0.01952 | 0.01538 | 0.02476 |
| 2022 | musculoskeletal | 0.02997 | 0.02422 | 0.03707 |  | 0.01909 | 0.01438 | 0.02533 |
| 2023 | musculoskeletal | 0.02780 | 0.02313 | 0.03342 |  | 0.01918 | 0.01491 | 0.02466 |
| 2024 | musculoskeletal | 0.02636 | 0.02071 | 0.03356 |  | 0.01997 | 0.01442 | 0.02765 |
| 2003 | neurological | 0.01038 | 0.00496 | 0.02174 |  | 0.01022 | 0.00356 | 0.02933 |
| 2004 | neurological | 0.01109 | 0.00587 | 0.02096 |  | 0.01094 | 0.00440 | 0.02719 |
| 2005 | neurological | 0.01185 | 0.00692 | 0.02029 |  | 0.01170 | 0.00540 | 0.02532 |
| 2006 | neurological | 0.01263 | 0.00808 | 0.01977 |  | 0.01248 | 0.00656 | 0.02376 |
| 2007 | neurological | 0.01345 | 0.00930 | 0.01944 |  | 0.01327 | 0.00782 | 0.02253 |
| 2008 | neurological | 0.01428 | 0.01052 | 0.01937 |  | 0.01406 | 0.00911 | 0.02168 |
| 2009 | neurological | 0.01511 | 0.01165 | 0.01959 |  | 0.01481 | 0.01032 | 0.02125 |
| 2010 | neurological | 0.01592 | 0.01259 | 0.02013 |  | 0.01550 | 0.01131 | 0.02125 |
| 2011 | neurological | 0.01670 | 0.01333 | 0.02091 |  | 0.01610 | 0.01202 | 0.02157 |
| 2012 | neurological | 0.01742 | 0.01395 | 0.02175 |  | 0.01659 | 0.01250 | 0.02201 |
| 2013 | neurological | 0.01806 | 0.01452 | 0.02246 |  | 0.01693 | 0.01286 | 0.02228 |
| 2014 | neurological | 0.01860 | 0.01512 | 0.02289 |  | 0.01709 | 0.01319 | 0.02214 |
| 2015 | neurological | 0.01902 | 0.01576 | 0.02296 |  | 0.01705 | 0.01352 | 0.02151 |
| 2016 | neurological | 0.01930 | 0.01632 | 0.02281 |  | 0.01680 | 0.01374 | 0.02055 |
| 2017 | neurological | 0.01944 | 0.01648 | 0.02293 |  | 0.01634 | 0.01346 | 0.01985 |
| 2018 | neurological | 0.01959 | 0.01624 | 0.02364 |  | 0.01575 | 0.01259 | 0.01971 |
| 2019 | neurological | 0.01996 | 0.01628 | 0.02447 |  | 0.01511 | 0.01178 | 0.01938 |
| 2020 | neurological | 0.02074 | 0.01720 | 0.02500 |  | 0.01449 | 0.01149 | 0.01828 |
| 2021 | neurological | 0.02183 | 0.01827 | 0.02609 |  | 0.01399 | 0.01126 | 0.01737 |
| 2022 | neurological | 0.02165 | 0.01748 | 0.02682 |  | 0.01375 | 0.01067 | 0.01772 |
| 2023 | neurological | 0.01850 | 0.01534 | 0.02231 |  | 0.01396 | 0.01120 | 0.01739 |
| 2024 | neurological | 0.01316 | 0.01025 | 0.01689 |  | 0.01469 | 0.01101 | 0.01960 |
| 2003 | infections | 0.02264 | 0.00061 | 0.83474 |  | 0.01131 | 0.00256 | 0.04998 |
| 2004 | infections | 0.02154 | 0.00099 | 0.46693 |  | 0.01199 | 0.00343 | 0.04196 |
| 2005 | infections | 0.02052 | 0.00159 | 0.26480 |  | 0.01269 | 0.00451 | 0.03566 |
| 2006 | infections | 0.01960 | 0.00249 | 0.15405 |  | 0.01336 | 0.00577 | 0.03092 |
| 2007 | infections | 0.01879 | 0.00379 | 0.09316 |  | 0.01397 | 0.00706 | 0.02764 |
| 2008 | infections | 0.01812 | 0.00551 | 0.05959 |  | 0.01447 | 0.00813 | 0.02576 |
| 2009 | infections | 0.01760 | 0.00749 | 0.04136 |  | 0.01483 | 0.00873 | 0.02518 |
| 2010 | infections | 0.01723 | 0.00913 | 0.03250 |  | 0.01498 | 0.00881 | 0.02546 |
| 2011 | infections | 0.01703 | 0.00965 | 0.03006 |  | 0.01489 | 0.00856 | 0.02592 |
| 2012 | infections | 0.01702 | 0.00920 | 0.03150 |  | 0.01454 | 0.00817 | 0.02587 |
| 2013 | infections | 0.01722 | 0.00868 | 0.03417 |  | 0.01390 | 0.00778 | 0.02484 |
| 2014 | infections | 0.01766 | 0.00860 | 0.03626 |  | 0.01299 | 0.00741 | 0.02276 |
| 2015 | infections | 0.01839 | 0.00920 | 0.03675 |  | 0.01184 | 0.00702 | 0.01995 |
| 2016 | infections | 0.01947 | 0.01063 | 0.03564 |  | 0.01049 | 0.00642 | 0.01714 |
| 2017 | infections | 0.02087 | 0.01257 | 0.03465 |  | 0.00910 | 0.00541 | 0.01530 |
| 2018 | infections | 0.02213 | 0.01350 | 0.03627 |  | 0.00800 | 0.00437 | 0.01464 |
| 2019 | infections | 0.02254 | 0.01325 | 0.03835 |  | 0.00745 | 0.00387 | 0.01436 |
| 2020 | infections | 0.02144 | 0.01307 | 0.03516 |  | 0.00769 | 0.00425 | 0.01391 |
| 2021 | infections | 0.01881 | 0.01248 | 0.02833 |  | 0.00903 | 0.00571 | 0.01427 |
| 2022 | infections | 0.01610 | 0.01025 | 0.02527 |  | 0.01170 | 0.00756 | 0.01813 |
| 2023 | infections | 0.01446 | 0.00974 | 0.02147 |  | 0.01597 | 0.01098 | 0.02323 |
| 2024 | infections | 0.01420 | 0.00892 | 0.02261 |  | 0.02205 | 0.01281 | 0.03796 |
| 2011 | COVID 19 | 0.02020 | 0.00344 | 0.11857 |  | 0.00428 | 0.00035 | 0.05259 |
| 2012 | COVID 19 | 0.00008 | 0.00000 | 0.53689 |  | 0.00002 | 0.00000 | 1.93342 |
| 2013 | COVID 19 | 0.00000 | 0.00000 | 2.95483 |  | 0.00000 | 0.00000 | 61.97738 |
| 2014 | COVID 19 | 0.00000 | 0.00000 | 8.56728 |  | 0.00000 | 0.00000 | 606.77394 |
| 2015 | COVID 19 | 0.00000 | 0.00000 | 11.88515 |  | 0.00000 | 0.00000 | 1539.47814 |
| 2016 | COVID 19 | 0.00000 | 0.00000 | 7.34186 |  | 0.00000 | 0.00000 | 888.20145 |
| 2017 | COVID 19 | 0.00000 | 0.00000 | 2.07591 |  | 0.00000 | 0.00000 | 120.17174 |
| 2018 | COVID 19 | 0.00004 | 0.00000 | 0.40025 |  | 0.00008 | 0.00000 | 7.25679 |
| 2019 | COVID 19 | 0.00143 | 0.00002 | 0.08790 |  | 0.00306 | 0.00002 | 0.44572 |
| 2020 | COVID 19 | 0.01638 | 0.00589 | 0.04559 |  | 0.02809 | 0.00899 | 0.08780 |
| 2021 | COVID 19 | 0.03157 | 0.01579 | 0.06311 |  | 0.03050 | 0.01314 | 0.07081 |
| 2022 | COVID 19 | 0.02340 | 0.01170 | 0.04683 |  | 0.01089 | 0.00495 | 0.02397 |
| 2023 | COVID 19 | 0.02221 | 0.00859 | 0.05747 |  | 0.00549 | 0.00135 | 0.02226 |
| 2024 | COVID 19 | 0.05771 | 0.00912 | 0.36503 |  | 0.00974 | 0.00084 | 0.11312 |
| 2003 | hematological | 0.49644 | 0.00014 | 1756.09645 |  | 0.00978 | 0.00066 | 0.14578 |
| 2004 | hematological | 0.34256 | 0.00028 | 425.39716 |  | 0.01018 | 0.00104 | 0.09993 |
| 2005 | hematological | 0.23744 | 0.00054 | 105.17091 |  | 0.01060 | 0.00161 | 0.06981 |
| 2006 | hematological | 0.16608 | 0.00102 | 27.04602 |  | 0.01100 | 0.00240 | 0.05035 |
| 2007 | hematological | 0.11776 | 0.00188 | 7.37648 |  | 0.01138 | 0.00340 | 0.03817 |
| 2008 | hematological | 0.08502 | 0.00332 | 2.17728 |  | 0.01172 | 0.00442 | 0.03111 |
| 2009 | hematological | 0.06279 | 0.00555 | 0.71095 |  | 0.01200 | 0.00516 | 0.02789 |
| 2010 | hematological | 0.04766 | 0.00861 | 0.26363 |  | 0.01220 | 0.00542 | 0.02745 |
| 2011 | hematological | 0.03733 | 0.01209 | 0.11530 |  | 0.01230 | 0.00531 | 0.02852 |
| 2012 | hematological | 0.03033 | 0.01445 | 0.06363 |  | 0.01229 | 0.00509 | 0.02970 |
| 2013 | hematological | 0.02566 | 0.01373 | 0.04796 |  | 0.01216 | 0.00496 | 0.02981 |
| 2014 | hematological | 0.02272 | 0.01144 | 0.04512 |  | 0.01188 | 0.00501 | 0.02817 |
| 2015 | hematological | 0.02114 | 0.01013 | 0.04410 |  | 0.01146 | 0.00530 | 0.02481 |
| 2016 | hematological | 0.02077 | 0.01045 | 0.04130 |  | 0.01091 | 0.00577 | 0.02063 |
| 2017 | hematological | 0.02156 | 0.01234 | 0.03768 |  | 0.01030 | 0.00602 | 0.01764 |
| 2018 | hematological | 0.02329 | 0.01442 | 0.03762 |  | 0.01001 | 0.00563 | 0.01777 |
| 2019 | hematological | 0.02568 | 0.01561 | 0.04224 |  | 0.01045 | 0.00547 | 0.01995 |
| 2020 | hematological | 0.02835 | 0.01755 | 0.04580 |  | 0.01225 | 0.00669 | 0.02243 |
| 2021 | hematological | 0.03038 | 0.01995 | 0.04626 |  | 0.01621 | 0.00971 | 0.02704 |
| 2022 | hematological | 0.02924 | 0.01811 | 0.04721 |  | 0.02095 | 0.01170 | 0.03751 |
| 2023 | hematological | 0.02311 | 0.01524 | 0.03502 |  | 0.02203 | 0.01294 | 0.03750 |
| 2024 | hematological | 0.01460 | 0.00850 | 0.02505 |  | 0.01726 | 0.00891 | 0.03344 |
| 2003 | respiratory | 0.01027 | 0.00374 | 0.02823 |  | 0.01454 | 0.00530 | 0.03988 |
| 2004 | respiratory | 0.01112 | 0.00465 | 0.02661 |  | 0.01411 | 0.00591 | 0.03369 |
| 2005 | respiratory | 0.01203 | 0.00575 | 0.02519 |  | 0.01371 | 0.00655 | 0.02868 |
| 2006 | respiratory | 0.01299 | 0.00703 | 0.02400 |  | 0.01335 | 0.00721 | 0.02473 |
| 2007 | respiratory | 0.01398 | 0.00847 | 0.02307 |  | 0.01305 | 0.00784 | 0.02172 |
| 2008 | respiratory | 0.01497 | 0.00998 | 0.02246 |  | 0.01282 | 0.00840 | 0.01956 |
| 2009 | respiratory | 0.01595 | 0.01143 | 0.02224 |  | 0.01267 | 0.00884 | 0.01816 |
| 2010 | respiratory | 0.01687 | 0.01269 | 0.02245 |  | 0.01262 | 0.00914 | 0.01742 |
| 2011 | respiratory | 0.01772 | 0.01363 | 0.02303 |  | 0.01268 | 0.00935 | 0.01720 |
| 2012 | respiratory | 0.01844 | 0.01430 | 0.02378 |  | 0.01287 | 0.00955 | 0.01733 |
| 2013 | respiratory | 0.01901 | 0.01481 | 0.02439 |  | 0.01320 | 0.00989 | 0.01763 |
| 2014 | respiratory | 0.01937 | 0.01526 | 0.02459 |  | 0.01372 | 0.01045 | 0.01801 |
| 2015 | respiratory | 0.01951 | 0.01569 | 0.02424 |  | 0.01445 | 0.01132 | 0.01845 |
| 2016 | respiratory | 0.01938 | 0.01602 | 0.02346 |  | 0.01546 | 0.01248 | 0.01914 |
| 2017 | respiratory | 0.01901 | 0.01586 | 0.02279 |  | 0.01670 | 0.01355 | 0.02057 |
| 2018 | respiratory | 0.01851 | 0.01511 | 0.02267 |  | 0.01772 | 0.01395 | 0.02251 |
| 2019 | respiratory | 0.01801 | 0.01444 | 0.02248 |  | 0.01784 | 0.01373 | 0.02318 |
| 2020 | respiratory | 0.01765 | 0.01435 | 0.02170 |  | 0.01647 | 0.01291 | 0.02100 |
| 2021 | respiratory | 0.01742 | 0.01424 | 0.02131 |  | 0.01390 | 0.01093 | 0.01767 |
| 2022 | respiratory | 0.01690 | 0.01326 | 0.02155 |  | 0.01211 | 0.00902 | 0.01627 |
| 2023 | respiratory | 0.01565 | 0.01266 | 0.01934 |  | 0.01270 | 0.00983 | 0.01642 |
| 2024 | respiratory | 0.01364 | 0.01027 | 0.01812 |  | 0.01722 | 0.01258 | 0.02359 |
| 2003 | cardiac | 0.01667 | 0.00613 | 0.04534 |  | 0.02223 | 0.00747 | 0.06618 |
| 2004 | cardiac | 0.01831 | 0.00772 | 0.04343 |  | 0.02241 | 0.00876 | 0.05737 |
| 2005 | cardiac | 0.02008 | 0.00966 | 0.04176 |  | 0.02261 | 0.01021 | 0.05005 |
| 2006 | cardiac | 0.02200 | 0.01198 | 0.04039 |  | 0.02282 | 0.01181 | 0.04412 |
| 2007 | cardiac | 0.02404 | 0.01466 | 0.03941 |  | 0.02307 | 0.01348 | 0.03951 |
| 2008 | cardiac | 0.02618 | 0.01761 | 0.03893 |  | 0.02337 | 0.01512 | 0.03613 |
| 2009 | cardiac | 0.02840 | 0.02065 | 0.03908 |  | 0.02372 | 0.01658 | 0.03395 |
| 2010 | cardiac | 0.03067 | 0.02351 | 0.04001 |  | 0.02415 | 0.01772 | 0.03292 |
| 2011 | cardiac | 0.03293 | 0.02596 | 0.04177 |  | 0.02467 | 0.01851 | 0.03286 |
| 2012 | cardiac | 0.03512 | 0.02798 | 0.04409 |  | 0.02528 | 0.01912 | 0.03343 |
| 2013 | cardiac | 0.03720 | 0.02977 | 0.04649 |  | 0.02603 | 0.01979 | 0.03423 |
| 2014 | cardiac | 0.03909 | 0.03156 | 0.04842 |  | 0.02692 | 0.02074 | 0.03494 |
| 2015 | cardiac | 0.04071 | 0.03346 | 0.04954 |  | 0.02798 | 0.02208 | 0.03544 |
| 2016 | cardiac | 0.04200 | 0.03529 | 0.04997 |  | 0.02924 | 0.02374 | 0.03603 |
| 2017 | cardiac | 0.04279 | 0.03615 | 0.05064 |  | 0.03064 | 0.02496 | 0.03762 |
| 2018 | cardiac | 0.04259 | 0.03518 | 0.05158 |  | 0.03164 | 0.02497 | 0.04010 |
| 2019 | cardiac | 0.04091 | 0.03316 | 0.05046 |  | 0.03156 | 0.02432 | 0.04095 |
| 2020 | cardiac | 0.03742 | 0.03076 | 0.04551 |  | 0.02977 | 0.02338 | 0.03791 |
| 2021 | cardiac | 0.03261 | 0.02694 | 0.03947 |  | 0.02654 | 0.02103 | 0.03348 |
| 2022 | cardiac | 0.02856 | 0.02270 | 0.03593 |  | 0.02413 | 0.01822 | 0.03195 |
| 2023 | cardiac | 0.02685 | 0.02198 | 0.03279 |  | 0.02463 | 0.01925 | 0.03152 |
| 2024 | cardiac | 0.02798 | 0.02140 | 0.03659 |  | 0.02952 | 0.02154 | 0.04046 |
| 2003 | vascular | 0.00571 | 0.00135 | 0.02408 |  | 0.00892 | 0.00146 | 0.05435 |
| 2004 | vascular | 0.00688 | 0.00199 | 0.02377 |  | 0.00953 | 0.00200 | 0.04540 |
| 2005 | vascular | 0.00827 | 0.00290 | 0.02355 |  | 0.01018 | 0.00271 | 0.03818 |
| 2006 | vascular | 0.00988 | 0.00416 | 0.02346 |  | 0.01085 | 0.00363 | 0.03247 |
| 2007 | vascular | 0.01173 | 0.00584 | 0.02356 |  | 0.01156 | 0.00476 | 0.02809 |
| 2008 | vascular | 0.01378 | 0.00793 | 0.02394 |  | 0.01229 | 0.00607 | 0.02487 |
| 2009 | vascular | 0.01599 | 0.01033 | 0.02475 |  | 0.01302 | 0.00746 | 0.02272 |
| 2010 | vascular | 0.01828 | 0.01278 | 0.02615 |  | 0.01376 | 0.00876 | 0.02160 |
| 2011 | vascular | 0.02054 | 0.01494 | 0.02826 |  | 0.01448 | 0.00977 | 0.02144 |
| 2012 | vascular | 0.02264 | 0.01663 | 0.03081 |  | 0.01517 | 0.01046 | 0.02198 |
| 2013 | vascular | 0.02439 | 0.01793 | 0.03318 |  | 0.01581 | 0.01098 | 0.02277 |
| 2014 | vascular | 0.02564 | 0.01897 | 0.03465 |  | 0.01640 | 0.01153 | 0.02333 |
| 2015 | vascular | 0.02622 | 0.01978 | 0.03478 |  | 0.01691 | 0.01223 | 0.02337 |
| 2016 | vascular | 0.02604 | 0.02015 | 0.03366 |  | 0.01731 | 0.01303 | 0.02301 |
| 2017 | vascular | 0.02512 | 0.01950 | 0.03237 |  | 0.01754 | 0.01339 | 0.02299 |
| 2018 | vascular | 0.02385 | 0.01793 | 0.03173 |  | 0.01726 | 0.01271 | 0.02346 |
| 2019 | vascular | 0.02266 | 0.01664 | 0.03086 |  | 0.01614 | 0.01149 | 0.02267 |
| 2020 | vascular | 0.02190 | 0.01649 | 0.02910 |  | 0.01402 | 0.01019 | 0.01930 |
| 2021 | vascular | 0.02157 | 0.01644 | 0.02830 |  | 0.01141 | 0.00838 | 0.01553 |
| 2022 | vascular | 0.02044 | 0.01479 | 0.02827 |  | 0.00989 | 0.00684 | 0.01430 |
| 2023 | vascular | 0.01735 | 0.01314 | 0.02289 |  | 0.01071 | 0.00778 | 0.01473 |
| 2024 | vascular | 0.01279 | 0.00878 | 0.01865 |  | 0.01550 | 0.01030 | 0.02333 |
| 2003 | endocrine_systemic_diseases | 0.01009 | 0.00264 | 0.03861 |  | 0.00577 | 0.00169 | 0.01974 |
| 2004 | endocrine_systemic_diseases | 0.01057 | 0.00333 | 0.03350 |  | 0.00671 | 0.00233 | 0.01933 |
| 2005 | endocrine_systemic_diseases | 0.01107 | 0.00419 | 0.02925 |  | 0.00778 | 0.00318 | 0.01905 |
| 2006 | endocrine_systemic_diseases | 0.01158 | 0.00520 | 0.02580 |  | 0.00898 | 0.00425 | 0.01895 |
| 2007 | endocrine_systemic_diseases | 0.01210 | 0.00634 | 0.02310 |  | 0.01026 | 0.00550 | 0.01912 |
| 2008 | endocrine_systemic_diseases | 0.01263 | 0.00755 | 0.02114 |  | 0.01159 | 0.00684 | 0.01962 |
| 2009 | endocrine_systemic_diseases | 0.01317 | 0.00871 | 0.01990 |  | 0.01289 | 0.00810 | 0.02051 |
| 2010 | endocrine_systemic_diseases | 0.01370 | 0.00967 | 0.01940 |  | 0.01409 | 0.00913 | 0.02175 |
| 2011 | endocrine_systemic_diseases | 0.01422 | 0.01033 | 0.01956 |  | 0.01509 | 0.00987 | 0.02308 |
| 2012 | endocrine_systemic_diseases | 0.01472 | 0.01076 | 0.02013 |  | 0.01578 | 0.01036 | 0.02405 |
| 2013 | endocrine_systemic_diseases | 0.01519 | 0.01112 | 0.02076 |  | 0.01607 | 0.01065 | 0.02425 |
| 2014 | endocrine_systemic_diseases | 0.01564 | 0.01155 | 0.02117 |  | 0.01589 | 0.01080 | 0.02337 |
| 2015 | endocrine_systemic_diseases | 0.01603 | 0.01211 | 0.02122 |  | 0.01520 | 0.01077 | 0.02146 |
| 2016 | endocrine_systemic_diseases | 0.01638 | 0.01270 | 0.02111 |  | 0.01404 | 0.01040 | 0.01894 |
| 2017 | endocrine_systemic_diseases | 0.01668 | 0.01292 | 0.02153 |  | 0.01255 | 0.00939 | 0.01679 |
| 2018 | endocrine_systemic_diseases | 0.01703 | 0.01268 | 0.02287 |  | 0.01121 | 0.00804 | 0.01563 |
| 2019 | endocrine_systemic_diseases | 0.01753 | 0.01268 | 0.02425 |  | 0.01037 | 0.00721 | 0.01491 |
| 2020 | endocrine_systemic_diseases | 0.01834 | 0.01355 | 0.02481 |  | 0.01030 | 0.00737 | 0.01440 |
| 2021 | endocrine_systemic_diseases | 0.01926 | 0.01449 | 0.02560 |  | 0.01117 | 0.00811 | 0.01539 |
| 2022 | endocrine_systemic_diseases | 0.01873 | 0.01349 | 0.02600 |  | 0.01235 | 0.00838 | 0.01820 |
| 2023 | endocrine_systemic_diseases | 0.01527 | 0.01158 | 0.02013 |  | 0.01270 | 0.00905 | 0.01783 |
| 2024 | endocrine_systemic_diseases | 0.01006 | 0.00696 | 0.01455 |  | 0.01159 | 0.00756 | 0.01778 |
| 2003 | dermatological | 0.01460 | 0.00402 | 0.05306 |  | 0.00786 | 0.00149 | 0.04145 |
| 2004 | dermatological | 0.01706 | 0.00564 | 0.05160 |  | 0.00846 | 0.00204 | 0.03505 |
| 2005 | dermatological | 0.01988 | 0.00782 | 0.05051 |  | 0.00909 | 0.00275 | 0.03002 |
| 2006 | dermatological | 0.02302 | 0.01061 | 0.04994 |  | 0.00975 | 0.00362 | 0.02625 |
| 2007 | dermatological | 0.02645 | 0.01397 | 0.05008 |  | 0.01043 | 0.00460 | 0.02364 |
| 2008 | dermatological | 0.03004 | 0.01762 | 0.05119 |  | 0.01112 | 0.00558 | 0.02217 |
| 2009 | dermatological | 0.03364 | 0.02113 | 0.05354 |  | 0.01179 | 0.00638 | 0.02178 |
| 2010 | dermatological | 0.03703 | 0.02402 | 0.05710 |  | 0.01243 | 0.00692 | 0.02233 |
| 2011 | dermatological | 0.03997 | 0.02608 | 0.06126 |  | 0.01302 | 0.00724 | 0.02343 |
| 2012 | dermatological | 0.04218 | 0.02744 | 0.06483 |  | 0.01353 | 0.00745 | 0.02458 |
| 2013 | dermatological | 0.04339 | 0.02832 | 0.06649 |  | 0.01395 | 0.00770 | 0.02526 |
| 2014 | dermatological | 0.04340 | 0.02886 | 0.06526 |  | 0.01423 | 0.00807 | 0.02511 |
| 2015 | dermatological | 0.04207 | 0.02903 | 0.06095 |  | 0.01437 | 0.00859 | 0.02403 |
| 2016 | dermatological | 0.03942 | 0.02853 | 0.05447 |  | 0.01434 | 0.00919 | 0.02238 |
| 2017 | dermatological | 0.03575 | 0.02653 | 0.04819 |  | 0.01420 | 0.00948 | 0.02126 |
| 2018 | dermatological | 0.03193 | 0.02321 | 0.04393 |  | 0.01420 | 0.00928 | 0.02172 |
| 2019 | dermatological | 0.02869 | 0.02044 | 0.04025 |  | 0.01468 | 0.00937 | 0.02301 |
| 2020 | dermatological | 0.02648 | 0.01944 | 0.03608 |  | 0.01606 | 0.01053 | 0.02449 |
| 2021 | dermatological | 0.02535 | 0.01885 | 0.03410 |  | 0.01868 | 0.01181 | 0.02954 |
| 2022 | dermatological | 0.02416 | 0.01687 | 0.03461 |  | 0.02155 | 0.01201 | 0.03867 |
| 2023 | dermatological | 0.02173 | 0.01583 | 0.02984 |  | 0.02262 | 0.01361 | 0.03759 |
| 2024 | dermatological | 0.01798 | 0.01145 | 0.02823 |  | 0.02070 | 0.01198 | 0.03576 |
| 2003 | trauma | 0.00286 | 0.00002 | 0.52870 |  | 0.03143 | 0.00135 | 0.73226 |
| 2004 | trauma | 0.00348 | 0.00004 | 0.34234 |  | 0.02882 | 0.00184 | 0.45244 |
| 2005 | trauma | 0.00423 | 0.00008 | 0.22330 |  | 0.02647 | 0.00248 | 0.28229 |
| 2006 | trauma | 0.00512 | 0.00018 | 0.14771 |  | 0.02441 | 0.00332 | 0.17929 |
| 2007 | trauma | 0.00618 | 0.00038 | 0.09975 |  | 0.02264 | 0.00439 | 0.11689 |
| 2008 | trauma | 0.00742 | 0.00080 | 0.06925 |  | 0.02116 | 0.00567 | 0.07892 |
| 2009 | trauma | 0.00884 | 0.00157 | 0.04979 |  | 0.01996 | 0.00715 | 0.05572 |
| 2010 | trauma | 0.01044 | 0.00292 | 0.03738 |  | 0.01905 | 0.00873 | 0.04159 |
| 2011 | trauma | 0.01221 | 0.00504 | 0.02958 |  | 0.01842 | 0.01022 | 0.03322 |
| 2012 | trauma | 0.01412 | 0.00796 | 0.02505 |  | 0.01809 | 0.01141 | 0.02868 |
| 2013 | trauma | 0.01611 | 0.01115 | 0.02329 |  | 0.01807 | 0.01222 | 0.02672 |
| 2014 | trauma | 0.01813 | 0.01353 | 0.02430 |  | 0.01840 | 0.01291 | 0.02624 |
| 2015 | trauma | 0.02008 | 0.01497 | 0.02692 |  | 0.01913 | 0.01388 | 0.02638 |
| 2016 | trauma | 0.02185 | 0.01649 | 0.02896 |  | 0.02035 | 0.01538 | 0.02694 |
| 2017 | trauma | 0.02336 | 0.01824 | 0.02993 |  | 0.02202 | 0.01680 | 0.02888 |
| 2018 | trauma | 0.02461 | 0.01914 | 0.03165 |  | 0.02338 | 0.01695 | 0.03223 |
| 2019 | trauma | 0.02564 | 0.01939 | 0.03389 |  | 0.02328 | 0.01628 | 0.03331 |
| 2020 | trauma | 0.02650 | 0.02042 | 0.03439 |  | 0.02083 | 0.01513 | 0.02867 |
| 2021 | trauma | 0.02702 | 0.02093 | 0.03489 |  | 0.01665 | 0.01200 | 0.02311 |
| 2022 | trauma | 0.02599 | 0.01847 | 0.03657 |  | 0.01387 | 0.00875 | 0.02199 |
| 2023 | trauma | 0.02231 | 0.01635 | 0.03046 |  | 0.01461 | 0.00935 | 0.02284 |
| 2024 | trauma | 0.01681 | 0.01113 | 0.02539 |  | 0.02127 | 0.01204 | 0.03759 |
| 2003 | genitourinary | 0.00641 | 0.00192 | 0.02143 |  | 0.01421 | 0.00397 | 0.05093 |
| 2004 | genitourinary | 0.00741 | 0.00262 | 0.02095 |  | 0.01453 | 0.00488 | 0.04326 |
| 2005 | genitourinary | 0.00856 | 0.00356 | 0.02058 |  | 0.01486 | 0.00595 | 0.03707 |
| 2006 | genitourinary | 0.00986 | 0.00477 | 0.02037 |  | 0.01519 | 0.00716 | 0.03223 |
| 2007 | genitourinary | 0.01130 | 0.00627 | 0.02038 |  | 0.01553 | 0.00842 | 0.02863 |
| 2008 | genitourinary | 0.01288 | 0.00801 | 0.02070 |  | 0.01588 | 0.00962 | 0.02621 |
| 2009 | genitourinary | 0.01456 | 0.00988 | 0.02147 |  | 0.01624 | 0.01058 | 0.02493 |
| 2010 | genitourinary | 0.01632 | 0.01168 | 0.02281 |  | 0.01662 | 0.01119 | 0.02468 |
| 2011 | genitourinary | 0.01810 | 0.01324 | 0.02473 |  | 0.01700 | 0.01148 | 0.02517 |
| 2012 | genitourinary | 0.01983 | 0.01457 | 0.02699 |  | 0.01740 | 0.01166 | 0.02596 |
| 2013 | genitourinary | 0.02144 | 0.01577 | 0.02914 |  | 0.01781 | 0.01190 | 0.02665 |
| 2014 | genitourinary | 0.02282 | 0.01695 | 0.03074 |  | 0.01823 | 0.01234 | 0.02695 |
| 2015 | genitourinary | 0.02390 | 0.01816 | 0.03147 |  | 0.01868 | 0.01304 | 0.02676 |
| 2016 | genitourinary | 0.02459 | 0.01925 | 0.03140 |  | 0.01914 | 0.01392 | 0.02630 |
| 2017 | genitourinary | 0.02487 | 0.01973 | 0.03135 |  | 0.01961 | 0.01459 | 0.02637 |
| 2018 | genitourinary | 0.02505 | 0.01945 | 0.03227 |  | 0.02012 | 0.01466 | 0.02760 |
| 2019 | genitourinary | 0.02552 | 0.01941 | 0.03356 |  | 0.02065 | 0.01473 | 0.02893 |
| 2020 | genitourinary | 0.02670 | 0.02066 | 0.03451 |  | 0.02121 | 0.01554 | 0.02894 |
| 2021 | genitourinary | 0.02853 | 0.02236 | 0.03642 |  | 0.02180 | 0.01642 | 0.02894 |
| 2022 | genitourinary | 0.02842 | 0.02148 | 0.03761 |  | 0.02236 | 0.01617 | 0.03092 |
| 2023 | genitourinary | 0.02358 | 0.01862 | 0.02986 |  | 0.02280 | 0.01724 | 0.03017 |
| 2024 | genitourinary | 0.01559 | 0.01126 | 0.02157 |  | 0.02310 | 0.01633 | 0.03269 |
| 2003 | gastrointestinal | 0.01333 | 0.00420 | 0.04237 |  | 0.01411 | 0.00455 | 0.04375 |
| 2004 | gastrointestinal | 0.01397 | 0.00517 | 0.03775 |  | 0.01436 | 0.00543 | 0.03796 |
| 2005 | gastrointestinal | 0.01462 | 0.00632 | 0.03383 |  | 0.01460 | 0.00642 | 0.03324 |
| 2006 | gastrointestinal | 0.01528 | 0.00763 | 0.03059 |  | 0.01484 | 0.00746 | 0.02950 |
| 2007 | gastrointestinal | 0.01594 | 0.00906 | 0.02804 |  | 0.01507 | 0.00850 | 0.02671 |
| 2008 | gastrointestinal | 0.01658 | 0.01050 | 0.02619 |  | 0.01527 | 0.00940 | 0.02482 |
| 2009 | gastrointestinal | 0.01719 | 0.01179 | 0.02507 |  | 0.01546 | 0.01006 | 0.02376 |
| 2010 | gastrointestinal | 0.01776 | 0.01278 | 0.02468 |  | 0.01561 | 0.01043 | 0.02337 |
| 2011 | gastrointestinal | 0.01826 | 0.01340 | 0.02488 |  | 0.01573 | 0.01058 | 0.02338 |
| 2012 | gastrointestinal | 0.01867 | 0.01376 | 0.02534 |  | 0.01580 | 0.01065 | 0.02346 |
| 2013 | gastrointestinal | 0.01898 | 0.01403 | 0.02568 |  | 0.01583 | 0.01075 | 0.02331 |
| 2014 | gastrointestinal | 0.01917 | 0.01434 | 0.02561 |  | 0.01580 | 0.01097 | 0.02276 |
| 2015 | gastrointestinal | 0.01921 | 0.01476 | 0.02501 |  | 0.01571 | 0.01132 | 0.02180 |
| 2016 | gastrointestinal | 0.01911 | 0.01519 | 0.02404 |  | 0.01555 | 0.01169 | 0.02069 |
| 2017 | gastrointestinal | 0.01890 | 0.01524 | 0.02343 |  | 0.01531 | 0.01166 | 0.02011 |
| 2018 | gastrointestinal | 0.01881 | 0.01482 | 0.02388 |  | 0.01489 | 0.01097 | 0.02021 |
| 2019 | gastrointestinal | 0.01915 | 0.01476 | 0.02485 |  | 0.01419 | 0.01018 | 0.01978 |
| 2020 | gastrointestinal | 0.02024 | 0.01588 | 0.02582 |  | 0.01315 | 0.00964 | 0.01794 |
| 2021 | gastrointestinal | 0.02216 | 0.01742 | 0.02820 |  | 0.01187 | 0.00871 | 0.01620 |
| 2022 | gastrointestinal | 0.02327 | 0.01736 | 0.03120 |  | 0.01084 | 0.00744 | 0.01581 |
| 2023 | gastrointestinal | 0.02132 | 0.01660 | 0.02739 |  | 0.01050 | 0.00759 | 0.01454 |
| 2024 | gastrointestinal | 0.01637 | 0.01190 | 0.02254 |  | 0.01103 | 0.00744 | 0.01634 |
| 2003 | eye_ear_balance | 0.01689 | 0.00494 | 0.05782 |  | 0.02251 | 0.00489 | 0.10365 |
| 2004 | eye_ear_balance | 0.01828 | 0.00631 | 0.05294 |  | 0.02372 | 0.00631 | 0.08919 |
| 2005 | eye_ear_balance | 0.01977 | 0.00803 | 0.04869 |  | 0.02498 | 0.00809 | 0.07719 |
| 2006 | eye_ear_balance | 0.02138 | 0.01013 | 0.04512 |  | 0.02627 | 0.01023 | 0.06743 |
| 2007 | eye_ear_balance | 0.02309 | 0.01261 | 0.04228 |  | 0.02754 | 0.01271 | 0.05970 |
| 2008 | eye_ear_balance | 0.02492 | 0.01544 | 0.04023 |  | 0.02878 | 0.01539 | 0.05382 |
| 2009 | eye_ear_balance | 0.02685 | 0.01845 | 0.03908 |  | 0.02994 | 0.01805 | 0.04968 |
| 2010 | eye_ear_balance | 0.02889 | 0.02140 | 0.03900 |  | 0.03099 | 0.02036 | 0.04718 |
| 2011 | eye_ear_balance | 0.03102 | 0.02400 | 0.04010 |  | 0.03188 | 0.02205 | 0.04610 |
| 2012 | eye_ear_balance | 0.03324 | 0.02614 | 0.04227 |  | 0.03258 | 0.02310 | 0.04593 |
| 2013 | eye_ear_balance | 0.03553 | 0.02805 | 0.04499 |  | 0.03302 | 0.02376 | 0.04590 |
| 2014 | eye_ear_balance | 0.03787 | 0.03009 | 0.04765 |  | 0.03319 | 0.02433 | 0.04528 |
| 2015 | eye_ear_balance | 0.04024 | 0.03250 | 0.04982 |  | 0.03304 | 0.02499 | 0.04369 |
| 2016 | eye_ear_balance | 0.04262 | 0.03522 | 0.05159 |  | 0.03255 | 0.02559 | 0.04141 |
| 2017 | eye_ear_balance | 0.04492 | 0.03734 | 0.05403 |  | 0.03179 | 0.02537 | 0.03984 |
| 2018 | eye_ear_balance | 0.04673 | 0.03791 | 0.05761 |  | 0.03113 | 0.02408 | 0.04024 |
| 2019 | eye_ear_balance | 0.04755 | 0.03777 | 0.05985 |  | 0.03099 | 0.02328 | 0.04125 |
| 2020 | eye_ear_balance | 0.04687 | 0.03783 | 0.05806 |  | 0.03180 | 0.02431 | 0.04159 |
| 2021 | eye_ear_balance | 0.04441 | 0.03628 | 0.05437 |  | 0.03376 | 0.02605 | 0.04375 |
| 2022 | eye_ear_balance | 0.04037 | 0.03177 | 0.05130 |  | 0.03574 | 0.02615 | 0.04884 |
| 2023 | eye_ear_balance | 0.03517 | 0.02857 | 0.04330 |  | 0.03599 | 0.02733 | 0.04740 |
| 2024 | eye_ear_balance | 0.02957 | 0.02240 | 0.03904 |  | 0.03370 | 0.02323 | 0.04889 |
| 2003 | psychiatric | 0.00829 | 0.00238 | 0.02885 |  | 0.00315 | 0.00050 | 0.01980 |
| 2004 | psychiatric | 0.00907 | 0.00309 | 0.02662 |  | 0.00423 | 0.00086 | 0.02072 |
| 2005 | psychiatric | 0.00990 | 0.00398 | 0.02467 |  | 0.00566 | 0.00147 | 0.02176 |
| 2006 | psychiatric | 0.01080 | 0.00506 | 0.02302 |  | 0.00749 | 0.00245 | 0.02294 |
| 2007 | psychiatric | 0.01173 | 0.00634 | 0.02169 |  | 0.00977 | 0.00393 | 0.02431 |
| 2008 | psychiatric | 0.01269 | 0.00776 | 0.02073 |  | 0.01249 | 0.00600 | 0.02598 |
| 2009 | psychiatric | 0.01365 | 0.00922 | 0.02020 |  | 0.01556 | 0.00862 | 0.02810 |
| 2010 | psychiatric | 0.01458 | 0.01055 | 0.02014 |  | 0.01882 | 0.01149 | 0.03083 |
| 2011 | psychiatric | 0.01546 | 0.01162 | 0.02058 |  | 0.02197 | 0.01413 | 0.03414 |
| 2012 | psychiatric | 0.01625 | 0.01238 | 0.02132 |  | 0.02463 | 0.01618 | 0.03750 |
| 2013 | psychiatric | 0.01690 | 0.01295 | 0.02207 |  | 0.02640 | 0.01753 | 0.03976 |
| 2014 | psychiatric | 0.01740 | 0.01346 | 0.02248 |  | 0.02691 | 0.01821 | 0.03976 |
| 2015 | psychiatric | 0.01768 | 0.01398 | 0.02237 |  | 0.02595 | 0.01819 | 0.03701 |
| 2016 | psychiatric | 0.01774 | 0.01441 | 0.02184 |  | 0.02356 | 0.01725 | 0.03217 |
| 2017 | psychiatric | 0.01756 | 0.01435 | 0.02148 |  | 0.02014 | 0.01489 | 0.02724 |
| 2018 | psychiatric | 0.01723 | 0.01369 | 0.02169 |  | 0.01658 | 0.01171 | 0.02347 |
| 2019 | psychiatric | 0.01685 | 0.01307 | 0.02172 |  | 0.01349 | 0.00917 | 0.01986 |
| 2020 | psychiatric | 0.01652 | 0.01302 | 0.02095 |  | 0.01116 | 0.00779 | 0.01599 |
| 2021 | psychiatric | 0.01624 | 0.01288 | 0.02046 |  | 0.00968 | 0.00695 | 0.01348 |
| 2022 | psychiatric | 0.01570 | 0.01189 | 0.02074 |  | 0.00919 | 0.00622 | 0.01358 |
| 2023 | psychiatric | 0.01459 | 0.01147 | 0.01856 |  | 0.01002 | 0.00714 | 0.01406 |
| 2024 | psychiatric | 0.01290 | 0.00956 | 0.01740 |  | 0.01266 | 0.00861 | 0.01859 |
| 2003 | dementia_memory_disorders | 0.00726 | 0.00150 | 0.03513 |  | 0.07957 | 0.00089 | 7.07756 |
| 2004 | dementia_memory_disorders | 0.00839 | 0.00215 | 0.03271 |  | 0.06841 | 0.00139 | 3.36099 |
| 2005 | dementia_memory_disorders | 0.00967 | 0.00305 | 0.03064 |  | 0.05889 | 0.00215 | 1.61495 |
| 2006 | dementia_memory_disorders | 0.01112 | 0.00427 | 0.02897 |  | 0.05082 | 0.00326 | 0.79333 |
| 2007 | dementia_memory_disorders | 0.01274 | 0.00584 | 0.02776 |  | 0.04402 | 0.00481 | 0.40276 |
| 2008 | dementia_memory_disorders | 0.01450 | 0.00775 | 0.02710 |  | 0.03831 | 0.00687 | 0.21381 |
| 2009 | dementia_memory_disorders | 0.01638 | 0.00989 | 0.02713 |  | 0.03356 | 0.00936 | 0.12031 |
| 2010 | dementia_memory_disorders | 0.01834 | 0.01202 | 0.02798 |  | 0.02961 | 0.01200 | 0.07306 |
| 2011 | dementia_memory_disorders | 0.02032 | 0.01390 | 0.02970 |  | 0.02635 | 0.01410 | 0.04923 |
| 2012 | dementia_memory_disorders | 0.02225 | 0.01547 | 0.03201 |  | 0.02368 | 0.01468 | 0.03821 |
| 2013 | dementia_memory_disorders | 0.02404 | 0.01682 | 0.03436 |  | 0.02153 | 0.01360 | 0.03407 |
| 2014 | dementia_memory_disorders | 0.02559 | 0.01812 | 0.03612 |  | 0.01981 | 0.01218 | 0.03222 |
| 2015 | dementia_memory_disorders | 0.02679 | 0.01942 | 0.03695 |  | 0.01847 | 0.01132 | 0.03016 |
| 2016 | dementia_memory_disorders | 0.02756 | 0.02044 | 0.03714 |  | 0.01749 | 0.01114 | 0.02745 |
| 2017 | dementia_memory_disorders | 0.02775 | 0.02037 | 0.03782 |  | 0.01680 | 0.01108 | 0.02547 |
| 2018 | dementia_memory_disorders | 0.02711 | 0.01896 | 0.03877 |  | 0.01626 | 0.01029 | 0.02570 |
| 2019 | dementia_memory_disorders | 0.02541 | 0.01728 | 0.03737 |  | 0.01575 | 0.00942 | 0.02635 |
| 2020 | dementia_memory_disorders | 0.02260 | 0.01597 | 0.03197 |  | 0.01514 | 0.00931 | 0.02463 |
| 2021 | dementia_memory_disorders | 0.01907 | 0.01393 | 0.02610 |  | 0.01436 | 0.00941 | 0.02190 |
| 2022 | dementia_memory_disorders | 0.01599 | 0.01097 | 0.02329 |  | 0.01348 | 0.00841 | 0.02161 |
| 2023 | dementia_memory_disorders | 0.01410 | 0.01019 | 0.01949 |  | 0.01260 | 0.00838 | 0.01894 |
| 2024 | dementia_memory_disorders | 0.01347 | 0.00946 | 0.01919 |  | 0.01178 | 0.00751 | 0.01849 |
| 2003 | symptoms_and_complaints | 0.01460 | 0.00330 | 0.06461 |  | 0.00359 | 0.00031 | 0.04214 |
| 2004 | symptoms_and_complaints | 0.01507 | 0.00424 | 0.05360 |  | 0.00482 | 0.00059 | 0.03961 |
| 2005 | symptoms_and_complaints | 0.01556 | 0.00540 | 0.04485 |  | 0.00645 | 0.00110 | 0.03761 |
| 2006 | symptoms_and_complaints | 0.01606 | 0.00678 | 0.03806 |  | 0.00854 | 0.00201 | 0.03628 |
| 2007 | symptoms_and_complaints | 0.01657 | 0.00833 | 0.03299 |  | 0.01115 | 0.00347 | 0.03580 |
| 2008 | symptoms_and_complaints | 0.01710 | 0.00992 | 0.02949 |  | 0.01429 | 0.00560 | 0.03650 |
| 2009 | symptoms_and_complaints | 0.01764 | 0.01134 | 0.02746 |  | 0.01788 | 0.00823 | 0.03885 |
| 2010 | symptoms_and_complaints | 0.01820 | 0.01234 | 0.02682 |  | 0.02175 | 0.01094 | 0.04323 |
| 2011 | symptoms_and_complaints | 0.01877 | 0.01290 | 0.02731 |  | 0.02558 | 0.01325 | 0.04936 |
| 2012 | symptoms_and_complaints | 0.01935 | 0.01319 | 0.02837 |  | 0.02896 | 0.01505 | 0.05570 |
| 2013 | symptoms_and_complaints | 0.01994 | 0.01350 | 0.02945 |  | 0.03141 | 0.01647 | 0.05990 |
| 2014 | symptoms_and_complaints | 0.02054 | 0.01397 | 0.03022 |  | 0.03248 | 0.01761 | 0.05993 |
| 2015 | symptoms_and_complaints | 0.02116 | 0.01459 | 0.03067 |  | 0.03188 | 0.01829 | 0.05557 |
| 2016 | symptoms_and_complaints | 0.02178 | 0.01510 | 0.03141 |  | 0.02955 | 0.01770 | 0.04933 |
| 2017 | symptoms_and_complaints | 0.02241 | 0.01491 | 0.03369 |  | 0.02589 | 0.01468 | 0.04568 |
| 2018 | symptoms_and_complaints | 0.02305 | 0.01419 | 0.03743 |  | 0.02196 | 0.01078 | 0.04476 |
| 2019 | symptoms_and_complaints | 0.02369 | 0.01394 | 0.04024 |  | 0.01856 | 0.00823 | 0.04186 |
| 2020 | symptoms_and_complaints | 0.02432 | 0.01507 | 0.03924 |  | 0.01611 | 0.00759 | 0.03418 |
| 2021 | symptoms_and_complaints | 0.02479 | 0.01763 | 0.03485 |  | 0.01474 | 0.00868 | 0.02502 |
| 2022 | symptoms_and_complaints | 0.02431 | 0.01840 | 0.03211 |  | 0.01454 | 0.00976 | 0.02166 |
| 2023 | symptoms_and_complaints | 0.02209 | 0.01747 | 0.02793 |  | 0.01576 | 0.01132 | 0.02196 |
| 2024 | symptoms_and_complaints | 0.01837 | 0.01377 | 0.02451 |  | 0.01873 | 0.01289 | 0.02723 |
| 2003 | congenital | 0.01426 | 0.00305 | 0.06670 |  | 0.00147 | 0.00010 | 0.02255 |
| 2004 | congenital | 0.01559 | 0.00374 | 0.06497 |  | 0.00152 | 0.00011 | 0.02163 |
| 2005 | congenital | 0.01587 | 0.00437 | 0.05765 |  | 0.00157 | 0.00012 | 0.02099 |
| 2006 | congenital | 0.01397 | 0.00456 | 0.04283 |  | 0.00163 | 0.00013 | 0.02062 |
| 2007 | congenital | 0.00990 | 0.00357 | 0.02745 |  | 0.00168 | 0.00014 | 0.02050 |
| 2008 | congenital | 0.00525 | 0.00139 | 0.01991 |  | 0.00174 | 0.00015 | 0.02060 |
| 2009 | congenital | 0.00194 | 0.00020 | 0.01873 |  | 0.00180 | 0.00015 | 0.02089 |
| 2010 | congenital | 0.00046 | 0.00001 | 0.02023 |  | 0.00185 | 0.00016 | 0.02130 |
| 2011 | congenital | 0.00007 | 0.00000 | 0.02332 |  | 0.00190 | 0.00017 | 0.02180 |
| 2012 | congenital | 0.00001 | 0.00000 | 0.02803 |  | 0.00195 | 0.00017 | 0.02231 |
| 2013 | congenital | 0.00000 | 0.00000 | 0.03487 |  | 0.00199 | 0.00017 | 0.02279 |
| 2014 | congenital | 0.00000 | 0.00000 | 0.04485 |  | 0.00203 | 0.00018 | 0.02320 |
| 2015 | congenital | 0.00000 | 0.00000 | 0.05972 |  | 0.00207 | 0.00018 | 0.02350 |
| 2016 | congenital | 0.00000 | 0.00000 | 0.08246 |  | 0.00210 | 0.00019 | 0.02373 |
| 2017 | congenital | 0.00000 | 0.00000 | 0.11609 |  | 0.00211 | 0.00019 | 0.02390 |
| 2018 | congenital | 0.00000 | 0.00000 | 0.15130 |  | 0.00210 | 0.00019 | 0.02386 |
| 2019 | congenital | 0.00000 | 0.00000 | 0.16250 |  | 0.00204 | 0.00018 | 0.02328 |
| 2020 | congenital | 0.00000 | 0.00000 | 0.12808 |  | 0.00192 | 0.00017 | 0.02186 |
| 2021 | congenital | 0.00000 | 0.00000 | 0.07082 |  | 0.00176 | 0.00015 | 0.02000 |
| 2022 | congenital | 0.00000 | 0.00000 | 0.03486 |  | 0.00167 | 0.00015 | 0.01913 |
| 2023 | congenital | 0.00637 | 0.00159 | 0.02552 |  | 0.00180 | 0.00016 | 0.02040 |
| 2024 | congenital | 0.02784 | 0.00974 | 0.07959 |  | 0.00225 | 0.00020 | 0.02574 |
| 2003 | neoplasms | 0.00951 | 0.00417 | 0.02168 |  | 0.01346 | 0.00602 | 0.03006 |
| 2004 | neoplasms | 0.01009 | 0.00497 | 0.02048 |  | 0.01355 | 0.00680 | 0.02701 |
| 2005 | neoplasms | 0.01071 | 0.00590 | 0.01945 |  | 0.01365 | 0.00763 | 0.02442 |
| 2006 | neoplasms | 0.01137 | 0.00694 | 0.01862 |  | 0.01376 | 0.00849 | 0.02229 |
| 2007 | neoplasms | 0.01207 | 0.00808 | 0.01803 |  | 0.01388 | 0.00933 | 0.02065 |
| 2008 | neoplasms | 0.01281 | 0.00926 | 0.01774 |  | 0.01402 | 0.01008 | 0.01950 |
| 2009 | neoplasms | 0.01361 | 0.01039 | 0.01782 |  | 0.01418 | 0.01066 | 0.01886 |
| 2010 | neoplasms | 0.01446 | 0.01141 | 0.01833 |  | 0.01437 | 0.01105 | 0.01868 |
| 2011 | neoplasms | 0.01537 | 0.01228 | 0.01923 |  | 0.01459 | 0.01130 | 0.01884 |
| 2012 | neoplasms | 0.01634 | 0.01308 | 0.02041 |  | 0.01485 | 0.01151 | 0.01916 |
| 2013 | neoplasms | 0.01738 | 0.01395 | 0.02166 |  | 0.01516 | 0.01180 | 0.01947 |
| 2014 | neoplasms | 0.01850 | 0.01499 | 0.02284 |  | 0.01551 | 0.01224 | 0.01967 |
| 2015 | neoplasms | 0.01971 | 0.01627 | 0.02387 |  | 0.01593 | 0.01287 | 0.01972 |
| 2016 | neoplasms | 0.02100 | 0.01774 | 0.02486 |  | 0.01642 | 0.01361 | 0.01980 |
| 2017 | neoplasms | 0.02235 | 0.01899 | 0.02630 |  | 0.01697 | 0.01414 | 0.02038 |
| 2018 | neoplasms | 0.02347 | 0.01950 | 0.02824 |  | 0.01756 | 0.01424 | 0.02165 |
| 2019 | neoplasms | 0.02397 | 0.01957 | 0.02937 |  | 0.01812 | 0.01441 | 0.02279 |
| 2020 | neoplasms | 0.02350 | 0.01945 | 0.02840 |  | 0.01858 | 0.01502 | 0.02300 |
| 2021 | neoplasms | 0.02193 | 0.01826 | 0.02634 |  | 0.01886 | 0.01531 | 0.02323 |
| 2022 | neoplasms | 0.01978 | 0.01590 | 0.02460 |  | 0.01876 | 0.01459 | 0.02413 |
| 2023 | neoplasms | 0.01761 | 0.01456 | 0.02129 |  | 0.01810 | 0.01451 | 0.02257 |
| 2024 | neoplasms | 0.01573 | 0.01208 | 0.02049 |  | 0.01689 | 0.01262 | 0.02262 |

# Appendix 6. Negative binomial regression of Clusters

## Model 1: Negative binomial regression of year, by sex

| model | term | estimate | conf.low | conf.high | p.value |
| --- | --- | --- | --- | --- | --- |
| M1 female | (Intercept) | -275.525 | -316.365 | -234.678 | 0.000 |
| M1 female | YEAR | 0.132 | 0.112 | 0.152 | 0.000 |
| M1 male | (Intercept) | -291.280 | -331.779 | -250.652 | 0.000 |
| M1 male | YEAR | 0.140 | 0.120 | 0.160 | 0.000 |

## Model 2: Negative binomial regression of year x cluster by sex

| model | term | estimate | conf.low | conf.high | p.value |
| --- | --- | --- | --- | --- | --- |
| M2 female | (Intercept) | -248.616 | -333.338 | -163.962 | 0.000 |
| M2 female | YEAR | 0.118 | 0.076 | 0.160 | 0.000 |
| M2 female | cluster2 | -0.778 | -121.253 | 119.822 | 0.989 |
| M2 female | cluster3 | 14.430 | -109.349 | 138.714 | 0.800 |
| M2 female | cluster4 | -58.271 | -185.621 | 68.858 | 0.305 |
| M2 female | cluster5 | -3.284 | -124.423 | 118.141 | 0.953 |
| M2 female | YEAR#cluster2 | 0.001 | -0.059 | 0.060 | 0.979 |
| M2 female | YEAR#cluster3 | -0.007 | -0.069 | 0.054 | 0.802 |
| M2 female | YEAR#cluster4 | 0.029 | -0.034 | 0.092 | 0.298 |
| M2 female | YEAR#cluster5 | 0.002 | -0.058 | 0.062 | 0.946 |
| M2 male | (Intercept) | -297.757 | -376.474 | -218.510 | 0.000 |
| M2 male | YEAR | 0.143 | 0.104 | 0.182 | 0.000 |
| M2 male | cluster2 | 16.477 | -103.816 | 136.933 | 0.760 |
| M2 male | cluster3 | 31.827 | -86.680 | 149.991 | 0.552 |
| M2 male | cluster4 | -21.006 | -153.152 | 111.556 | 0.708 |
| M2 male | cluster5 | 38.103 | -76.737 | 153.336 | 0.465 |
| M2 male | YEAR#cluster2 | -0.008 | -0.068 | 0.051 | 0.758 |
| M2 male | YEAR#cluster3 | -0.015 | -0.074 | 0.043 | 0.559 |
| M2 male | YEAR#cluster4 | 0.011 | -0.055 | 0.076 | 0.705 |
| M2 male | YEAR#cluster5 | -0.019 | -0.076 | 0.038 | 0.462 |

## Model 3: Negative binomial regression of year x cluster x language, by sex

| model | term | estimate | conf.low | conf.high | p.value |
| --- | --- | --- | --- | --- | --- |
| M3 female | (Intercept) | -286.289 | -424.020 | -148.320 | 0.000 |
| M3 female | YEAR | 0.137 | 0.069 | 0.205 | 0.000 |
| M3 female | cluster2 | -89.313 | -301.619 | 121.910 | 0.374 |
| M3 female | cluster3 | 11.868 | -178.265 | 203.158 | 0.899 |
| M3 female | cluster4 | -91.502 | -305.004 | 121.778 | 0.367 |
| M3 female | cluster5 | 20.723 | -180.775 | 222.677 | 0.834 |
| M3 female | REGION:NL | 36.426 | -139.132 | 211.372 | 0.659 |
| M3 female | YEAR#cluster2 | 0.045 | -0.060 | 0.150 | 0.371 |
| M3 female | YEAR#cluster3 | -0.006 | -0.101 | 0.088 | 0.900 |
| M3 female | YEAR#cluster4 | 0.046 | -0.060 | 0.152 | 0.363 |
| M3 female | YEAR#cluster5 | -0.010 | -0.110 | 0.090 | 0.838 |
| M3 female | YEAR#REGION:NL | -0.018 | -0.105 | 0.069 | 0.663 |
| M3 female | cluster2#REGION:NL | 94.993 | -165.360 | 356.142 | 0.430 |
| M3 female | cluster3#REGION:NL | 18.021 | -232.630 | 268.292 | 0.879 |
| M3 female | cluster4#REGION:NL | 33.218 | -233.740 | 300.142 | 0.787 |
| M3 female | cluster5#REGION:NL | -43.481 | -297.423 | 210.335 | 0.717 |
| M3 female | YEAR#cluster2#REGION:NL | -0.047 | -0.176 | 0.082 | 0.431 |
| M3 female | YEAR#cluster3#REGION:NL | -0.009 | -0.133 | 0.115 | 0.879 |
| M3 female | YEAR#cluster4#REGION:NL | -0.016 | -0.149 | 0.116 | 0.787 |
| M3 female | YEAR#cluster5#REGION:NL | 0.022 | -0.104 | 0.147 | 0.717 |
| M3 male | (Intercept) | -326.060 | -468.003 | -184.424 | 0.000 |
| M3 male | YEAR | 0.157 | 0.087 | 0.227 | 0.000 |
| M3 male | cluster2 | 109.237 | -94.993 | 314.730 | 0.250 |
| M3 male | cluster3 | 73.532 | -146.822 | 297.176 | 0.451 |
| M3 male | cluster4 | 61.291 | -184.626 | 312.382 | 0.581 |
| M3 male | cluster5 | 88.672 | -104.122 | 283.545 | 0.326 |
| M3 male | REGION:NL | 22.188 | -149.449 | 194.114 | 0.783 |
| M3 male | YEAR#cluster2 | -0.054 | -0.156 | 0.047 | 0.249 |
| M3 male | YEAR#cluster3 | -0.036 | -0.147 | 0.073 | 0.455 |
| M3 male | YEAR#cluster4 | -0.030 | -0.155 | 0.092 | 0.581 |
| M3 male | YEAR#cluster5 | -0.044 | -0.141 | 0.052 | 0.326 |
| M3 male | YEAR#REGION:NL | -0.011 | -0.096 | 0.074 | 0.787 |
| M3 male | cluster2#REGION:NL | -132.078 | -386.589 | 121.660 | 0.254 |
| M3 male | cluster3#REGION:NL | -50.118 | -315.064 | 212.212 | 0.669 |
| M3 male | cluster4#REGION:NL | -125.919 | -423.259 | 167.659 | 0.331 |
| M3 male | cluster5#REGION:NL | -61.000 | -303.260 | 180.159 | 0.583 |
| M3 male | YEAR#cluster2#REGION:NL | 0.065 | -0.060 | 0.192 | 0.254 |
| M3 male | YEAR#cluster3#REGION:NL | 0.025 | -0.105 | 0.156 | 0.670 |
| M3 male | YEAR#cluster4#REGION:NL | 0.063 | -0.083 | 0.210 | 0.330 |
| M3 male | YEAR#cluster5#REGION:NL | 0.030 | -0.089 | 0.150 | 0.584 |

## Model 4: Negative binomial regression of year x cluster x age groups, by sex

| model | term | estimate | conf.low | conf.high | p.value |
| --- | --- | --- | --- | --- | --- |
| M4 female | (Intercept) | -210.452 | -319.755 | -108.751 | 0.000 |
| M4 female | YEAR | 0.099 | 0.048 | 0.153 | 0.000 |
| M4 female | cluster2 | -4.460 | -144.774 | 137.974 | 0.951 |
| M4 female | cluster3 | 72.550 | -73.105 | 218.954 | 0.335 |
| M4 female | cluster4 | 72.042 | -78.124 | 222.791 | 0.353 |
| M4 female | cluster5 | 102.493 | -49.961 | 254.823 | 0.189 |
| M4 female | AGEGROUP70-79 | -31.919 | -155.170 | 95.730 | 0.623 |
| M4 female | AGEGROUP80-89 | -79.973 | -200.273 | 45.374 | 0.205 |
| M4 female | YEAR#cluster2 | 0.002 | -0.068 | 0.072 | 0.948 |
| M4 female | YEAR#cluster3 | -0.036 | -0.108 | 0.036 | 0.335 |
| M4 female | YEAR#cluster4 | -0.036 | -0.111 | 0.039 | 0.351 |
| M4 female | YEAR#cluster5 | -0.051 | -0.126 | 0.025 | 0.188 |
| M4 female | YEAR#AGEGROUP70-79 | 0.016 | -0.047 | 0.077 | 0.613 |
| M4 female | YEAR#AGEGROUP80-89 | 0.041 | -0.021 | 0.100 | 0.193 |
| M4 female | cluster2#AGEGROUP70-79 | 32.217 | -136.933 | 200.142 | 0.712 |
| M4 female | cluster3#AGEGROUP70-79 | -69.183 | -245.239 | 106.317 | 0.446 |
| M4 female | cluster4#AGEGROUP70-79 | -38.928 | -219.460 | 141.135 | 0.676 |
| M4 female | cluster5#AGEGROUP70-79 | -67.143 | -250.050 | 115.688 | 0.475 |
| M4 female | cluster2#AGEGROUP80-89 | 37.467 | -127.589 | 201.116 | 0.657 |
| M4 female | cluster3#AGEGROUP80-89 | 4.938 | -165.002 | 174.424 | 0.955 |
| M4 female | cluster4#AGEGROUP80-89 | -89.164 | -263.224 | 84.508 | 0.317 |
| M4 female | cluster5#AGEGROUP80-89 | -82.230 | -255.854 | 91.595 | 0.354 |
| M4 female | YEAR#cluster2#AGEGROUP70-79 | -0.016 | -0.099 | 0.068 | 0.712 |
| M4 female | YEAR#cluster3#AGEGROUP70-79 | 0.034 | -0.053 | 0.122 | 0.446 |
| M4 female | YEAR#cluster4#AGEGROUP70-79 | 0.019 | -0.070 | 0.109 | 0.674 |
| M4 female | YEAR#cluster5#AGEGROUP70-79 | 0.033 | -0.057 | 0.124 | 0.474 |
| M4 female | YEAR#cluster2#AGEGROUP80-89 | -0.018 | -0.100 | 0.063 | 0.659 |
| M4 female | YEAR#cluster3#AGEGROUP80-89 | -0.002 | -0.086 | 0.082 | 0.955 |
| M4 female | YEAR#cluster4#AGEGROUP80-89 | 0.045 | -0.042 | 0.131 | 0.313 |
| M4 female | YEAR#cluster5#AGEGROUP80-89 | 0.041 | -0.045 | 0.127 | 0.351 |
| M4 male | (Intercept) | -138.435 | -213.115 | -67.520 | 0.000 |
| M4 male | YEAR | 0.063 | 0.028 | 0.100 | 0.001 |
| M4 male | cluster2 | 64.695 | -41.678 | 171.459 | 0.234 |
| M4 male | cluster3 | 90.796 | -14.610 | 196.353 | 0.094 |
| M4 male | cluster4 | -75.916 | -221.137 | 59.813 | 0.290 |
| M4 male | cluster5 | -46.123 | -151.963 | 58.757 | 0.394 |
| M4 male | AGEGROUP70-79 | -106.758 | -198.087 | -13.943 | 0.023 |
| M4 male | AGEGROUP80-89 | -120.119 | -208.839 | -29.557 | 0.009 |
| M4 male | YEAR#cluster2 | -0.032 | -0.085 | 0.021 | 0.232 |
| M4 male | YEAR#cluster3 | -0.045 | -0.098 | 0.007 | 0.093 |
| M4 male | YEAR#cluster4 | 0.037 | -0.030 | 0.109 | 0.294 |
| M4 male | YEAR#cluster5 | 0.023 | -0.029 | 0.075 | 0.395 |
| M4 male | YEAR#AGEGROUP70-79 | 0.054 | 0.008 | 0.099 | 0.022 |
| M4 male | YEAR#AGEGROUP80-89 | 0.061 | 0.016 | 0.105 | 0.007 |
| M4 male | cluster2#AGEGROUP70-79 | -42.628 | -180.069 | 94.153 | 0.543 |
| M4 male | cluster3#AGEGROUP70-79 | 0.394 | -134.086 | 134.719 | 0.995 |
| M4 male | cluster4#AGEGROUP70-79 | 138.442 | -30.447 | 312.685 | 0.114 |
| M4 male | cluster5#AGEGROUP70-79 | 73.893 | -59.257 | 207.502 | 0.280 |
| M4 male | cluster2#AGEGROUP80-89 | -73.753 | -209.566 | 61.380 | 0.285 |
| M4 male | cluster3#AGEGROUP80-89 | -75.258 | -203.822 | 53.239 | 0.254 |
| M4 male | cluster4#AGEGROUP80-89 | 102.324 | -56.965 | 268.517 | 0.216 |
| M4 male | cluster5#AGEGROUP80-89 | 51.770 | -80.785 | 184.748 | 0.446 |
| M4 male | YEAR#cluster2#AGEGROUP70-79 | 0.021 | -0.047 | 0.089 | 0.545 |
| M4 male | YEAR#cluster3#AGEGROUP70-79 | 0.000 | -0.067 | 0.067 | 0.997 |
| M4 male | YEAR#cluster4#AGEGROUP70-79 | -0.069 | -0.155 | 0.015 | 0.113 |
| M4 male | YEAR#cluster5#AGEGROUP70-79 | -0.037 | -0.103 | 0.029 | 0.279 |
| M4 male | YEAR#cluster2#AGEGROUP80-89 | 0.036 | -0.031 | 0.104 | 0.286 |
| M4 male | YEAR#cluster3#AGEGROUP80-89 | 0.038 | -0.026 | 0.101 | 0.252 |
| M4 male | YEAR#cluster4#AGEGROUP80-89 | -0.051 | -0.133 | 0.028 | 0.217 |
| M4 male | YEAR#cluster5#AGEGROUP80-89 | -0.026 | -0.092 | 0.040 | 0.445 |

# Appendix 7. Negative binomial regression of Clusters with splines

## Model 1: Negative binomial regression of year, by sex

### Predictions

| YEAR | Predicted rate per 1000 | Lower 95%CI | Upper 95%CI | sex |
| --- | --- | --- | --- | --- |
| 2003 | 0.0075915 | 0.00445987 | 0.01292211 | female |
| 2004 | 0.00957524 | 0.00626569 | 0.0146329 | female |
| 2005 | 0.01206552 | 0.00867087 | 0.01678919 | female |
| 2006 | 0.0151737 | 0.01168754 | 0.01969971 | female |
| 2007 | 0.01902658 | 0.01519067 | 0.02383112 | female |
| 2008 | 0.02376446 | 0.01907409 | 0.02960819 | female |
| 2009 | 0.02953709 | 0.02352567 | 0.03708459 | female |
| 2010 | 0.0364968 | 0.02899909 | 0.04593304 | female |
| 2011 | 0.0447882 | 0.03605431 | 0.05563781 | female |
| 2012 | 0.0544986 | 0.04499952 | 0.06600288 | female |
| 2013 | 0.06547548 | 0.05492201 | 0.07805683 | female |
| 2014 | 0.07728919 | 0.06429618 | 0.09290782 | female |
| 2015 | 0.08920329 | 0.07287401 | 0.10919156 | female |
| 2016 | 0.10017061 | 0.08161596 | 0.12294349 | female |
| 2017 | 0.10919897 | 0.09089015 | 0.13119591 | female |
| 2018 | 0.11652575 | 0.09850753 | 0.13783971 | female |
| 2019 | 0.12305601 | 0.10244949 | 0.14780729 | female |
| 2020 | 0.1300211 | 0.10548733 | 0.16026082 | female |
| 2021 | 0.13896563 | 0.11258017 | 0.17153506 | female |
| 2022 | 0.15142693 | 0.12591207 | 0.18211213 | female |
| 2023 | 0.16749001 | 0.1365831 | 0.20539074 | female |
| 2024 | 0.18664644 | 0.13699759 | 0.25428835 | female |
| 2003 | 0.00909009 | 0.00541684 | 0.01525421 | male |
| 2004 | 0.01006529 | 0.00668711 | 0.01515007 | male |
| 2005 | 0.01119996 | 0.00817144 | 0.01535093 | male |
| 2006 | 0.01258549 | 0.00981813 | 0.01613285 | male |
| 2007 | 0.0143522 | 0.01153953 | 0.01785043 | male |
| 2008 | 0.01669143 | 0.01339836 | 0.02079388 | male |
| 2009 | 0.01989422 | 0.01576455 | 0.0251057 | male |
| 2010 | 0.02442027 | 0.01924853 | 0.03098156 | male |
| 2011 | 0.03102383 | 0.02474868 | 0.03889008 | male |
| 2012 | 0.04076596 | 0.03337396 | 0.0497952 | male |
| 2013 | 0.05416363 | 0.04512795 | 0.06500846 | male |
| 2014 | 0.07074249 | 0.05857216 | 0.08544163 | male |
| 2015 | 0.08830191 | 0.07186894 | 0.10849233 | male |
| 2016 | 0.10240746 | 0.08315623 | 0.12611549 | male |
| 2017 | 0.10850065 | 0.09008508 | 0.13068081 | male |
| 2018 | 0.10804171 | 0.09133257 | 0.12780777 | male |
| 2019 | 0.10520612 | 0.0878226 | 0.12603052 | male |
| 2020 | 0.10423487 | 0.08488605 | 0.12799404 | male |
| 2021 | 0.10933006 | 0.08890065 | 0.13445415 | male |
| 2022 | 0.12480963 | 0.10418534 | 0.14951664 | male |
| 2023 | 0.15196642 | 0.12466319 | 0.18524949 | male |
| 2024 | 0.19109209 | 0.14149428 | 0.25807535 | male |

### Model estimates

| Model | Term | IRR, 95%CI | Estimate, 95%CI | p-value |
| --- | --- | --- | --- | --- |
| Female | (Intercept) | 0.000 (0.000-0.000) | -11.788 (-12.320--11.235) | <0.001 |
| Female | Year, knot number 1 | 15.096 (8.182-27.610) | 2.714 (2.102-3.318) | <0.001 |
| Female | Year, knot number 2 | 11.310 (6.340-20.134) | 2.426 (1.847-3.002) | <0.001 |
| Female | Year, knot number 3 | 87.094 (24.083-304.957) | 4.467 (3.182-5.720) | <0.001 |
| Female | Year, knot number 4 | 9.049 (5.881-14.092) | 2.203 (1.772-2.646) | <0.001 |
| Male | (Intercept) | 0.000 (0.000-0.000) | -11.608 (-12.149--11.045) | <0.001 |
| Male | Year, knot number 1 | 17.212 (9.339-31.436) | 2.846 (2.234-3.448) | <0.001 |
| Male | Year, knot number 2 | 7.559 (4.220-13.488) | 2.023 (1.440-2.602) | <0.001 |
| Male | Year, knot number 3 | 30.239 (7.815-113.362) | 3.409 (2.056-4.731) | <0.001 |
| Male | Year, knot number 4 | 13.608 (8.755-21.369) | 2.611 (2.170-3.062) | <0.001 |

## Model 2: Negative binomial regression of year x cluster by sex

### Predictions

|  |  | Female | | |  | Male | | |
| --- | --- | --- | --- | --- | --- | --- | --- | --- |
| Year | Cluster | Predicted rate | Lower 95%CI | Upper 95%CI | | Predicted rate | Lower 95%CI | Upper 95%CI |
| 2003 | 1 | 0.00751 | 0.00259 | 0.02181 |  | 0.01162 | 0.00403 | 0.03355 |
| 2004 | 1 | 0.00908 | 0.00386 | 0.02135 |  | 0.01178 | 0.00512 | 0.02713 |
| 2005 | 1 | 0.01096 | 0.00558 | 0.02154 |  | 0.01205 | 0.00636 | 0.02280 |
| 2006 | 1 | 0.01321 | 0.00765 | 0.02281 |  | 0.01251 | 0.00762 | 0.02055 |
| 2007 | 1 | 0.01587 | 0.00980 | 0.02571 |  | 0.01331 | 0.00864 | 0.02051 |
| 2008 | 1 | 0.01899 | 0.01183 | 0.03050 |  | 0.01462 | 0.00944 | 0.02264 |
| 2009 | 1 | 0.02261 | 0.01391 | 0.03676 |  | 0.01672 | 0.01048 | 0.02667 |
| 2010 | 1 | 0.02675 | 0.01647 | 0.04345 |  | 0.02005 | 0.01238 | 0.03248 |
| 2011 | 1 | 0.03143 | 0.01999 | 0.04942 |  | 0.02543 | 0.01602 | 0.04037 |
| 2012 | 1 | 0.03662 | 0.02461 | 0.05447 |  | 0.03413 | 0.02259 | 0.05155 |
| 2013 | 1 | 0.04226 | 0.02931 | 0.06093 |  | 0.04695 | 0.03219 | 0.06850 |
| 2013 | 1 | 0.04226 | 0.02931 | 0.06093 |  | 0.04695 | 0.03219 | 0.06850 |
| 2014 | 1 | 0.04827 | 0.03275 | 0.07114 |  | 0.06373 | 0.04325 | 0.09390 |
| 2015 | 1 | 0.05449 | 0.03547 | 0.08373 |  | 0.08206 | 0.05403 | 0.12465 |
| 2016 | 1 | 0.06074 | 0.03925 | 0.09399 |  | 0.09644 | 0.06346 | 0.14656 |
| 2017 | 1 | 0.06681 | 0.04522 | 0.09872 |  | 0.10106 | 0.06979 | 0.14633 |
| 2018 | 1 | 0.07276 | 0.05118 | 0.10344 |  | 0.09808 | 0.07023 | 0.13696 |
| 2019 | 1 | 0.07876 | 0.05402 | 0.11482 |  | 0.09302 | 0.06467 | 0.13379 |
| 2020 | 1 | 0.08506 | 0.05539 | 0.13060 |  | 0.09096 | 0.05997 | 0.13795 |
| 2021 | 1 | 0.09202 | 0.05962 | 0.14201 |  | 0.09675 | 0.06361 | 0.14716 |
| 2022 | 1 | 0.10001 | 0.06818 | 0.14670 |  | 0.11616 | 0.08049 | 0.16764 |
| 2023 | 1 | 0.10911 | 0.07155 | 0.16639 |  | 0.15288 | 0.10115 | 0.23106 |
| 2024 | 1 | 0.11926 | 0.06328 | 0.22478 |  | 0.21065 | 0.11158 | 0.39766 |
| 2003 | 2 | 0.01130 | 0.00343 | 0.03718 |  | 0.00668 | 0.00206 | 0.02166 |
| 2004 | 2 | 0.01420 | 0.00549 | 0.03670 |  | 0.00791 | 0.00313 | 0.01997 |
| 2005 | 2 | 0.01782 | 0.00853 | 0.03726 |  | 0.00938 | 0.00462 | 0.01908 |
| 2006 | 2 | 0.02231 | 0.01254 | 0.03969 |  | 0.01116 | 0.00640 | 0.01945 |
| 2007 | 2 | 0.02780 | 0.01707 | 0.04526 |  | 0.01332 | 0.00817 | 0.02172 |
| 2008 | 2 | 0.03444 | 0.02155 | 0.05505 |  | 0.01598 | 0.00972 | 0.02628 |
| 2009 | 2 | 0.04237 | 0.02612 | 0.06873 |  | 0.01929 | 0.01134 | 0.03281 |
| 2010 | 2 | 0.05167 | 0.03166 | 0.08435 |  | 0.02345 | 0.01359 | 0.04048 |
| 2011 | 2 | 0.06239 | 0.03926 | 0.09913 |  | 0.02877 | 0.01713 | 0.04830 |
| 2012 | 2 | 0.07445 | 0.04954 | 0.11190 |  | 0.03556 | 0.02256 | 0.05606 |
| 2013 | 2 | 0.08765 | 0.06056 | 0.12686 |  | 0.04391 | 0.02922 | 0.06599 |
| 2014 | 2 | 0.10156 | 0.06918 | 0.14909 |  | 0.05358 | 0.03541 | 0.08108 |
| 2015 | 2 | 0.11558 | 0.07578 | 0.17627 |  | 0.06389 | 0.04072 | 0.10024 |
| 2016 | 2 | 0.12890 | 0.08389 | 0.19805 |  | 0.07365 | 0.04666 | 0.11624 |
| 2017 | 2 | 0.14075 | 0.09576 | 0.20689 |  | 0.08149 | 0.05413 | 0.12270 |
| 2018 | 2 | 0.15114 | 0.10666 | 0.21416 |  | 0.08734 | 0.06005 | 0.12704 |
| 2019 | 2 | 0.16047 | 0.11038 | 0.23329 |  | 0.09184 | 0.06093 | 0.13843 |
| 2020 | 2 | 0.16940 | 0.11078 | 0.25905 |  | 0.09598 | 0.05995 | 0.15367 |
| 2020 | 2 | 0.16940 | 0.11078 | 0.25905 |  | 0.09598 | 0.05995 | 0.15367 |
| 2021 | 2 | 0.17880 | 0.11658 | 0.27424 |  | 0.10099 | 0.06276 | 0.16252 |
| 2022 | 2 | 0.18952 | 0.13020 | 0.27587 |  | 0.10802 | 0.07149 | 0.16323 |
| 2023 | 2 | 0.20160 | 0.13277 | 0.30611 |  | 0.11707 | 0.07554 | 0.18141 |
| 2024 | 2 | 0.21483 | 0.11384 | 0.40541 |  | 0.12770 | 0.06622 | 0.24627 |
| 2003 | 3 | 0.00823 | 0.00220 | 0.03086 |  | 0.02412 | 0.00833 | 0.06986 |
| 2004 | 3 | 0.01012 | 0.00349 | 0.02934 |  | 0.02437 | 0.01050 | 0.05653 |
| 2005 | 3 | 0.01243 | 0.00540 | 0.02862 |  | 0.02481 | 0.01292 | 0.04767 |
| 2006 | 3 | 0.01521 | 0.00793 | 0.02918 |  | 0.02566 | 0.01522 | 0.04326 |
| 2007 | 3 | 0.01852 | 0.01082 | 0.03170 |  | 0.02716 | 0.01701 | 0.04339 |
| 2008 | 3 | 0.02241 | 0.01366 | 0.03675 |  | 0.02965 | 0.01841 | 0.04776 |
| 2009 | 3 | 0.02689 | 0.01640 | 0.04409 |  | 0.03366 | 0.02033 | 0.05571 |
| 2010 | 3 | 0.03196 | 0.01948 | 0.05242 |  | 0.04001 | 0.02393 | 0.06689 |
| 2011 | 3 | 0.03755 | 0.02351 | 0.05999 |  | 0.05022 | 0.03085 | 0.08177 |
| 2012 | 3 | 0.04357 | 0.02865 | 0.06626 |  | 0.06653 | 0.04312 | 0.10264 |
| 2013 | 3 | 0.04991 | 0.03373 | 0.07387 |  | 0.09002 | 0.06036 | 0.13424 |
| 2014 | 3 | 0.05647 | 0.03732 | 0.08543 |  | 0.11946 | 0.07883 | 0.18103 |
| 2015 | 3 | 0.06311 | 0.04011 | 0.09930 |  | 0.14927 | 0.09495 | 0.23468 |
| 2016 | 3 | 0.06969 | 0.04416 | 0.10999 |  | 0.16863 | 0.10689 | 0.26602 |
| 2017 | 3 | 0.07609 | 0.05075 | 0.11409 |  | 0.16828 | 0.11211 | 0.25258 |
| 2018 | 3 | 0.08234 | 0.05711 | 0.11871 |  | 0.15548 | 0.10789 | 0.22407 |
| 2019 | 3 | 0.08854 | 0.05953 | 0.13167 |  | 0.14191 | 0.09600 | 0.20978 |
| 2020 | 3 | 0.09487 | 0.06025 | 0.14939 |  | 0.13648 | 0.08758 | 0.21267 |
| 2021 | 3 | 0.10157 | 0.06422 | 0.16064 |  | 0.14754 | 0.09424 | 0.23099 |
| 2022 | 3 | 0.10889 | 0.07287 | 0.16272 |  | 0.18731 | 0.12566 | 0.27920 |
| 2023 | 3 | 0.11689 | 0.07501 | 0.18213 |  | 0.26841 | 0.17096 | 0.42141 |
| 2024 | 3 | 0.12555 | 0.06404 | 0.24614 |  | 0.40862 | 0.20675 | 0.80761 |
| 2003 | 4 | 0.00679 | 0.00178 | 0.02586 |  | 0.00326 | 0.00079 | 0.01349 |
| 2004 | 4 | 0.00888 | 0.00306 | 0.02576 |  | 0.00431 | 0.00138 | 0.01345 |
| 2005 | 4 | 0.01162 | 0.00511 | 0.02644 |  | 0.00572 | 0.00235 | 0.01389 |
| 2006 | 4 | 0.01520 | 0.00806 | 0.02865 |  | 0.00760 | 0.00379 | 0.01525 |
| 2007 | 4 | 0.01985 | 0.01173 | 0.03360 |  | 0.01015 | 0.00564 | 0.01826 |
| 2008 | 4 | 0.02591 | 0.01571 | 0.04273 |  | 0.01363 | 0.00779 | 0.02385 |
| 2009 | 4 | 0.03378 | 0.02016 | 0.05661 |  | 0.01844 | 0.01041 | 0.03266 |
| 2010 | 4 | 0.04396 | 0.02600 | 0.07433 |  | 0.02514 | 0.01413 | 0.04473 |
| 2011 | 4 | 0.05712 | 0.03475 | 0.09390 |  | 0.03462 | 0.02013 | 0.05955 |
| 2012 | 4 | 0.07391 | 0.04788 | 0.11410 |  | 0.04804 | 0.02995 | 0.07705 |
| 2013 | 4 | 0.09425 | 0.06407 | 0.13864 |  | 0.06598 | 0.04339 | 0.10033 |
| 2014 | 4 | 0.11694 | 0.07888 | 0.17338 |  | 0.08785 | 0.05736 | 0.13454 |
| 2015 | 4 | 0.13942 | 0.09047 | 0.21484 |  | 0.11099 | 0.06952 | 0.17717 |
| 2016 | 4 | 0.15770 | 0.10135 | 0.24538 |  | 0.13027 | 0.08068 | 0.21035 |
| 2017 | 4 | 0.16821 | 0.11274 | 0.25098 |  | 0.14028 | 0.09102 | 0.21620 |
| 2018 | 4 | 0.17258 | 0.11908 | 0.25010 |  | 0.14165 | 0.09590 | 0.20922 |
| 2019 | 4 | 0.17485 | 0.11633 | 0.26280 |  | 0.13826 | 0.09150 | 0.20892 |
| 2020 | 4 | 0.17961 | 0.11293 | 0.28565 |  | 0.13448 | 0.08447 | 0.21410 |
| 2021 | 4 | 0.19205 | 0.12062 | 0.30578 |  | 0.13439 | 0.08426 | 0.21434 |
| 2022 | 4 | 0.21770 | 0.14491 | 0.32705 |  | 0.14106 | 0.09420 | 0.21125 |
| 2023 | 4 | 0.25795 | 0.16331 | 0.40743 |  | 0.15385 | 0.09960 | 0.23764 |
| 2024 | 4 | 0.31248 | 0.15524 | 0.62900 |  | 0.17104 | 0.08839 | 0.33095 |
| 2003 | 5 | 0.01154 | 0.00390 | 0.03414 |  | 0.00597 | 0.00189 | 0.01889 |
| 2003 | 5 | 0.01154 | 0.00390 | 0.03414 |  | 0.00597 | 0.00189 | 0.01889 |
| 2004 | 5 | 0.01421 | 0.00597 | 0.03382 |  | 0.00739 | 0.00300 | 0.01818 |
| 2005 | 5 | 0.01745 | 0.00880 | 0.03460 |  | 0.00912 | 0.00460 | 0.01808 |
| 2006 | 5 | 0.02136 | 0.01223 | 0.03730 |  | 0.01125 | 0.00663 | 0.01911 |
| 2007 | 5 | 0.02600 | 0.01574 | 0.04295 |  | 0.01385 | 0.00870 | 0.02204 |
| 2008 | 5 | 0.03142 | 0.01901 | 0.05194 |  | 0.01700 | 0.01054 | 0.02741 |
| 2009 | 5 | 0.03764 | 0.02235 | 0.06338 |  | 0.02079 | 0.01240 | 0.03485 |
| 2010 | 5 | 0.04459 | 0.02643 | 0.07522 |  | 0.02532 | 0.01482 | 0.04327 |
| 2011 | 5 | 0.05217 | 0.03199 | 0.08509 |  | 0.03068 | 0.01837 | 0.05126 |
| 2012 | 5 | 0.06021 | 0.03924 | 0.09238 |  | 0.03693 | 0.02344 | 0.05820 |
| 2013 | 5 | 0.06862 | 0.04653 | 0.10120 |  | 0.04393 | 0.02919 | 0.06612 |
| 2014 | 5 | 0.07741 | 0.05173 | 0.11584 |  | 0.05130 | 0.03392 | 0.07759 |
| 2015 | 5 | 0.08661 | 0.05572 | 0.13462 |  | 0.05844 | 0.03739 | 0.09135 |
| 2016 | 5 | 0.09630 | 0.06164 | 0.15043 |  | 0.06454 | 0.04112 | 0.10132 |
| 2017 | 5 | 0.10659 | 0.07158 | 0.15871 |  | 0.06891 | 0.04614 | 0.10291 |
| 2018 | 5 | 0.11748 | 0.08176 | 0.16881 |  | 0.07196 | 0.05045 | 0.10265 |
| 2019 | 5 | 0.12894 | 0.08677 | 0.19159 |  | 0.07464 | 0.05136 | 0.10846 |
| 2020 | 5 | 0.14090 | 0.08941 | 0.22204 |  | 0.07807 | 0.05114 | 0.11917 |
| 2021 | 5 | 0.15331 | 0.09667 | 0.24313 |  | 0.08362 | 0.05462 | 0.12802 |
| 2022 | 5 | 0.16612 | 0.11088 | 0.24887 |  | 0.09269 | 0.06407 | 0.13411 |
| 2023 | 5 | 0.17947 | 0.11611 | 0.27740 |  | 0.10546 | 0.07042 | 0.15794 |
| 2024 | 5 | 0.19361 | 0.10098 | 0.37118 |  | 0.12157 | 0.06549 | 0.22565 |

### Model estimates

| Model | Term | IRR_95_CI | Estimate_95_CI | p_value_formatted |
| --- | --- | --- | --- | --- |
| Female | (Intercept) | 0.000 (0.000-0.000) | -11.800 (-12.898--10.618) | <0.001 |
| Female | ns(YEAR, df = knots_number)1 | 8.574 (2.299-30.938) | 2.149 (0.833-3.432) | <0.001 |
| Female | ns(YEAR, df = knots_number)2 | 8.429 (2.523-27.953) | 2.132 (0.925-3.331) | <0.001 |
| Female | ns(YEAR, df = knots_number)3 | 44.676 (2.987-589.151) | 3.799 (1.094-6.379) | 0.003 |
| Female | ns(YEAR, df = knots_number)4 | 7.017 (2.851-18.128) | 1.948 (1.048-2.897) | <0.001 |
| Female | cluster2 | 1.505 (0.276-8.172) | 0.409 (-1.286-2.101) | 0.616 |
| Female | cluster3 | 1.096 (0.202-6.356) | 0.092 (-1.598-1.849) | 0.915 |
| Female | cluster4 | 0.904 (0.154-5.399) | -0.101 (-1.873-1.686) | 0.908 |
| Female | cluster5 | 1.538 (0.315-7.696) | 0.430 (-1.157-2.041) | 0.579 |
| Female | ns(YEAR, df = knots_number)1#cluster2 | 1.461 (0.227-9.370) | 0.379 (-1.484-2.238) | 0.665 |
| Female | ns(YEAR, df = knots_number)2#cluster2 | 1.234 (0.216-7.050) | 0.210 (-1.530-1.953) | 0.803 |
| Female | ns(YEAR, df = knots_number)3#cluster2 | 1.547 (0.031-79.126) | 0.436 (-3.478-4.371) | 0.821 |
| Female | ns(YEAR, df = knots_number)4#cluster2 | 1.012 (0.277-3.696) | 0.012 (-1.284-1.307) | 0.985 |
| Female | ns(YEAR, df = knots_number)1#cluster3 | 1.037 (0.149-6.969) | 0.036 (-1.902-1.941) | 0.969 |
| Female | ns(YEAR, df = knots_number)2#cluster3 | 0.996 (0.167-5.796) | -0.004 (-1.790-1.757) | 0.997 |
| Female | ns(YEAR, df = knots_number)3#cluster3 | 1.082 (0.019-53.531) | 0.078 (-3.942-3.980) | 0.969 |
| Female | ns(YEAR, df = knots_number)4#cluster3 | 0.892 (0.236-3.386) | -0.114 (-1.444-1.220) | 0.858 |
| Female | ns(YEAR, df = knots_number)1#cluster4 | 3.555 (0.512-24.356) | 1.268 (-0.670-3.193) | 0.165 |
| Female | ns(YEAR, df = knots_number)2#cluster4 | 1.728 (0.278-10.723) | 0.547 (-1.279-2.372) | 0.54 |
| Female | ns(YEAR, df = knots_number)3#cluster4 | 4.158 (0.068-254.343) | 1.425 (-2.694-5.539) | 0.488 |
| Female | ns(YEAR, df = knots_number)4#cluster4 | 2.062 (0.535-8.019) | 0.724 (-0.625-2.082) | 0.266 |
| Female | ns(YEAR, df = knots_number)1#cluster5 | 0.990 (0.157-6.210) | -0.010 (-1.854-1.826) | 0.991 |
| Female | ns(YEAR, df = knots_number)2#cluster5 | 1.078 (0.191-6.047) | 0.076 (-1.653-1.800) | 0.928 |
| Female | ns(YEAR, df = knots_number)3#cluster5 | 1.190 (0.028-49.018) | 0.174 (-3.587-3.892) | 0.925 |
| Female | ns(YEAR, df = knots_number)4#cluster5 | 0.978 (0.260-3.683) | -0.023 (-1.347-1.304) | 0.972 |
| Male | (Intercept) | 0.000 (0.000-0.000) | -11.363 (-12.408--10.234) | <0.001 |
| Male | ns(YEAR, df = knots_number)1 | 14.279 (4.275-46.173) | 2.659 (1.453-3.832) | <0.001 |
| Male | ns(YEAR, df = knots_number)2 | 5.331 (1.663-16.871) | 1.674 (0.509-2.826) | 0.003 |
| Male | ns(YEAR, df = knots_number)3 | 14.070 (0.933-189.804) | 2.644 (-0.069-5.246) | 0.045 |
| Male | ns(YEAR, df = knots_number)4 | 17.169 (7.061-43.999) | 2.843 (1.955-3.784) | <0.001 |
| Male | cluster2 | 0.574 (0.109-3.101) | -0.554 (-2.220-1.132) | 0.493 |
| Male | cluster3 | 2.076 (0.422-10.189) | 0.730 (-0.863-2.321) | 0.341 |
| Male | cluster4 | 0.280 (0.046-1.752) | -1.272 (-3.088-0.561) | 0.16 |
| Male | cluster5 | 0.514 (0.106-2.574) | -0.665 (-2.249-0.946) | 0.405 |
| Male | ns(YEAR, df = knots_number)1#cluster2 | 0.931 (0.155-5.561) | -0.071 (-1.865-1.716) | 0.934 |
| Male | ns(YEAR, df = knots_number)2#cluster2 | 1.977 (0.340-11.464) | 0.681 (-1.079-2.439) | 0.426 |
| Male | ns(YEAR, df = knots_number)3#cluster2 | 3.392 (0.052-214.366) | 1.221 (-2.959-5.368) | 0.536 |
| Male | ns(YEAR, df = knots_number)4#cluster2 | 0.538 (0.138-2.078) | -0.621 (-1.977-0.731) | 0.322 |
| Male | ns(YEAR, df = knots_number)1#cluster3 | 0.883 (0.146-5.324) | -0.124 (-1.922-1.672) | 0.882 |
| Male | ns(YEAR, df = knots_number)2#cluster3 | 0.646 (0.118-3.550) | -0.436 (-2.137-1.267) | 0.595 |
| Male | ns(YEAR, df = knots_number)3#cluster3 | 0.826 (0.018-37.423) | -0.191 (-3.997-3.622) | 0.919 |
| Male | ns(YEAR, df = knots_number)4#cluster3 | 0.949 (0.243-3.764) | -0.052 (-1.416-1.326) | 0.934 |
| Male | ns(YEAR, df = knots_number)1#cluster4 | 4.160 (0.587-29.202) | 1.426 (-0.533-3.374) | 0.129 |
| Male | ns(YEAR, df = knots_number)2#cluster4 | 4.201 (0.665-26.512) | 1.435 (-0.407-3.278) | 0.113 |
| Male | ns(YEAR, df = knots_number)3#cluster4 | 17.612 (0.224-1361.188) | 2.869 (-1.497-7.216) | 0.181 |
| Male | ns(YEAR, df = knots_number)4#cluster4 | 0.915 (0.227-3.683) | -0.089 (-1.483-1.304) | 0.891 |
| Male | ns(YEAR, df = knots_number)1#cluster5 | 0.884 (0.153-5.065) | -0.123 (-1.877-1.622) | 0.884 |
| Male | ns(YEAR, df = knots_number)2#cluster5 | 1.597 (0.296-8.550) | 0.468 (-1.219-2.146) | 0.572 |
| Male | ns(YEAR, df = knots_number)3#cluster5 | 4.390 (0.086-212.025) | 1.479 (-2.451-5.357) | 0.449 |
| Male | ns(YEAR, df = knots_number)4#cluster5 | 0.476 (0.128-1.755) | -0.743 (-2.053-0.563) | 0.224 |

## Model 3: Negative binomial regression of year x cluster x language, by sex

### Predictions

| Year | cluster | Language/Region | sex | Predicted rate | 95%CI lower | 95%CI upper |
| --- | --- | --- | --- | --- | --- | --- |
| 2003 | 1 | NL | female | 0.00515804 | 0.00143181 | 0.01858166 |
| 2004 | 1 | NL | female | 0.00684926 | 0.00245841 | 0.01908243 |
| 2005 | 1 | NL | female | 0.00907568 | 0.00406317 | 0.02027183 |
| 2006 | 1 | NL | female | 0.01197476 | 0.00629368 | 0.02278395 |
| 2007 | 1 | NL | female | 0.0156994 | 0.00894288 | 0.02756061 |
| 2008 | 1 | NL | female | 0.02040819 | 0.01175782 | 0.03542273 |
| 2009 | 1 | NL | female | 0.02624864 | 0.01489182 | 0.04626644 |
| 2010 | 1 | NL | female | 0.03333241 | 0.01889975 | 0.05878648 |
| 2011 | 1 | NL | female | 0.04170232 | 0.02450303 | 0.07097424 |
| 2012 | 1 | NL | female | 0.0512489 | 0.03191755 | 0.08228857 |
| 2013 | 1 | NL | female | 0.06146396 | 0.03910281 | 0.09661245 |
| 2014 | 1 | NL | female | 0.0714118 | 0.04354228 | 0.11711937 |
| 2015 | 1 | NL | female | 0.07978746 | 0.04588819 | 0.13872936 |
| 2016 | 1 | NL | female | 0.08509742 | 0.04843935 | 0.14949767 |
| 2017 | 1 | NL | female | 0.08647434 | 0.05200355 | 0.14379424 |
| 2018 | 1 | NL | female | 0.08540838 | 0.0534871 | 0.13638038 |
| 2019 | 1 | NL | female | 0.08409681 | 0.05076054 | 0.13932622 |
| 2020 | 1 | NL | female | 0.08467359 | 0.04805179 | 0.14920601 |
| 2021 | 1 | NL | female | 0.08941889 | 0.05091604 | 0.15703769 |
| 2022 | 1 | NL | female | 0.10075035 | 0.06146942 | 0.16513305 |
| 2023 | 1 | NL | female | 0.11918496 | 0.06754038 | 0.21031941 |
| 2024 | 1 | NL | female | 0.14446915 | 0.06020327 | 0.34668109 |
| 2003 | 5 | NL | female | 0.00884724 | 0.00254162 | 0.03079669 |
| 2004 | 5 | NL | female | 0.01174822 | 0.00437793 | 0.03152646 |
| 2005 | 5 | NL | female | 0.01555459 | 0.00721832 | 0.03351822 |
| 2006 | 5 | NL | female | 0.02047339 | 0.01103341 | 0.03799002 |
| 2007 | 5 | NL | female | 0.02671083 | 0.01527183 | 0.04671794 |
| 2008 | 5 | NL | female | 0.03444087 | 0.0194575 | 0.06096226 |
| 2009 | 5 | NL | female | 0.04375945 | 0.02393844 | 0.07999224 |
| 2010 | 5 | NL | female | 0.05462645 | 0.0295914 | 0.10084177 |
| 2011 | 5 | NL | female | 0.06680203 | 0.03741362 | 0.11927506 |
| 2012 | 5 | NL | female | 0.07981312 | 0.04767136 | 0.13362599 |
| 2013 | 5 | NL | female | 0.09302062 | 0.0576734 | 0.15003164 |
| 2014 | 5 | NL | female | 0.10562023 | 0.06395213 | 0.17443724 |
| 2015 | 5 | NL | female | 0.11668648 | 0.06748056 | 0.20177268 |
| 2016 | 5 | NL | female | 0.12526853 | 0.07214575 | 0.21750698 |
| 2017 | 5 | NL | female | 0.13090165 | 0.07965303 | 0.21512356 |
| 2018 | 5 | NL | female | 0.13496705 | 0.08465619 | 0.21517746 |
| 2019 | 5 | NL | female | 0.13959786 | 0.08306423 | 0.23460835 |
| 2020 | 5 | NL | female | 0.14726073 | 0.0815056 | 0.26606421 |
| 2021 | 5 | NL | female | 0.16107987 | 0.08896365 | 0.29165535 |
| 2022 | 5 | NL | female | 0.18469631 | 0.10831492 | 0.31494022 |
| 2023 | 5 | NL | female | 0.21936388 | 0.11728609 | 0.41028321 |
| 2024 | 5 | NL | female | 0.2651655 | 0.10227143 | 0.68751112 |
| 2003 | 3 | NL | female | 0.00683615 | 0.00115411 | 0.04049249 |
| 2004 | 3 | NL | female | 0.00912682 | 0.00219234 | 0.03799536 |
| 2005 | 3 | NL | female | 0.01214215 | 0.00400971 | 0.0367687 |
| 2006 | 3 | NL | female | 0.01604016 | 0.00685129 | 0.03755302 |
| 2007 | 3 | NL | female | 0.02096656 | 0.01057409 | 0.04157298 |
| 2008 | 3 | NL | female | 0.02702212 | 0.01452243 | 0.05028049 |
| 2009 | 3 | NL | female | 0.03421795 | 0.01835312 | 0.06379667 |
| 2010 | 3 | NL | female | 0.04242279 | 0.02259655 | 0.07964462 |
| 2011 | 3 | NL | female | 0.05131256 | 0.02814207 | 0.09356025 |
| 2012 | 3 | NL | female | 0.06037975 | 0.03532981 | 0.1031909 |
| 2013 | 3 | NL | female | 0.06911122 | 0.04221794 | 0.1131358 |
| 2014 | 3 | NL | female | 0.07699053 | 0.0461287 | 0.12850007 |
| 2015 | 3 | NL | female | 0.08352174 | 0.0477581 | 0.14606697 |
| 2016 | 3 | NL | female | 0.08828302 | 0.05017859 | 0.15532308 |
| 2017 | 3 | NL | female | 0.09117237 | 0.05484174 | 0.15157071 |
| 2018 | 3 | NL | female | 0.09305822 | 0.0579477 | 0.14944221 |
| 2019 | 3 | NL | female | 0.09516994 | 0.05634797 | 0.160739 |
| 2020 | 3 | NL | female | 0.09886587 | 0.05446411 | 0.17946608 |
| 2021 | 3 | NL | female | 0.10576518 | 0.05831202 | 0.19183476 |
| 2022 | 3 | NL | female | 0.11757275 | 0.06942319 | 0.19911718 |
| 2023 | 3 | NL | female | 0.13450418 | 0.07263078 | 0.24908688 |
| 2024 | 3 | NL | female | 0.15609805 | 0.06028017 | 0.40422251 |
| 2003 | 2 | NL | female | 0.00897832 | 0.00233518 | 0.03451991 |
| 2004 | 2 | NL | female | 0.01233243 | 0.00424593 | 0.0358199 |
| 2005 | 2 | NL | female | 0.01688387 | 0.00743438 | 0.03834417 |
| 2006 | 2 | NL | female | 0.02296332 | 0.01216111 | 0.04336067 |
| 2007 | 2 | NL | female | 0.03092477 | 0.0180341 | 0.05302962 |
| 2008 | 2 | NL | female | 0.04110145 | 0.02426323 | 0.06962507 |
| 2009 | 2 | NL | female | 0.0537349 | 0.03095195 | 0.0932878 |
| 2010 | 2 | NL | female | 0.06887698 | 0.03921842 | 0.12096455 |
| 2011 | 2 | NL | female | 0.08627397 | 0.05050169 | 0.14738512 |
| 2012 | 2 | NL | female | 0.10528582 | 0.06524347 | 0.16990366 |
| 2013 | 2 | NL | female | 0.12495328 | 0.0796786 | 0.19595378 |
| 2014 | 2 | NL | female | 0.14399353 | 0.08889187 | 0.23325121 |
| 2015 | 2 | NL | female | 0.16087396 | 0.09419942 | 0.27474086 |
| 2016 | 2 | NL | female | 0.17398262 | 0.1008793 | 0.30006107 |
| 2017 | 2 | NL | female | 0.18223721 | 0.11139489 | 0.29813218 |
| 2018 | 2 | NL | female | 0.18652134 | 0.11858159 | 0.29338628 |
| 2019 | 2 | NL | female | 0.18859638 | 0.11559362 | 0.30770379 |
| 2020 | 2 | NL | female | 0.19046033 | 0.10963677 | 0.33086653 |
| 2021 | 2 | NL | female | 0.19422054 | 0.11165522 | 0.33784015 |
| 2022 | 2 | NL | female | 0.20158935 | 0.12371962 | 0.32847068 |
| 2023 | 2 | NL | female | 0.21213957 | 0.12280581 | 0.36645821 |
| 2024 | 2 | NL | female | 0.22478463 | 0.09835873 | 0.51371269 |
| 2003 | 4 | NL | female | 0.00764955 | 0.00167829 | 0.03486611 |
| 2004 | 4 | NL | female | 0.00989257 | 0.00298496 | 0.03278535 |
| 2005 | 4 | NL | female | 0.01281563 | 0.00512535 | 0.03204472 |
| 2006 | 4 | NL | female | 0.01666043 | 0.00825785 | 0.03361286 |
| 2007 | 4 | NL | female | 0.02177232 | 0.01210836 | 0.03914929 |
| 2008 | 4 | NL | female | 0.02865189 | 0.01619423 | 0.05069278 |
| 2009 | 4 | NL | female | 0.03803552 | 0.02087776 | 0.06929388 |
| 2010 | 4 | NL | female | 0.05102354 | 0.02756886 | 0.09443269 |
| 2011 | 4 | NL | female | 0.06928743 | 0.03857585 | 0.12444957 |
| 2012 | 4 | NL | female | 0.09496008 | 0.05662057 | 0.15926043 |
| 2013 | 4 | NL | female | 0.12849953 | 0.08012017 | 0.20609206 |
| 2014 | 4 | NL | female | 0.16716668 | 0.10184895 | 0.27437396 |
| 2015 | 4 | NL | female | 0.20356323 | 0.11762219 | 0.35229737 |
| 2016 | 4 | NL | female | 0.22592494 | 0.12895418 | 0.39581562 |
| 2017 | 4 | NL | female | 0.22543322 | 0.135432 | 0.37524466 |
| 2018 | 4 | NL | female | 0.21017148 | 0.1305109 | 0.33845489 |
| 2019 | 4 | NL | female | 0.19275469 | 0.11428594 | 0.32510009 |
| 2020 | 4 | NL | female | 0.18309839 | 0.10150498 | 0.33027957 |
| 2021 | 4 | NL | female | 0.18966433 | 0.10542764 | 0.34120613 |
| 2022 | 4 | NL | female | 0.22193182 | 0.13184026 | 0.37358644 |
| 2023 | 4 | NL | female | 0.28474097 | 0.15435284 | 0.52527326 |
| 2024 | 4 | NL | female | 0.38254151 | 0.14871145 | 0.9840399 |
| 2003 | 1 | FR | female | 0.01594923 | 0.00258143 | 0.0985415 |
| 2004 | 1 | FR | female | 0.01461307 | 0.00334368 | 0.06386426 |
| 2005 | 1 | FR | female | 0.01346722 | 0.00410971 | 0.04413113 |
| 2006 | 1 | FR | female | 0.01255695 | 0.00466404 | 0.03380694 |
| 2007 | 1 | FR | female | 0.01191501 | 0.00483119 | 0.02938565 |
| 2008 | 1 | FR | female | 0.01157295 | 0.00471342 | 0.02841526 |
| 2009 | 1 | FR | female | 0.01157357 | 0.00461755 | 0.02900836 |
| 2010 | 1 | FR | female | 0.01198669 | 0.00482001 | 0.02980926 |
| 2011 | 1 | FR | female | 0.01293227 | 0.00556477 | 0.03005398 |
| 2012 | 1 | FR | female | 0.01459039 | 0.00707791 | 0.03007661 |
| 2013 | 1 | FR | female | 0.0171437 | 0.00916112 | 0.03208192 |
| 2014 | 1 | FR | female | 0.02085232 | 0.01122073 | 0.03875143 |
| 2015 | 1 | FR | female | 0.02609659 | 0.01337288 | 0.05092633 |
| 2016 | 1 | FR | female | 0.03340101 | 0.01697581 | 0.06571865 |
| 2017 | 1 | FR | female | 0.04334677 | 0.02385454 | 0.07876665 |
| 2018 | 1 | FR | female | 0.05598619 | 0.03333161 | 0.09403848 |
| 2019 | 1 | FR | female | 0.0704605 | 0.04084617 | 0.12154586 |
| 2020 | 1 | FR | female | 0.08459888 | 0.04498116 | 0.1591104 |
| 2021 | 1 | FR | female | 0.09487483 | 0.04930864 | 0.1825488 |
| 2022 | 1 | FR | female | 0.09813097 | 0.05486085 | 0.17552933 |
| 2023 | 1 | FR | female | 0.0956273 | 0.05205664 | 0.17566597 |
| 2024 | 1 | FR | female | 0.09045197 | 0.03735227 | 0.21903778 |
| 2003 | 5 | FR | female | 0.02209977 | 0.00295732 | 0.16514928 |
| 2004 | 5 | FR | female | 0.02167044 | 0.00413318 | 0.113619 |
| 2005 | 5 | FR | female | 0.02132014 | 0.00545488 | 0.08332877 |
| 2006 | 5 | FR | female | 0.02111529 | 0.00662723 | 0.0672763 |
| 2007 | 5 | FR | female | 0.02112179 | 0.00735014 | 0.06069684 |
| 2008 | 5 | FR | female | 0.02141085 | 0.00765474 | 0.05988767 |
| 2009 | 5 | FR | female | 0.02206726 | 0.00791623 | 0.06151464 |
| 2010 | 5 | FR | female | 0.02320155 | 0.00858389 | 0.06271185 |
| 2011 | 5 | FR | female | 0.02496788 | 0.01010946 | 0.06166448 |
| 2012 | 5 | FR | female | 0.02758063 | 0.01284791 | 0.05920739 |
| 2013 | 5 | FR | female | 0.03131295 | 0.01621883 | 0.06045445 |
| 2014 | 5 | FR | female | 0.03656787 | 0.01892902 | 0.07064334 |
| 2015 | 5 | FR | female | 0.04396329 | 0.02139037 | 0.09035707 |
| 2016 | 5 | FR | female | 0.05445707 | 0.02614903 | 0.11341041 |
| 2017 | 5 | FR | female | 0.06925379 | 0.03625651 | 0.13228211 |
| 2018 | 5 | FR | female | 0.08852826 | 0.05060537 | 0.15487 |
| 2019 | 5 | FR | female | 0.11088844 | 0.06165924 | 0.1994226 |
| 2020 | 5 | FR | female | 0.13267021 | 0.06714159 | 0.26215322 |
| 2021 | 5 | FR | female | 0.14779515 | 0.07321357 | 0.29835187 |
| 2022 | 5 | FR | female | 0.15088588 | 0.08249801 | 0.2759648 |
| 2023 | 5 | FR | female | 0.1444047 | 0.08007222 | 0.26042384 |
| 2024 | 5 | FR | female | 0.13380927 | 0.05655447 | 0.31659606 |
| 2003 | 3 | FR | female | 0.00913375 | 0.00135057 | 0.06177057 |
| 2004 | 3 | FR | female | 0.01009936 | 0.00214718 | 0.04750284 |
| 2005 | 3 | FR | female | 0.01116641 | 0.00326237 | 0.03822028 |
| 2006 | 3 | FR | female | 0.01234474 | 0.00459985 | 0.03312994 |
| 2007 | 3 | FR | female | 0.01364502 | 0.00587105 | 0.03171267 |
| 2008 | 3 | FR | female | 0.01507873 | 0.00681765 | 0.03334994 |
| 2009 | 3 | FR | female | 0.0166582 | 0.00754039 | 0.03680123 |
| 2010 | 3 | FR | female | 0.01839665 | 0.00839188 | 0.04032907 |
| 2011 | 3 | FR | female | 0.02030819 | 0.00971316 | 0.0424602 |
| 2012 | 3 | FR | female | 0.02243936 | 0.01156068 | 0.04355493 |
| 2013 | 3 | FR | female | 0.0249909 | 0.01326691 | 0.0470754 |
| 2014 | 3 | FR | female | 0.02828925 | 0.01432646 | 0.05586038 |
| 2015 | 3 | FR | female | 0.03282199 | 0.01556028 | 0.06923287 |
| 2016 | 3 | FR | female | 0.03935935 | 0.01865165 | 0.08305745 |
| 2017 | 3 | FR | female | 0.04889703 | 0.02551136 | 0.09371981 |
| 2018 | 3 | FR | female | 0.06157215 | 0.03520243 | 0.1076951 |
| 2019 | 3 | FR | female | 0.07642674 | 0.04264644 | 0.13696446 |
| 2020 | 3 | FR | female | 0.0909405 | 0.04647668 | 0.17794244 |
| 2021 | 3 | FR | female | 0.10088165 | 0.0506993 | 0.20073469 |
| 2022 | 3 | FR | female | 0.10249071 | 0.05636476 | 0.18636371 |
| 2023 | 3 | FR | female | 0.09753937 | 0.05257444 | 0.18096109 |
| 2024 | 3 | FR | female | 0.08984356 | 0.035852 | 0.22514407 |
| 2003 | 2 | FR | female | 0.01937237 | 0.0017436 | 0.21523818 |
| 2004 | 2 | FR | female | 0.01588169 | 0.00223861 | 0.11267145 |
| 2005 | 2 | FR | female | 0.01320033 | 0.00275657 | 0.06321218 |
| 2006 | 2 | FR | female | 0.01127772 | 0.00318616 | 0.03991857 |
| 2007 | 2 | FR | female | 0.01004109 | 0.00340232 | 0.02963379 |
| 2008 | 2 | FR | female | 0.00944578 | 0.0034215 | 0.02607706 |
| 2009 | 2 | FR | female | 0.00951845 | 0.00346951 | 0.02611344 |
| 2010 | 2 | FR | female | 0.01041694 | 0.00385461 | 0.0281514 |
| 2011 | 2 | FR | female | 0.01255262 | 0.00499677 | 0.031534 |
| 2012 | 2 | FR | female | 0.01673594 | 0.00763167 | 0.03670122 |
| 2013 | 2 | FR | female | 0.02393862 | 0.0123763 | 0.04630281 |
| 2014 | 2 | FR | female | 0.03530368 | 0.01888933 | 0.06598168 |
| 2015 | 2 | FR | female | 0.05158832 | 0.02652328 | 0.10034034 |
| 2016 | 2 | FR | female | 0.07178473 | 0.03649012 | 0.14121759 |
| 2017 | 2 | FR | female | 0.09231728 | 0.05058278 | 0.16848577 |
| 2018 | 2 | FR | female | 0.11077812 | 0.06542118 | 0.18758132 |
| 2019 | 2 | FR | female | 0.12646695 | 0.07266658 | 0.22009967 |
| 2020 | 2 | FR | female | 0.14005037 | 0.07419049 | 0.26437495 |
| 2021 | 2 | FR | female | 0.15339373 | 0.08037663 | 0.29274229 |
| 2022 | 2 | FR | female | 0.16879949 | 0.09615007 | 0.29634162 |
| 2023 | 2 | FR | female | 0.18679566 | 0.10086027 | 0.34595007 |
| 2024 | 2 | FR | female | 0.20729006 | 0.08050863 | 0.5337213 |
| 2003 | 4 | FR | female | 0.00443631 | 0.0002635 | 0.07468969 |
| 2004 | 4 | FR | female | 0.00572641 | 0.0005817 | 0.05637223 |
| 2005 | 4 | FR | female | 0.00737623 | 0.00121682 | 0.04471406 |
| 2006 | 4 | FR | female | 0.00946168 | 0.00231834 | 0.03861536 |
| 2007 | 4 | FR | female | 0.01206076 | 0.00385637 | 0.03771988 |
| 2008 | 4 | FR | female | 0.0152456 | 0.00550422 | 0.04222727 |
| 2009 | 4 | FR | female | 0.01907081 | 0.00704887 | 0.0515963 |
| 2010 | 4 | FR | female | 0.02355803 | 0.00879646 | 0.06309143 |
| 2011 | 4 | FR | female | 0.02867777 | 0.01141428 | 0.07205137 |
| 2012 | 4 | FR | female | 0.03439542 | 0.01561474 | 0.07576462 |
| 2013 | 4 | FR | female | 0.04094531 | 0.02105324 | 0.07963234 |
| 2014 | 4 | FR | female | 0.0488289 | 0.02589416 | 0.09207719 |
| 2015 | 4 | FR | female | 0.05887623 | 0.02981362 | 0.11626939 |
| 2016 | 4 | FR | female | 0.07244598 | 0.03609023 | 0.14542498 |
| 2017 | 4 | FR | female | 0.09135457 | 0.04878688 | 0.17106356 |
| 2018 | 4 | FR | female | 0.11618658 | 0.06577033 | 0.2052494 |
| 2019 | 4 | FR | female | 0.14593817 | 0.07850615 | 0.27129021 |
| 2020 | 4 | FR | female | 0.17727462 | 0.08671979 | 0.36238892 |
| 2021 | 4 | FR | female | 0.20392311 | 0.09875257 | 0.42109927 |
| 2022 | 4 | FR | female | 0.21920356 | 0.1173501 | 0.40946024 |
| 2023 | 4 | FR | female | 0.22406563 | 0.11596469 | 0.432937 |
| 2024 | 4 | FR | female | 0.22334494 | 0.08238206 | 0.60550758 |
| 2003 | 1 | NL | male | 0.01124503 | 0.00359321 | 0.03519157 |
| 2004 | 1 | NL | male | 0.01150599 | 0.00470208 | 0.02815513 |
| 2005 | 1 | NL | male | 0.01188386 | 0.00598212 | 0.02360805 |
| 2006 | 1 | NL | male | 0.0125064 | 0.0072549 | 0.0215592 |
| 2007 | 1 | NL | male | 0.01353686 | 0.00827815 | 0.02213619 |
| 2008 | 1 | NL | male | 0.01521198 | 0.00914079 | 0.02531558 |
| 2009 | 1 | NL | male | 0.01791454 | 0.01037989 | 0.03091851 |
| 2010 | 1 | NL | male | 0.02231764 | 0.01275046 | 0.03906346 |
| 2011 | 1 | NL | male | 0.0296882 | 0.01742392 | 0.05058504 |
| 2012 | 1 | NL | male | 0.04214227 | 0.02612412 | 0.06798204 |
| 2013 | 1 | NL | male | 0.06127666 | 0.03903545 | 0.09619024 |
| 2014 | 1 | NL | male | 0.08673507 | 0.05367025 | 0.14017024 |
| 2015 | 1 | NL | male | 0.11357857 | 0.06711313 | 0.19221414 |
| 2016 | 1 | NL | male | 0.13076142 | 0.07705897 | 0.22188916 |
| 2017 | 1 | NL | male | 0.12851929 | 0.0803171 | 0.20564996 |
| 2018 | 1 | NL | male | 0.11412057 | 0.07438129 | 0.17509115 |
| 2019 | 1 | NL | male | 0.09899479 | 0.06210672 | 0.15779242 |
| 2020 | 1 | NL | male | 0.0907109 | 0.05337437 | 0.15416512 |
| 2021 | 1 | NL | male | 0.09494042 | 0.05577975 | 0.16159418 |
| 2022 | 1 | NL | male | 0.11972413 | 0.074619 | 0.19209408 |
| 2023 | 1 | NL | male | 0.17379578 | 0.09989339 | 0.30237211 |
| 2024 | 1 | NL | male | 0.27068254 | 0.11527987 | 0.63557526 |
| 2003 | 5 | NL | male | 0.00582235 | 0.00143712 | 0.02358865 |
| 2004 | 5 | NL | male | 0.00722141 | 0.00240303 | 0.0217012 |
| 2005 | 5 | NL | male | 0.00895282 | 0.00385719 | 0.02078016 |
| 2006 | 5 | NL | male | 0.01108988 | 0.00575063 | 0.02138644 |
| 2007 | 5 | NL | male | 0.01371945 | 0.00772369 | 0.02436963 |
| 2008 | 5 | NL | male | 0.01694355 | 0.00945849 | 0.03035199 |
| 2009 | 5 | NL | male | 0.02088064 | 0.01119408 | 0.03894926 |
| 2010 | 5 | NL | male | 0.02566668 | 0.013497 | 0.04880928 |
| 2011 | 5 | NL | male | 0.03145548 | 0.0170293 | 0.05810264 |
| 2012 | 5 | NL | male | 0.0383695 | 0.0222735 | 0.06609731 |
| 2013 | 5 | NL | male | 0.04626941 | 0.02829326 | 0.07566674 |
| 2014 | 5 | NL | male | 0.05471706 | 0.03311657 | 0.09040661 |
| 2015 | 5 | NL | male | 0.06294705 | 0.03648445 | 0.10860328 |
| 2016 | 5 | NL | male | 0.0698803 | 0.04013936 | 0.12165754 |
| 2017 | 5 | NL | male | 0.07459781 | 0.0451574 | 0.12323194 |
| 2018 | 5 | NL | male | 0.07769693 | 0.0490841 | 0.12298916 |
| 2019 | 5 | NL | male | 0.08047569 | 0.04931196 | 0.13133402 |
| 2020 | 5 | NL | male | 0.08448615 | 0.04882953 | 0.14618018 |
| 2021 | 5 | NL | male | 0.0916311 | 0.05304519 | 0.15828503 |
| 2022 | 5 | NL | male | 0.10401334 | 0.06463885 | 0.16737263 |
| 2023 | 5 | NL | male | 0.12220174 | 0.07220497 | 0.2068177 |
| 2024 | 5 | NL | male | 0.14606188 | 0.06550003 | 0.32571088 |
| 2003 | 3 | NL | male | 0.03580654 | 0.01194193 | 0.10736188 |
| 2004 | 3 | NL | male | 0.03310296 | 0.01386531 | 0.07903219 |
| 2005 | 3 | NL | male | 0.0309383 | 0.01559629 | 0.06137218 |
| 2006 | 3 | NL | male | 0.02955129 | 0.01672216 | 0.05222285 |
| 2007 | 3 | NL | male | 0.02916299 | 0.01706518 | 0.04983715 |
| 2008 | 3 | NL | male | 0.03005995 | 0.0172097 | 0.05250533 |
| 2009 | 3 | NL | male | 0.03271677 | 0.01816316 | 0.05893179 |
| 2010 | 3 | NL | male | 0.03801051 | 0.02099625 | 0.06881225 |
| 2011 | 3 | NL | male | 0.04765552 | 0.02727891 | 0.08325293 |
| 2012 | 3 | NL | male | 0.06450204 | 0.0391868 | 0.1061713 |
| 2013 | 3 | NL | male | 0.09041818 | 0.05629528 | 0.14522438 |
| 2014 | 3 | NL | male | 0.12461801 | 0.07475805 | 0.20773212 |
| 2015 | 3 | NL | male | 0.16031317 | 0.09115604 | 0.28193759 |
| 2016 | 3 | NL | male | 0.18274337 | 0.103188 | 0.32363393 |
| 2017 | 3 | NL | male | 0.17921719 | 0.10706521 | 0.29999289 |
| 2018 | 3 | NL | male | 0.16062342 | 0.09982675 | 0.25844659 |
| 2019 | 3 | NL | male | 0.14292747 | 0.08540382 | 0.2391961 |
| 2020 | 3 | NL | male | 0.13717869 | 0.07684492 | 0.24488273 |
| 2021 | 3 | NL | male | 0.15427951 | 0.08626617 | 0.27591544 |
| 2022 | 3 | NL | male | 0.21495371 | 0.12696838 | 0.36391027 |
| 2023 | 3 | NL | male | 0.35178155 | 0.18926631 | 0.65384196 |
| 2024 | 3 | NL | male | 0.6239458 | 0.24419211 | 1.59427085 |
| 2003 | 2 | NL | male | 0.00743661 | 0.00195556 | 0.02827995 |
| 2004 | 2 | NL | male | 0.00782251 | 0.00273041 | 0.02241114 |
| 2005 | 2 | NL | male | 0.00830232 | 0.00367812 | 0.01874016 |
| 2006 | 2 | NL | male | 0.00897054 | 0.00466767 | 0.01723997 |
| 2007 | 2 | NL | male | 0.00995602 | 0.00550135 | 0.0180178 |
| 2008 | 2 | NL | male | 0.01145206 | 0.00621743 | 0.0210939 |
| 2009 | 2 | NL | male | 0.01377512 | 0.00720266 | 0.02634501 |
| 2010 | 2 | NL | male | 0.0174825 | 0.00904483 | 0.03379144 |
| 2011 | 2 | NL | male | 0.0236206 | 0.01270747 | 0.04390588 |
| 2012 | 2 | NL | male | 0.03396494 | 0.01970527 | 0.05854358 |
| 2013 | 2 | NL | male | 0.05007956 | 0.03040817 | 0.08247658 |
| 2014 | 2 | NL | male | 0.07227822 | 0.04274225 | 0.12222428 |
| 2015 | 2 | NL | male | 0.0974764 | 0.05456565 | 0.17413241 |
| 2016 | 2 | NL | male | 0.11726391 | 0.06503417 | 0.21144001 |
| 2017 | 2 | NL | male | 0.12229 | 0.07198687 | 0.20774407 |
| 2018 | 2 | NL | male | 0.11540178 | 0.07076193 | 0.18820249 |
| 2019 | 2 | NL | male | 0.1047185 | 0.06113336 | 0.17937774 |
| 2020 | 2 | NL | male | 0.09709974 | 0.052416 | 0.17987559 |
| 2021 | 2 | NL | male | 0.09776667 | 0.05246009 | 0.18220179 |
| 2022 | 2 | NL | male | 0.11155344 | 0.0648124 | 0.19200292 |
| 2023 | 2 | NL | male | 0.14003076 | 0.0782566 | 0.25056818 |
| 2024 | 2 | NL | male | 0.18436909 | 0.07723287 | 0.44012298 |
| 2003 | 4 | NL | male | 0.00262725 | 0.00056313 | 0.0122573 |
| 2004 | 4 | NL | male | 0.00369037 | 0.00108148 | 0.01259272 |
| 2005 | 4 | NL | male | 0.00518511 | 0.00199919 | 0.01344816 |
| 2006 | 4 | NL | male | 0.00728934 | 0.00345734 | 0.0153686 |
| 2007 | 4 | NL | male | 0.01025603 | 0.00544336 | 0.01932374 |
| 2008 | 4 | NL | male | 0.01444616 | 0.00783664 | 0.02663021 |
| 2009 | 4 | NL | male | 0.02037642 | 0.01083677 | 0.03831385 |
| 2010 | 4 | NL | male | 0.02878897 | 0.01520168 | 0.05452062 |
| 2011 | 4 | NL | male | 0.04075378 | 0.02232967 | 0.07437955 |
| 2012 | 4 | NL | male | 0.05761708 | 0.03402292 | 0.09757329 |
| 2013 | 4 | NL | male | 0.07996231 | 0.04967883 | 0.12870615 |
| 2014 | 4 | NL | male | 0.10669819 | 0.06503026 | 0.17506471 |
| 2015 | 4 | NL | male | 0.13407672 | 0.07752306 | 0.23188671 |
| 2016 | 4 | NL | male | 0.15540363 | 0.08857261 | 0.2726609 |
| 2017 | 4 | NL | male | 0.1643238 | 0.09832295 | 0.27462877 |
| 2018 | 4 | NL | male | 0.16301358 | 0.10084741 | 0.26350133 |
| 2019 | 4 | NL | male | 0.15754941 | 0.09332435 | 0.26597365 |
| 2020 | 4 | NL | male | 0.15405121 | 0.08536232 | 0.27801229 |
| 2021 | 4 | NL | male | 0.1582539 | 0.08767872 | 0.2856371 |
| 2022 | 4 | NL | male | 0.17540336 | 0.10421157 | 0.29522958 |
| 2023 | 4 | NL | male | 0.20603411 | 0.114937 | 0.36933324 |
| 2024 | 4 | NL | male | 0.24914331 | 0.10313617 | 0.60184888 |
| 2003 | 1 | FR | male | 0.01941665 | 0.00133693 | 0.28199438 |
| 2004 | 1 | FR | male | 0.01677239 | 0.00202709 | 0.13877695 |
| 2005 | 1 | FR | male | 0.01462929 | 0.00295417 | 0.0724456 |
| 2006 | 1 | FR | male | 0.0130097 | 0.00399605 | 0.04235486 |
| 2007 | 1 | FR | male | 0.01191061 | 0.0047647 | 0.02977371 |
| 2008 | 1 | FR | male | 0.01133526 | 0.00487398 | 0.02636208 |
| 2009 | 1 | FR | male | 0.01132316 | 0.00463129 | 0.02768428 |
| 2010 | 1 | FR | male | 0.01198809 | 0.00464604 | 0.03093265 |
| 2011 | 1 | FR | male | 0.0135827 | 0.00535301 | 0.03446466 |
| 2012 | 1 | FR | male | 0.01653544 | 0.00720292 | 0.03795973 |
| 2013 | 1 | FR | male | 0.02122782 | 0.01036243 | 0.04348598 |
| 2014 | 1 | FR | male | 0.02804489 | 0.01437088 | 0.05472984 |
| 2015 | 1 | FR | male | 0.03720996 | 0.01875365 | 0.07382996 |
| 2016 | 1 | FR | male | 0.0483861 | 0.02450045 | 0.09555801 |
| 2017 | 1 | FR | male | 0.06048842 | 0.0332687 | 0.10997875 |
| 2018 | 1 | FR | male | 0.07279346 | 0.04308177 | 0.12299605 |
| 2019 | 1 | FR | male | 0.08487799 | 0.04839688 | 0.1488582 |
| 2020 | 1 | FR | male | 0.09651492 | 0.05035333 | 0.1849953 |
| 2021 | 1 | FR | male | 0.10772205 | 0.05557355 | 0.20880505 |
| 2022 | 1 | FR | male | 0.11873087 | 0.06728287 | 0.20951871 |
| 2023 | 1 | FR | male | 0.12980936 | 0.07095488 | 0.23748149 |
| 2024 | 1 | FR | male | 0.14134809 | 0.05581242 | 0.357972 |
| 2003 | 5 | FR | male | 0.00657317 | 0.00092265 | 0.04682879 |
| 2004 | 5 | FR | male | 0.00789877 | 0.0017269 | 0.03612862 |
| 2005 | 5 | FR | male | 0.00948657 | 0.00304354 | 0.02956917 |
| 2006 | 5 | FR | male | 0.01138126 | 0.00478136 | 0.02709126 |
| 2007 | 5 | FR | male | 0.01363229 | 0.00633429 | 0.02933861 |
| 2008 | 5 | FR | male | 0.01629333 | 0.00724857 | 0.03662416 |
| 2009 | 5 | FR | male | 0.01942135 | 0.00793651 | 0.0475258 |
| 2010 | 5 | FR | male | 0.02307509 | 0.00901851 | 0.05904078 |
| 2011 | 5 | FR | male | 0.02731285 | 0.01105052 | 0.06750743 |
| 2012 | 5 | FR | male | 0.03217021 | 0.01444145 | 0.07166334 |
| 2013 | 5 | FR | male | 0.03757135 | 0.01845911 | 0.07647206 |
| 2014 | 5 | FR | male | 0.04332776 | 0.0214053 | 0.08770232 |
| 2015 | 5 | FR | male | 0.04913293 | 0.02315167 | 0.10427087 |
| 2016 | 5 | FR | male | 0.05455904 | 0.0256997 | 0.1158258 |
| 2017 | 5 | FR | male | 0.05918836 | 0.03081015 | 0.11370481 |
| 2018 | 5 | FR | male | 0.06304636 | 0.03644724 | 0.10905747 |
| 2019 | 5 | FR | male | 0.06639173 | 0.03786158 | 0.11642042 |
| 2020 | 5 | FR | male | 0.06959438 | 0.03637888 | 0.13313706 |
| 2021 | 5 | FR | male | 0.07311673 | 0.03761004 | 0.14214439 |
| 2022 | 5 | FR | male | 0.07739304 | 0.0436477 | 0.13722791 |
| 2023 | 5 | FR | male | 0.08241875 | 0.04465248 | 0.15212706 |
| 2024 | 5 | FR | male | 0.0880379 | 0.03411624 | 0.22718426 |
| 2003 | 3 | FR | male | 0.000604 | 2.0144E-05 | 0.01810992 |
| 2004 | 3 | FR | male | 0.00132742 | 8.8806E-05 | 0.01984147 |
| 2005 | 3 | FR | male | 0.00287001 | 0.00036683 | 0.02245409 |
| 2006 | 3 | FR | male | 0.00600566 | 0.00133665 | 0.02698379 |
| 2007 | 3 | FR | male | 0.01196584 | 0.00396655 | 0.03609721 |
| 2008 | 3 | FR | male | 0.02233224 | 0.00883524 | 0.05644767 |
| 2009 | 3 | FR | male | 0.03840864 | 0.01503448 | 0.09812269 |
| 2010 | 3 | FR | male | 0.05988735 | 0.02216001 | 0.16184539 |
| 2011 | 3 | FR | male | 0.08328223 | 0.03111262 | 0.22292976 |
| 2012 | 3 | FR | male | 0.10243417 | 0.04247692 | 0.24702261 |
| 2013 | 3 | FR | male | 0.11408486 | 0.05357158 | 0.24295259 |
| 2014 | 3 | FR | male | 0.11873529 | 0.05862174 | 0.24049217 |
| 2015 | 3 | FR | male | 0.11917313 | 0.05718363 | 0.24836188 |
| 2016 | 3 | FR | male | 0.11904238 | 0.05672099 | 0.24983851 |
| 2017 | 3 | FR | male | 0.12137461 | 0.06298337 | 0.23389975 |
| 2018 | 3 | FR | male | 0.12636773 | 0.07187752 | 0.22216686 |
| 2019 | 3 | FR | male | 0.1335691 | 0.07457984 | 0.23921616 |
| 2020 | 3 | FR | male | 0.14250055 | 0.07294676 | 0.27837299 |
| 2021 | 3 | FR | male | 0.15256228 | 0.07668963 | 0.30349931 |
| 2022 | 3 | FR | male | 0.16313718 | 0.0893319 | 0.29791976 |
| 2023 | 3 | FR | male | 0.17417734 | 0.09137987 | 0.33199596 |
| 2024 | 3 | FR | male | 0.18582188 | 0.07010051 | 0.49257515 |
| 2003 | 2 | FR | male | 0.00488635 | 0.00045374 | 0.05262089 |
| 2004 | 2 | FR | male | 0.00738425 | 0.00115076 | 0.04738368 |
| 2005 | 2 | FR | male | 0.01099357 | 0.00272408 | 0.04436677 |
| 2006 | 2 | FR | male | 0.01588519 | 0.00564145 | 0.04472951 |
| 2007 | 2 | FR | male | 0.02194713 | 0.00944856 | 0.05097884 |
| 2008 | 2 | FR | male | 0.02856308 | 0.01244389 | 0.06556226 |
| 2009 | 2 | FR | male | 0.03449729 | 0.01396072 | 0.08524369 |
| 2010 | 2 | FR | male | 0.0380915 | 0.0146294 | 0.09918128 |
| 2011 | 2 | FR | male | 0.03788305 | 0.01494854 | 0.0960044 |
| 2012 | 2 | FR | male | 0.03388992 | 0.01490099 | 0.07707719 |
| 2013 | 2 | FR | male | 0.02876328 | 0.01412159 | 0.05858593 |
| 2014 | 2 | FR | male | 0.02476319 | 0.01253204 | 0.04893182 |
| 2015 | 2 | FR | male | 0.0231224 | 0.01128023 | 0.04739667 |
| 2016 | 2 | FR | male | 0.02503661 | 0.01212924 | 0.05167938 |
| 2017 | 2 | FR | male | 0.0327171 | 0.0171266 | 0.06249978 |
| 2018 | 2 | FR | male | 0.048206 | 0.02703914 | 0.08594274 |
| 2019 | 2 | FR | male | 0.07282944 | 0.03928202 | 0.13502685 |
| 2020 | 2 | FR | male | 0.10259951 | 0.05062869 | 0.20791886 |
| 2021 | 2 | FR | male | 0.12256562 | 0.05990634 | 0.25076366 |
| 2022 | 2 | FR | male | 0.11637897 | 0.06273823 | 0.21588219 |
| 2023 | 2 | FR | male | 0.09292635 | 0.04844713 | 0.17824186 |
| 2024 | 2 | FR | male | 0.06804286 | 0.02542491 | 0.18209816 |
| 2003 | 4 | FR | male | 0.0178351 | 0.00041277 | 0.77063058 |
| 2004 | 4 | FR | male | 0.01521189 | 0.00072544 | 0.31898116 |
| 2005 | 4 | FR | male | 0.01313609 | 0.00120375 | 0.14334949 |
| 2006 | 4 | FR | male | 0.01162785 | 0.00181295 | 0.07457821 |
| 2007 | 4 | FR | male | 0.01068213 | 0.00236998 | 0.04814725 |
| 2008 | 4 | FR | male | 0.01031139 | 0.00266187 | 0.03994363 |
| 2009 | 4 | FR | male | 0.01058894 | 0.00277825 | 0.04035834 |
| 2010 | 4 | FR | male | 0.0117122 | 0.00307036 | 0.04467733 |
| 2011 | 4 | FR | male | 0.01412703 | 0.00398855 | 0.05003648 |
| 2012 | 4 | FR | male | 0.01865533 | 0.00625526 | 0.05563654 |
| 2013 | 4 | FR | male | 0.02617959 | 0.01049469 | 0.06530649 |
| 2014 | 4 | FR | male | 0.03757833 | 0.01614914 | 0.08744312 |
| 2015 | 4 | FR | male | 0.05310474 | 0.02191826 | 0.12866504 |
| 2016 | 4 | FR | male | 0.0711143 | 0.02883471 | 0.17538739 |
| 2017 | 4 | FR | male | 0.08760231 | 0.03926045 | 0.19546806 |
| 2018 | 4 | FR | male | 0.09970522 | 0.0508511 | 0.19549488 |
| 2019 | 4 | FR | male | 0.10621193 | 0.0550437 | 0.2049458 |
| 2020 | 4 | FR | male | 0.10727316 | 0.05135115 | 0.22409491 |
| 2021 | 4 | FR | male | 0.10405919 | 0.04908761 | 0.22059164 |
| 2022 | 4 | FR | male | 0.09814723 | 0.05192602 | 0.18551159 |
| 2023 | 4 | FR | male | 0.09089283 | 0.04779515 | 0.17285237 |
| 2024 | 4 | FR | male | 0.08340808 | 0.03106474 | 0.22394866 |

### Model estimates

| Model | Term | IRR_95_CI | Estimate_95_CI | p_value_formatted |
| --- | --- | --- | --- | --- |
| Female | (Intercept) | 0.000 (0.000-0.000) | -11.046 (-12.887--8.921) | <0.001 |
| Female | ns(YEAR, df = knots_number)1 | 1.956 (0.204-16.271) | 0.671 (-1.590-2.789) | 0.516 |
| Female | ns(YEAR, df = knots_number)2 | 8.169 (1.141-53.967) | 2.100 (0.132-3.988) | 0.022 |
| Female | ns(YEAR, df = knots_number)3 | 3.570 (0.030-267.108) | 1.273 (-3.513-5.588) | 0.559 |
| Female | ns(YEAR, df = knots_number)4 | 8.298 (1.862-39.117) | 2.116 (0.622-3.667) | 0.003 |
| Female | cluster2 | 1.215 (0.051-28.451) | 0.194 (-2.975-3.348) | 0.9 |
| Female | cluster3 | 0.573 (0.040-8.489) | -0.557 (-3.229-2.139) | 0.679 |
| Female | cluster4 | 0.278 (0.009-8.311) | -1.280 (-4.761-2.118) | 0.455 |
| Female | cluster5 | 1.386 (0.075-27.584) | 0.326 (-2.585-3.317) | 0.814 |
| Female | REGION:NL | 0.323 (0.027-3.308) | -1.129 (-3.607-1.196) | 0.32 |
| Female | ns(YEAR, df = knots_number)1#cluster2 | 2.741 (0.092-80.589) | 1.008 (-2.385-4.389) | 0.536 |
| Female | ns(YEAR, df = knots_number)2#cluster2 | 1.235 (0.066-23.433) | 0.211 (-2.717-3.154) | 0.884 |
| Female | ns(YEAR, df = knots_number)3#cluster2 | 0.822 (0.001-1043.227) | -0.196 (-7.247-6.950) | 0.956 |
| Female | ns(YEAR, df = knots_number)4#cluster2 | 3.062 (0.320-29.052) | 1.119 (-1.138-3.369) | 0.292 |
| Female | ns(YEAR, df = knots_number)1#cluster3 | 1.854 (0.086-39.971) | 0.617 (-2.459-3.688) | 0.682 |
| Female | ns(YEAR, df = knots_number)2#cluster3 | 1.412 (0.097-20.457) | 0.345 (-2.336-3.018) | 0.797 |
| Female | ns(YEAR, df = knots_number)3#cluster3 | 5.450 (0.012-2386.682) | 1.696 (-4.431-7.778) | 0.587 |
| Female | ns(YEAR, df = knots_number)4#cluster3 | 0.769 (0.094-6.307) | -0.262 (-2.359-1.842) | 0.79 |
| Female | ns(YEAR, df = knots_number)1#cluster4 | 7.382 (0.204-269.546) | 1.999 (-1.589-5.597) | 0.252 |
| Female | ns(YEAR, df = knots_number)2#cluster4 | 4.193 (0.182-104.011) | 1.433 (-1.705-4.644) | 0.364 |
| Female | ns(YEAR, df = knots_number)3#cluster4 | 64.446 (0.035-154981.871) | 4.166 (-3.359-11.951) | 0.284 |
| Female | ns(YEAR, df = knots_number)4#cluster4 | 2.020 (0.204-20.063) | 0.703 (-1.589-2.999) | 0.512 |
| Female | ns(YEAR, df = knots_number)1#cluster5 | 1.108 (0.040-29.213) | 0.103 (-3.228-3.375) | 0.948 |
| Female | ns(YEAR, df = knots_number)2#cluster5 | 1.033 (0.059-17.784) | 0.032 (-2.834-2.878) | 0.981 |
| Female | ns(YEAR, df = knots_number)3#cluster5 | 1.633 (0.002-1198.533) | 0.490 (-6.202-7.089) | 0.878 |
| Female | ns(YEAR, df = knots_number)4#cluster5 | 0.796 (0.090-6.871) | -0.228 (-2.408-1.927) | 0.827 |
| Female | ns(YEAR, df = knots_number)1#REGION:NL | 10.555 (0.737-163.871) | 2.357 (-0.305-5.099) | 0.064 |
| Female | ns(YEAR, df = knots_number)2#REGION:NL | 1.055 (0.094-12.502) | 0.054 (-2.369-2.526) | 0.964 |
| Female | ns(YEAR, df = knots_number)3#REGION:NL | 34.575 (0.153-10054.242) | 3.543 (-1.877-9.216) | 0.186 |
| Female | ns(YEAR, df = knots_number)4#REGION:NL | 0.995 (0.147-6.743) | -0.005 (-1.915-1.909) | 0.995 |
| Female | cluster2#REGION:NL | 1.433 (0.036-58.728) | 0.360 (-3.335-4.073) | 0.842 |
| Female | cluster3#REGION:NL | 2.314 (0.073-78.371) | 0.839 (-2.623-4.361) | 0.632 |
| Female | cluster4#REGION:NL | 5.332 (0.103-298.700) | 1.674 (-2.276-5.699) | 0.401 |
| Female | cluster5#REGION:NL | 1.238 (0.038-38.637) | 0.213 (-3.273-3.654) | 0.898 |
| Female | ns(YEAR, df = knots_number)1#cluster2#REGION:NL | 0.395 (0.007-22.364) | -0.930 (-4.964-3.107) | 0.631 |
| Female | ns(YEAR, df = knots_number)2#cluster2#REGION:NL | 1.134 (0.030-42.488) | 0.126 (-3.510-3.749) | 0.943 |
| Female | ns(YEAR, df = knots_number)3#cluster2#REGION:NL | 1.526 (0.000-6857.610) | 0.422 (-8.063-8.833) | 0.92 |
| Female | ns(YEAR, df = knots_number)4#cluster2#REGION:NL | 0.252 (0.016-3.990) | -1.378 (-4.136-1.384) | 0.297 |
| Female | ns(YEAR, df = knots_number)1#cluster3#REGION:NL | 0.375 (0.007-18.581) | -0.981 (-4.918-2.922) | 0.608 |
| Female | ns(YEAR, df = knots_number)2#cluster3#REGION:NL | 0.675 (0.019-23.287) | -0.393 (-3.959-3.148) | 0.824 |
| Female | ns(YEAR, df = knots_number)3#cluster3#REGION:NL | 0.162 (0.000-468.032) | -1.821 (-9.896-6.149) | 0.654 |
| Female | ns(YEAR, df = knots_number)4#cluster3#REGION:NL | 1.034 (0.069-15.413) | 0.034 (-2.667-2.735) | 0.979 |
| Female | ns(YEAR, df = knots_number)1#cluster4#REGION:NL | 0.319 (0.005-22.206) | -1.143 (-5.403-3.100) | 0.578 |
| Female | ns(YEAR, df = knots_number)2#cluster4#REGION:NL | 0.301 (0.006-14.103) | -1.200 (-5.099-2.646) | 0.532 |
| Female | ns(YEAR, df = knots_number)3#cluster4#REGION:NL | 0.021 (0.000-159.715) | -3.853 (-12.980-5.073) | 0.4 |
| Female | ns(YEAR, df = knots_number)4#cluster4#REGION:NL | 0.993 (0.058-17.109) | -0.007 (-2.850-2.840) | 0.996 |
| Female | ns(YEAR, df = knots_number)1#cluster5#REGION:NL | 0.701 (0.014-37.302) | -0.355 (-4.292-3.619) | 0.85 |
| Female | ns(YEAR, df = knots_number)2#cluster5#REGION:NL | 1.051 (0.030-37.322) | 0.050 (-3.511-3.620) | 0.977 |
| Female | ns(YEAR, df = knots_number)3#cluster5#REGION:NL | 0.658 (0.000-1899.589) | -0.418 (-8.346-7.549) | 0.914 |
| Female | ns(YEAR, df = knots_number)4#cluster5#REGION:NL | 1.344 (0.086-21.458) | 0.295 (-2.450-3.066) | 0.825 |
| Male | (Intercept) | 0.000 (0.000-0.000) | -10.849 (-13.515--7.937) | <0.001 |
| Male | ns(YEAR, df = knots_number)1 | 3.000 (0.187-39.891) | 1.099 (-1.678-3.686) | 0.392 |
| Male | ns(YEAR, df = knots_number)2 | 6.781 (0.544-76.604) | 1.914 (-0.609-4.339) | 0.11 |
| Male | ns(YEAR, df = knots_number)3 | 2.787 (0.004-1490.087) | 1.025 (-5.598-7.307) | 0.748 |
| Male | ns(YEAR, df = knots_number)4 | 13.762 (3.312-60.894) | 2.622 (1.198-4.109) | <0.001 |
| Male | cluster2 | 0.252 (0.006-10.901) | -1.380 (-5.187-2.389) | 0.45 |
| Male | cluster3 | 0.031 (0.000-2.345) | -3.470 (-7.889-0.852) | 0.116 |
| Male | cluster4 | 0.919 (0.007-223.862) | -0.085 (-4.981-5.411) | 0.971 |
| Male | cluster5 | 0.339 (0.010-11.340) | -1.083 (-4.651-2.428) | 0.522 |
| Male | REGION:NL | 0.579 (0.026-10.718) | -0.546 (-3.660-2.372) | 0.713 |
| Male | ns(YEAR, df = knots_number)1#cluster2 | 0.697 (0.018-28.064) | -0.361 (-4.008-3.335) | 0.835 |
| Male | ns(YEAR, df = knots_number)2#cluster2 | 3.473 (0.110-112.051) | 1.245 (-2.207-4.719) | 0.449 |
| Male | ns(YEAR, df = knots_number)3#cluster2 | 80.819 (0.009-723464.754) | 4.392 (-4.696-13.492) | 0.311 |
| Male | ns(YEAR, df = knots_number)4#cluster2 | 0.169 (0.020-1.451) | -1.775 (-3.927-0.372) | 0.077 |
| Male | ns(YEAR, df = knots_number)1#cluster3 | 58.772 (1.054-3417.716) | 4.074 (0.053-8.137) | 0.046 |
| Male | ns(YEAR, df = knots_number)2#cluster3 | 10.006 (0.232-453.910) | 2.303 (-1.461-6.118) | 0.221 |
| Male | ns(YEAR, df = knots_number)3#cluster3 | 11089.645 (0.489-330725846.541) | 9.314 (-0.716-19.617) | 0.067 |
| Male | ns(YEAR, df = knots_number)4#cluster3 | 0.746 (0.083-6.718) | -0.292 (-2.488-1.905) | 0.776 |
| Male | ns(YEAR, df = knots_number)1#cluster4 | 1.950 (0.010-230.328) | 0.668 (-4.606-5.440) | 0.768 |
| Male | ns(YEAR, df = knots_number)2#cluster4 | 1.233 (0.013-82.962) | 0.209 (-4.346-4.418) | 0.917 |
| Male | ns(YEAR, df = knots_number)3#cluster4 | 0.706 (0.000-49845.136) | -0.348 (-12.497-10.817) | 0.948 |
| Male | ns(YEAR, df = knots_number)4#cluster4 | 0.680 (0.058-7.772) | -0.386 (-2.842-2.051) | 0.745 |
| Male | ns(YEAR, df = knots_number)1#cluster5 | 3.072 (0.097-102.968) | 1.122 (-2.328-4.634) | 0.494 |
| Male | ns(YEAR, df = knots_number)2#cluster5 | 1.151 (0.044-30.586) | 0.140 (-3.117-3.421) | 0.928 |
| Male | ns(YEAR, df = knots_number)3#cluster5 | 13.338 (0.003-65292.010) | 2.591 (-5.852-11.087) | 0.523 |
| Male | ns(YEAR, df = knots_number)4#cluster5 | 0.441 (0.055-3.568) | -0.818 (-2.901-1.272) | 0.411 |
| Male | ns(YEAR, df = knots_number)1#REGION:NL | 8.195 (0.438-177.301) | 2.103 (-0.825-5.178) | 0.145 |
| Male | ns(YEAR, df = knots_number)2#REGION:NL | 0.647 (0.040-11.193) | -0.435 (-3.212-2.415) | 0.752 |
| Male | ns(YEAR, df = knots_number)3#REGION:NL | 6.220 (0.006-8288.779) | 1.828 (-5.107-9.023) | 0.602 |
| Male | ns(YEAR, df = knots_number)4#REGION:NL | 1.595 (0.246-10.466) | 0.467 (-1.402-2.348) | 0.6 |
| Male | cluster2#REGION:NL | 2.628 (0.040-179.257) | 0.966 (-3.219-5.189) | 0.635 |
| Male | cluster3#REGION:NL | 102.363 (0.991-11517.226) | 4.629 (-0.009-9.352) | 0.049 |
| Male | cluster4#REGION:NL | 0.254 (0.001-48.709) | -1.369 (-7.155-3.886) | 0.592 |
| Male | cluster5#REGION:NL | 1.529 (0.030-82.414) | 0.425 (-3.505-4.412) | 0.826 |
| Male | ns(YEAR, df = knots_number)1#cluster2#REGION:NL | 1.797 (0.026-119.334) | 0.586 (-3.649-4.782) | 0.769 |
| Male | ns(YEAR, df = knots_number)2#cluster2#REGION:NL | 0.532 (0.009-29.997) | -0.631 (-4.678-3.401) | 0.745 |
| Male | ns(YEAR, df = knots_number)3#cluster2#REGION:NL | 0.018 (0.000-473.401) | -4.027 (-14.234-6.160) | 0.408 |
| Male | ns(YEAR, df = knots_number)4#cluster2#REGION:NL | 5.396 (0.341-85.582) | 1.686 (-1.077-4.449) | 0.19 |
| Male | ns(YEAR, df = knots_number)1#cluster3#REGION:NL | 0.007 (0.000-0.664) | -4.925 (-9.475--0.410) | 0.029 |
| Male | ns(YEAR, df = knots_number)2#cluster3#REGION:NL | 0.053 (0.001-3.764) | -2.945 (-7.253-1.325) | 0.164 |
| Male | ns(YEAR, df = knots_number)3#cluster3#REGION:NL | 0.000 (0.000-1.613) | -10.370 (-21.455-0.478) | 0.058 |
| Male | ns(YEAR, df = knots_number)4#cluster3#REGION:NL | 1.502 (0.090-25.357) | 0.407 (-2.404-3.233) | 0.756 |
| Male | ns(YEAR, df = knots_number)1#cluster4#REGION:NL | 1.884 (0.010-541.735) | 0.633 (-4.607-6.295) | 0.8 |
| Male | ns(YEAR, df = knots_number)2#cluster4#REGION:NL | 4.960 (0.045-729.497) | 1.601 (-3.109-6.592) | 0.479 |
| Male | ns(YEAR, df = knots_number)3#cluster4#REGION:NL | 47.596 (0.000-20077225.492) | 3.863 (-8.241-16.815) | 0.51 |
| Male | ns(YEAR, df = knots_number)4#cluster4#REGION:NL | 1.473 (0.074-29.861) | 0.387 (-2.610-3.397) | 0.787 |
| Male | ns(YEAR, df = knots_number)1#cluster5#REGION:NL | 0.191 (0.003-10.645) | -1.657 (-5.731-2.365) | 0.389 |
| Male | ns(YEAR, df = knots_number)2#cluster5#REGION:NL | 1.806 (0.039-82.481) | 0.591 (-3.251-4.413) | 0.749 |
| Male | ns(YEAR, df = knots_number)3#cluster5#REGION:NL | 0.324 (0.000-4312.237) | -1.128 (-10.688-8.369) | 0.808 |
| Male | ns(YEAR, df = knots_number)4#cluster5#REGION:NL | 1.025 (0.070-14.778) | 0.024 (-2.658-2.693) | 0.985 |

## Model 4: Negative binomial regression of year x cluster x age groups, by sex

### Predictions

|  |  |  |  | Female | |  | Male | | |
| --- | --- | --- | --- | --- | --- | --- | --- | --- | --- |
| Year | cluster | Age group | Predicted rate | Lower 95%CI | Upper 95%CI | | Predicted rate | Lower 95%CI | Upper 95%CI |
| 2003 | 1 | 60-69 | 0.00376 | 0.00069 | 0.02041 |  | 0.00964 | 0.00404 | 0.02301 |
| 2004 | 1 | 60-69 | 0.00369 | 0.00092 | 0.01486 |  | 0.00869 | 0.00436 | 0.01729 |
| 2005 | 1 | 60-69 | 0.00364 | 0.00117 | 0.01136 |  | 0.00787 | 0.00450 | 0.01375 |
| 2006 | 1 | 60-69 | 0.00362 | 0.00140 | 0.00937 |  | 0.00721 | 0.00437 | 0.01190 |
| 2007 | 1 | 60-69 | 0.00366 | 0.00157 | 0.00849 |  | 0.00672 | 0.00403 | 0.01120 |
| 2008 | 1 | 60-69 | 0.00375 | 0.00168 | 0.00838 |  | 0.00640 | 0.00370 | 0.01107 |
| 2009 | 1 | 60-69 | 0.00394 | 0.00179 | 0.00869 |  | 0.00627 | 0.00354 | 0.01110 |
| 2010 | 1 | 60-69 | 0.00424 | 0.00198 | 0.00911 |  | 0.00634 | 0.00363 | 0.01109 |
| 2011 | 1 | 60-69 | 0.00471 | 0.00236 | 0.00943 |  | 0.00666 | 0.00402 | 0.01103 |
| 2012 | 1 | 60-69 | 0.00540 | 0.00302 | 0.00968 |  | 0.00721 | 0.00465 | 0.01117 |
| 2013 | 1 | 60-69 | 0.00631 | 0.00384 | 0.01036 |  | 0.00797 | 0.00534 | 0.01188 |
| 2014 | 1 | 60-69 | 0.00736 | 0.00449 | 0.01206 |  | 0.00892 | 0.00597 | 0.01332 |
| 2015 | 1 | 60-69 | 0.00844 | 0.00492 | 0.01448 |  | 0.01001 | 0.00660 | 0.01518 |
| 2016 | 1 | 60-69 | 0.00935 | 0.00542 | 0.01614 |  | 0.01119 | 0.00749 | 0.01670 |
| 2017 | 1 | 60-69 | 0.00990 | 0.00617 | 0.01590 |  | 0.01235 | 0.00877 | 0.01738 |
| 2018 | 1 | 60-69 | 0.01023 | 0.00684 | 0.01531 |  | 0.01348 | 0.01000 | 0.01818 |
| 2019 | 1 | 60-69 | 0.01059 | 0.00696 | 0.01613 |  | 0.01460 | 0.01062 | 0.02008 |
| 2020 | 1 | 60-69 | 0.01130 | 0.00709 | 0.01802 |  | 0.01574 | 0.01112 | 0.02228 |
| 2021 | 1 | 60-69 | 0.01268 | 0.00817 | 0.01969 |  | 0.01692 | 0.01224 | 0.02338 |
| 2022 | 1 | 60-69 | 0.01485 | 0.01028 | 0.02145 |  | 0.01816 | 0.01376 | 0.02396 |
| 2023 | 1 | 60-69 | 0.01789 | 0.01224 | 0.02616 |  | 0.01947 | 0.01423 | 0.02665 |
| 2024 | 1 | 60-69 | 0.02187 | 0.01259 | 0.03798 |  | 0.02087 | 0.01310 | 0.03325 |
| 2003 | 5 | 60-69 | 0.00650 | 0.00160 | 0.02649 |  | 0.00806 | 0.00317 | 0.02051 |
| 2004 | 5 | 60-69 | 0.00504 | 0.00158 | 0.01611 |  | 0.00651 | 0.00317 | 0.01335 |
| 2005 | 5 | 60-69 | 0.00396 | 0.00146 | 0.01074 |  | 0.00533 | 0.00297 | 0.00956 |
| 2006 | 5 | 60-69 | 0.00322 | 0.00127 | 0.00814 |  | 0.00448 | 0.00255 | 0.00789 |
| 2007 | 5 | 60-69 | 0.00274 | 0.00107 | 0.00700 |  | 0.00392 | 0.00209 | 0.00736 |
| 2008 | 5 | 60-69 | 0.00248 | 0.00093 | 0.00663 |  | 0.00361 | 0.00178 | 0.00736 |
| 2009 | 5 | 60-69 | 0.00243 | 0.00088 | 0.00668 |  | 0.00356 | 0.00167 | 0.00760 |
| 2010 | 5 | 60-69 | 0.00261 | 0.00097 | 0.00702 |  | 0.00380 | 0.00180 | 0.00801 |
| 2011 | 5 | 60-69 | 0.00313 | 0.00128 | 0.00764 |  | 0.00441 | 0.00226 | 0.00861 |
| 2012 | 5 | 60-69 | 0.00420 | 0.00203 | 0.00868 |  | 0.00543 | 0.00310 | 0.00952 |
| 2013 | 5 | 60-69 | 0.00595 | 0.00337 | 0.01049 |  | 0.00688 | 0.00430 | 0.01101 |
| 2014 | 5 | 60-69 | 0.00832 | 0.00502 | 0.01381 |  | 0.00867 | 0.00561 | 0.01340 |
| 2015 | 5 | 60-69 | 0.01073 | 0.00627 | 0.01837 |  | 0.01052 | 0.00676 | 0.01635 |
| 2016 | 5 | 60-69 | 0.01190 | 0.00686 | 0.02066 |  | 0.01189 | 0.00770 | 0.01834 |
| 2017 | 5 | 60-69 | 0.01091 | 0.00666 | 0.01786 |  | 0.01229 | 0.00837 | 0.01803 |
| 2018 | 5 | 60-69 | 0.00888 | 0.00561 | 0.01405 |  | 0.01206 | 0.00854 | 0.01702 |
| 2019 | 5 | 60-69 | 0.00711 | 0.00423 | 0.01196 |  | 0.01182 | 0.00824 | 0.01695 |
| 2020 | 5 | 60-69 | 0.00621 | 0.00346 | 0.01113 |  | 0.01217 | 0.00827 | 0.01792 |
| 2021 | 5 | 60-69 | 0.00638 | 0.00369 | 0.01103 |  | 0.01369 | 0.00959 | 0.01954 |
| 2022 | 5 | 60-69 | 0.00754 | 0.00480 | 0.01183 |  | 0.01661 | 0.01241 | 0.02221 |
| 2023 | 5 | 60-69 | 0.00978 | 0.00625 | 0.01533 |  | 0.02118 | 0.01576 | 0.02848 |
| 2024 | 5 | 60-69 | 0.01331 | 0.00697 | 0.02542 |  | 0.02771 | 0.01799 | 0.04269 |
| 2003 | 3 | 60-69 | 0.00339 | 0.00046 | 0.02492 |  | 0.00647 | 0.00251 | 0.01666 |
| 2004 | 3 | 60-69 | 0.00387 | 0.00079 | 0.01906 |  | 0.00701 | 0.00339 | 0.01451 |
| 2005 | 3 | 60-69 | 0.00442 | 0.00129 | 0.01512 |  | 0.00757 | 0.00433 | 0.01325 |
| 2006 | 3 | 60-69 | 0.00502 | 0.00197 | 0.01283 |  | 0.00811 | 0.00504 | 0.01307 |
| 2007 | 3 | 60-69 | 0.00567 | 0.00267 | 0.01205 |  | 0.00859 | 0.00531 | 0.01392 |
| 2008 | 3 | 60-69 | 0.00635 | 0.00318 | 0.01269 |  | 0.00896 | 0.00529 | 0.01518 |
| 2009 | 3 | 60-69 | 0.00703 | 0.00346 | 0.01428 |  | 0.00916 | 0.00524 | 0.01603 |
| 2010 | 3 | 60-69 | 0.00769 | 0.00372 | 0.01590 |  | 0.00916 | 0.00528 | 0.01589 |
| 2011 | 3 | 60-69 | 0.00828 | 0.00412 | 0.01664 |  | 0.00892 | 0.00538 | 0.01477 |
| 2012 | 3 | 60-69 | 0.00877 | 0.00473 | 0.01626 |  | 0.00850 | 0.00534 | 0.01351 |
| 2013 | 3 | 60-69 | 0.00914 | 0.00531 | 0.01572 |  | 0.00797 | 0.00498 | 0.01275 |
| 2014 | 3 | 60-69 | 0.00938 | 0.00553 | 0.01593 |  | 0.00739 | 0.00441 | 0.01240 |
| 2015 | 3 | 60-69 | 0.00951 | 0.00545 | 0.01659 |  | 0.00682 | 0.00391 | 0.01192 |
| 2016 | 3 | 60-69 | 0.00953 | 0.00550 | 0.01651 |  | 0.00630 | 0.00365 | 0.01087 |
| 2017 | 3 | 60-69 | 0.00947 | 0.00592 | 0.01514 |  | 0.00587 | 0.00364 | 0.00947 |
| 2018 | 3 | 60-69 | 0.00946 | 0.00635 | 0.01410 |  | 0.00563 | 0.00364 | 0.00870 |
| 2019 | 3 | 60-69 | 0.00967 | 0.00633 | 0.01478 |  | 0.00567 | 0.00355 | 0.00905 |
| 2020 | 3 | 60-69 | 0.01028 | 0.00640 | 0.01652 |  | 0.00613 | 0.00370 | 0.01015 |
| 2021 | 3 | 60-69 | 0.01149 | 0.00734 | 0.01798 |  | 0.00721 | 0.00453 | 0.01148 |
| 2022 | 3 | 60-69 | 0.01338 | 0.00922 | 0.01943 |  | 0.00910 | 0.00624 | 0.01328 |
| 2023 | 3 | 60-69 | 0.01603 | 0.01090 | 0.02357 |  | 0.01204 | 0.00829 | 0.01748 |
| 2024 | 3 | 60-69 | 0.01946 | 0.01106 | 0.03423 |  | 0.01628 | 0.00950 | 0.02790 |
| 2003 | 2 | 60-69 | 0.00383 | 0.00025 | 0.05764 |  | 0.00841 | 0.00248 | 0.02852 |
| 2004 | 2 | 60-69 | 0.00388 | 0.00042 | 0.03576 |  | 0.00770 | 0.00303 | 0.01958 |
| 2005 | 2 | 60-69 | 0.00395 | 0.00068 | 0.02295 |  | 0.00709 | 0.00354 | 0.01418 |
| 2006 | 2 | 60-69 | 0.00407 | 0.00106 | 0.01567 |  | 0.00660 | 0.00383 | 0.01138 |
| 2007 | 2 | 60-69 | 0.00426 | 0.00155 | 0.01174 |  | 0.00625 | 0.00375 | 0.01043 |
| 2008 | 2 | 60-69 | 0.00456 | 0.00208 | 0.00999 |  | 0.00605 | 0.00347 | 0.01055 |
| 2009 | 2 | 60-69 | 0.00501 | 0.00257 | 0.00975 |  | 0.00603 | 0.00331 | 0.01099 |
| 2010 | 2 | 60-69 | 0.00568 | 0.00306 | 0.01054 |  | 0.00621 | 0.00341 | 0.01130 |
| 2011 | 2 | 60-69 | 0.00668 | 0.00377 | 0.01183 |  | 0.00664 | 0.00388 | 0.01136 |
| 2012 | 2 | 60-69 | 0.00816 | 0.00499 | 0.01332 |  | 0.00729 | 0.00463 | 0.01146 |
| 2013 | 2 | 60-69 | 0.01008 | 0.00662 | 0.01535 |  | 0.00812 | 0.00546 | 0.01209 |
| 2014 | 2 | 60-69 | 0.01222 | 0.00802 | 0.01863 |  | 0.00909 | 0.00608 | 0.01360 |
| 2015 | 2 | 60-69 | 0.01412 | 0.00882 | 0.02258 |  | 0.01009 | 0.00655 | 0.01556 |
| 2016 | 2 | 60-69 | 0.01505 | 0.00926 | 0.02446 |  | 0.01098 | 0.00712 | 0.01694 |
| 2017 | 2 | 60-69 | 0.01460 | 0.00952 | 0.02238 |  | 0.01161 | 0.00785 | 0.01717 |
| 2018 | 2 | 60-69 | 0.01349 | 0.00937 | 0.01941 |  | 0.01193 | 0.00817 | 0.01743 |
| 2019 | 2 | 60-69 | 0.01263 | 0.00871 | 0.01833 |  | 0.01198 | 0.00783 | 0.01831 |
| 2020 | 2 | 60-69 | 0.01276 | 0.00849 | 0.01918 |  | 0.01178 | 0.00745 | 0.01865 |
| 2021 | 2 | 60-69 | 0.01457 | 0.00994 | 0.02135 |  | 0.01141 | 0.00751 | 0.01734 |
| 2022 | 2 | 60-69 | 0.01843 | 0.01340 | 0.02535 |  | 0.01093 | 0.00764 | 0.01562 |
| 2023 | 2 | 60-69 | 0.02499 | 0.01791 | 0.03486 |  | 0.01038 | 0.00683 | 0.01577 |
| 2024 | 2 | 60-69 | 0.03506 | 0.02151 | 0.05715 |  | 0.00981 | 0.00522 | 0.01844 |
| 2003 | 4 | 60-69 | 0.01064 | 0.00145 | 0.07799 |  | 0.00417 | 0.00061 | 0.02847 |
| 2004 | 4 | 60-69 | 0.00777 | 0.00163 | 0.03698 |  | 0.00373 | 0.00085 | 0.01634 |
| 2005 | 4 | 60-69 | 0.00576 | 0.00176 | 0.01883 |  | 0.00336 | 0.00109 | 0.01036 |
| 2006 | 4 | 60-69 | 0.00441 | 0.00177 | 0.01096 |  | 0.00307 | 0.00120 | 0.00783 |
| 2007 | 4 | 60-69 | 0.00353 | 0.00161 | 0.00777 |  | 0.00285 | 0.00113 | 0.00722 |
| 2008 | 4 | 60-69 | 0.00301 | 0.00135 | 0.00671 |  | 0.00272 | 0.00099 | 0.00749 |
| 2009 | 4 | 60-69 | 0.00277 | 0.00117 | 0.00658 |  | 0.00268 | 0.00091 | 0.00790 |
| 2010 | 4 | 60-69 | 0.00280 | 0.00115 | 0.00684 |  | 0.00273 | 0.00094 | 0.00798 |
| 2011 | 4 | 60-69 | 0.00315 | 0.00136 | 0.00731 |  | 0.00291 | 0.00111 | 0.00761 |
| 2012 | 4 | 60-69 | 0.00396 | 0.00195 | 0.00802 |  | 0.00319 | 0.00141 | 0.00725 |
| 2013 | 4 | 60-69 | 0.00527 | 0.00302 | 0.00921 |  | 0.00359 | 0.00173 | 0.00746 |
| 2014 | 4 | 60-69 | 0.00699 | 0.00427 | 0.01145 |  | 0.00407 | 0.00193 | 0.00860 |
| 2015 | 4 | 60-69 | 0.00864 | 0.00513 | 0.01458 |  | 0.00461 | 0.00206 | 0.01034 |
| 2016 | 4 | 60-69 | 0.00934 | 0.00541 | 0.01612 |  | 0.00516 | 0.00230 | 0.01158 |
| 2017 | 4 | 60-69 | 0.00851 | 0.00512 | 0.01414 |  | 0.00567 | 0.00281 | 0.01142 |
| 2018 | 4 | 60-69 | 0.00703 | 0.00426 | 0.01160 |  | 0.00617 | 0.00349 | 0.01090 |
| 2019 | 4 | 60-69 | 0.00582 | 0.00328 | 0.01033 |  | 0.00677 | 0.00402 | 0.01141 |
| 2020 | 4 | 60-69 | 0.00535 | 0.00284 | 0.01009 |  | 0.00760 | 0.00444 | 0.01300 |
| 2021 | 4 | 60-69 | 0.00589 | 0.00329 | 0.01057 |  | 0.00883 | 0.00539 | 0.01445 |
| 2022 | 4 | 60-69 | 0.00757 | 0.00477 | 0.01202 |  | 0.01055 | 0.00714 | 0.01560 |
| 2023 | 4 | 60-69 | 0.01078 | 0.00702 | 0.01655 |  | 0.01286 | 0.00889 | 0.01861 |
| 2024 | 4 | 60-69 | 0.01615 | 0.00868 | 0.03004 |  | 0.01583 | 0.00915 | 0.02738 |
| 2003 | 1 | 70-79 | 0.00646 | 0.00250 | 0.01669 |  | 0.00927 | 0.00316 | 0.02721 |
| 2004 | 1 | 70-79 | 0.00690 | 0.00325 | 0.01465 |  | 0.00969 | 0.00417 | 0.02250 |
| 2005 | 1 | 70-79 | 0.00737 | 0.00409 | 0.01330 |  | 0.01019 | 0.00539 | 0.01924 |
| 2006 | 1 | 70-79 | 0.00788 | 0.00485 | 0.01279 |  | 0.01080 | 0.00667 | 0.01750 |
| 2007 | 1 | 70-79 | 0.00842 | 0.00538 | 0.01319 |  | 0.01162 | 0.00774 | 0.01744 |
| 2008 | 1 | 70-79 | 0.00901 | 0.00568 | 0.01431 |  | 0.01273 | 0.00852 | 0.01901 |
| 2009 | 1 | 70-79 | 0.00965 | 0.00593 | 0.01572 |  | 0.01427 | 0.00936 | 0.02175 |
| 2010 | 1 | 70-79 | 0.01035 | 0.00632 | 0.01694 |  | 0.01644 | 0.01078 | 0.02509 |
| 2011 | 1 | 70-79 | 0.01111 | 0.00700 | 0.01764 |  | 0.01951 | 0.01328 | 0.02868 |
| 2012 | 1 | 70-79 | 0.01195 | 0.00799 | 0.01789 |  | 0.02356 | 0.01699 | 0.03267 |
| 2013 | 1 | 70-79 | 0.01293 | 0.00900 | 0.01857 |  | 0.02852 | 0.02153 | 0.03777 |
| 2014 | 1 | 70-79 | 0.01410 | 0.00973 | 0.02044 |  | 0.03408 | 0.02598 | 0.04471 |
| 2015 | 1 | 70-79 | 0.01558 | 0.01042 | 0.02330 |  | 0.03963 | 0.02984 | 0.05264 |
| 2016 | 1 | 70-79 | 0.01749 | 0.01169 | 0.02618 |  | 0.04417 | 0.03338 | 0.05844 |
| 2017 | 1 | 70-79 | 0.02000 | 0.01408 | 0.02841 |  | 0.04681 | 0.03670 | 0.05971 |
| 2018 | 1 | 70-79 | 0.02319 | 0.01708 | 0.03147 |  | 0.04823 | 0.03890 | 0.05978 |
| 2019 | 1 | 70-79 | 0.02712 | 0.01970 | 0.03733 |  | 0.04970 | 0.03952 | 0.06251 |
| 2020 | 1 | 70-79 | 0.03182 | 0.02248 | 0.04504 |  | 0.05276 | 0.04104 | 0.06783 |
| 2021 | 1 | 70-79 | 0.03728 | 0.02698 | 0.05149 |  | 0.05895 | 0.04664 | 0.07449 |
| 2022 | 1 | 70-79 | 0.04359 | 0.03313 | 0.05735 |  | 0.06881 | 0.05652 | 0.08377 |
| 2023 | 1 | 70-79 | 0.05091 | 0.03752 | 0.06908 |  | 0.08270 | 0.06688 | 0.10225 |
| 2024 | 1 | 70-79 | 0.05943 | 0.03776 | 0.09353 |  | 0.10085 | 0.07376 | 0.13791 |
| 2003 | 5 | 70-79 | 0.00736 | 0.00239 | 0.02268 |  | 0.01035 | 0.00366 | 0.02923 |
| 2004 | 5 | 70-79 | 0.00743 | 0.00303 | 0.01821 |  | 0.01043 | 0.00467 | 0.02328 |
| 2005 | 5 | 70-79 | 0.00752 | 0.00372 | 0.01519 |  | 0.01056 | 0.00578 | 0.01929 |
| 2006 | 5 | 70-79 | 0.00765 | 0.00432 | 0.01352 |  | 0.01080 | 0.00678 | 0.01720 |
| 2007 | 5 | 70-79 | 0.00784 | 0.00469 | 0.01309 |  | 0.01121 | 0.00739 | 0.01701 |
| 2008 | 5 | 70-79 | 0.00811 | 0.00484 | 0.01361 |  | 0.01186 | 0.00766 | 0.01836 |
| 2009 | 5 | 70-79 | 0.00851 | 0.00496 | 0.01460 |  | 0.01286 | 0.00805 | 0.02056 |
| 2010 | 5 | 70-79 | 0.00906 | 0.00526 | 0.01560 |  | 0.01436 | 0.00896 | 0.02304 |
| 2011 | 5 | 70-79 | 0.00982 | 0.00590 | 0.01633 |  | 0.01655 | 0.01074 | 0.02550 |
| 2012 | 5 | 70-79 | 0.01085 | 0.00697 | 0.01689 |  | 0.01947 | 0.01342 | 0.02824 |
| 2013 | 5 | 70-79 | 0.01217 | 0.00819 | 0.01808 |  | 0.02308 | 0.01664 | 0.03200 |
| 2014 | 5 | 70-79 | 0.01378 | 0.00918 | 0.02068 |  | 0.02720 | 0.01977 | 0.03743 |
| 2015 | 5 | 70-79 | 0.01566 | 0.01006 | 0.02438 |  | 0.03146 | 0.02258 | 0.04384 |
| 2016 | 5 | 70-79 | 0.01778 | 0.01141 | 0.02769 |  | 0.03524 | 0.02550 | 0.04871 |
| 2017 | 5 | 70-79 | 0.02006 | 0.01368 | 0.02939 |  | 0.03793 | 0.02874 | 0.05006 |
| 2018 | 5 | 70-79 | 0.02248 | 0.01626 | 0.03107 |  | 0.03982 | 0.03139 | 0.05051 |
| 2019 | 5 | 70-79 | 0.02503 | 0.01794 | 0.03492 |  | 0.04161 | 0.03249 | 0.05330 |
| 2020 | 5 | 70-79 | 0.02770 | 0.01925 | 0.03986 |  | 0.04419 | 0.03373 | 0.05790 |
| 2021 | 5 | 70-79 | 0.03049 | 0.02169 | 0.04287 |  | 0.04843 | 0.03763 | 0.06233 |
| 2022 | 5 | 70-79 | 0.03341 | 0.02494 | 0.04476 |  | 0.05455 | 0.04407 | 0.06752 |
| 2023 | 5 | 70-79 | 0.03651 | 0.02623 | 0.05082 |  | 0.06258 | 0.04957 | 0.07899 |
| 2024 | 5 | 70-79 | 0.03983 | 0.02434 | 0.06518 |  | 0.07244 | 0.05133 | 0.10222 |
| 2003 | 3 | 70-79 | 0.00646 | 0.00218 | 0.01914 |  | 0.01003 | 0.00303 | 0.03325 |
| 2004 | 3 | 70-79 | 0.00695 | 0.00287 | 0.01686 |  | 0.01061 | 0.00414 | 0.02716 |
| 2005 | 3 | 70-79 | 0.00749 | 0.00368 | 0.01523 |  | 0.01126 | 0.00553 | 0.02293 |
| 2006 | 3 | 70-79 | 0.00807 | 0.00454 | 0.01434 |  | 0.01205 | 0.00705 | 0.02059 |
| 2007 | 3 | 70-79 | 0.00871 | 0.00533 | 0.01424 |  | 0.01305 | 0.00839 | 0.02028 |
| 2008 | 3 | 70-79 | 0.00943 | 0.00597 | 0.01490 |  | 0.01436 | 0.00939 | 0.02197 |
| 2009 | 3 | 70-79 | 0.01023 | 0.00652 | 0.01604 |  | 0.01612 | 0.01037 | 0.02508 |
| 2010 | 3 | 70-79 | 0.01113 | 0.00717 | 0.01727 |  | 0.01855 | 0.01193 | 0.02883 |
| 2011 | 3 | 70-79 | 0.01216 | 0.00810 | 0.01825 |  | 0.02186 | 0.01462 | 0.03268 |
| 2012 | 3 | 70-79 | 0.01334 | 0.00934 | 0.01905 |  | 0.02600 | 0.01843 | 0.03668 |
| 2013 | 3 | 70-79 | 0.01470 | 0.01056 | 0.02045 |  | 0.03062 | 0.02261 | 0.04146 |
| 2014 | 3 | 70-79 | 0.01629 | 0.01148 | 0.02311 |  | 0.03502 | 0.02591 | 0.04732 |
| 2015 | 3 | 70-79 | 0.01815 | 0.01236 | 0.02665 |  | 0.03817 | 0.02777 | 0.05246 |
| 2016 | 3 | 70-79 | 0.02037 | 0.01387 | 0.02991 |  | 0.03890 | 0.02843 | 0.05323 |
| 2017 | 3 | 70-79 | 0.02301 | 0.01647 | 0.03214 |  | 0.03679 | 0.02796 | 0.04841 |
| 2018 | 3 | 70-79 | 0.02614 | 0.01947 | 0.03508 |  | 0.03356 | 0.02612 | 0.04311 |
| 2019 | 3 | 70-79 | 0.02980 | 0.02180 | 0.04073 |  | 0.03104 | 0.02356 | 0.04089 |
| 2020 | 3 | 70-79 | 0.03404 | 0.02420 | 0.04788 |  | 0.03061 | 0.02260 | 0.04146 |
| 2021 | 3 | 70-79 | 0.03891 | 0.02833 | 0.05343 |  | 0.03340 | 0.02518 | 0.04431 |
| 2022 | 3 | 70-79 | 0.04448 | 0.03392 | 0.05833 |  | 0.03971 | 0.03136 | 0.05029 |
| 2023 | 3 | 70-79 | 0.05086 | 0.03747 | 0.06902 |  | 0.04999 | 0.03900 | 0.06409 |
| 2024 | 3 | 70-79 | 0.05815 | 0.03694 | 0.09154 |  | 0.06476 | 0.04509 | 0.09302 |
| 2003 | 2 | 70-79 | 0.00651 | 0.00216 | 0.01960 |  | 0.00520 | 0.00181 | 0.01491 |
| 2004 | 2 | 70-79 | 0.00794 | 0.00333 | 0.01893 |  | 0.00591 | 0.00258 | 0.01356 |
| 2005 | 2 | 70-79 | 0.00964 | 0.00499 | 0.01862 |  | 0.00673 | 0.00353 | 0.01282 |
| 2006 | 2 | 70-79 | 0.01156 | 0.00705 | 0.01894 |  | 0.00765 | 0.00453 | 0.01292 |
| 2007 | 2 | 70-79 | 0.01362 | 0.00913 | 0.02031 |  | 0.00871 | 0.00538 | 0.01410 |
| 2008 | 2 | 70-79 | 0.01567 | 0.01070 | 0.02296 |  | 0.00991 | 0.00605 | 0.01625 |
| 2009 | 2 | 70-79 | 0.01752 | 0.01171 | 0.02622 |  | 0.01129 | 0.00675 | 0.01887 |
| 2010 | 2 | 70-79 | 0.01891 | 0.01245 | 0.02871 |  | 0.01287 | 0.00777 | 0.02130 |
| 2011 | 2 | 70-79 | 0.01959 | 0.01313 | 0.02922 |  | 0.01467 | 0.00933 | 0.02307 |
| 2012 | 2 | 70-79 | 0.01948 | 0.01369 | 0.02773 |  | 0.01665 | 0.01132 | 0.02448 |
| 2013 | 2 | 70-79 | 0.01903 | 0.01381 | 0.02622 |  | 0.01872 | 0.01332 | 0.02632 |
| 2014 | 2 | 70-79 | 0.01879 | 0.01345 | 0.02625 |  | 0.02077 | 0.01479 | 0.02915 |
| 2015 | 2 | 70-79 | 0.01930 | 0.01334 | 0.02792 |  | 0.02260 | 0.01581 | 0.03231 |
| 2016 | 2 | 70-79 | 0.02123 | 0.01462 | 0.03083 |  | 0.02401 | 0.01689 | 0.03412 |
| 2017 | 2 | 70-79 | 0.02543 | 0.01837 | 0.03518 |  | 0.02490 | 0.01834 | 0.03380 |
| 2018 | 2 | 70-79 | 0.03208 | 0.02425 | 0.04244 |  | 0.02565 | 0.01956 | 0.03363 |
| 2019 | 2 | 70-79 | 0.04075 | 0.03049 | 0.05447 |  | 0.02684 | 0.02013 | 0.03579 |
| 2020 | 2 | 70-79 | 0.04984 | 0.03636 | 0.06831 |  | 0.02917 | 0.02133 | 0.03990 |
| 2021 | 2 | 70-79 | 0.05670 | 0.04227 | 0.07606 |  | 0.03347 | 0.02503 | 0.04475 |
| 2022 | 2 | 70-79 | 0.06060 | 0.04697 | 0.07817 |  | 0.04017 | 0.03159 | 0.05109 |
| 2023 | 2 | 70-79 | 0.06212 | 0.04621 | 0.08352 |  | 0.04970 | 0.03870 | 0.06383 |
| 2024 | 2 | 70-79 | 0.06237 | 0.04007 | 0.09709 |  | 0.06242 | 0.04325 | 0.09008 |
| 2003 | 4 | 70-79 | 0.00764 | 0.00223 | 0.02623 |  | 0.00405 | 0.00108 | 0.01515 |
| 2004 | 4 | 70-79 | 0.00753 | 0.00284 | 0.01997 |  | 0.00443 | 0.00155 | 0.01269 |
| 2005 | 4 | 70-79 | 0.00746 | 0.00352 | 0.01579 |  | 0.00487 | 0.00210 | 0.01128 |
| 2006 | 4 | 70-79 | 0.00746 | 0.00417 | 0.01335 |  | 0.00537 | 0.00263 | 0.01100 |
| 2007 | 4 | 70-79 | 0.00756 | 0.00459 | 0.01244 |  | 0.00599 | 0.00302 | 0.01187 |
| 2008 | 4 | 70-79 | 0.00781 | 0.00477 | 0.01278 |  | 0.00675 | 0.00333 | 0.01369 |
| 2009 | 4 | 70-79 | 0.00825 | 0.00491 | 0.01387 |  | 0.00772 | 0.00372 | 0.01599 |
| 2010 | 4 | 70-79 | 0.00897 | 0.00527 | 0.01527 |  | 0.00898 | 0.00443 | 0.01820 |
| 2011 | 4 | 70-79 | 0.01008 | 0.00609 | 0.01668 |  | 0.01064 | 0.00569 | 0.01989 |
| 2012 | 4 | 70-79 | 0.01171 | 0.00757 | 0.01812 |  | 0.01269 | 0.00758 | 0.02127 |
| 2013 | 4 | 70-79 | 0.01390 | 0.00952 | 0.02029 |  | 0.01507 | 0.00983 | 0.02310 |
| 2014 | 4 | 70-79 | 0.01656 | 0.01140 | 0.02407 |  | 0.01756 | 0.01178 | 0.02617 |
| 2015 | 4 | 70-79 | 0.01951 | 0.01305 | 0.02917 |  | 0.01983 | 0.01313 | 0.02995 |
| 2016 | 4 | 70-79 | 0.02234 | 0.01492 | 0.03346 |  | 0.02144 | 0.01429 | 0.03217 |
| 2017 | 4 | 70-79 | 0.02460 | 0.01733 | 0.03492 |  | 0.02203 | 0.01551 | 0.03129 |
| 2018 | 4 | 70-79 | 0.02631 | 0.01941 | 0.03567 |  | 0.02195 | 0.01613 | 0.02986 |
| 2019 | 4 | 70-79 | 0.02776 | 0.02009 | 0.03836 |  | 0.02176 | 0.01563 | 0.03029 |
| 2020 | 4 | 70-79 | 0.02935 | 0.02054 | 0.04194 |  | 0.02202 | 0.01526 | 0.03179 |
| 2021 | 4 | 70-79 | 0.03148 | 0.02253 | 0.04398 |  | 0.02322 | 0.01642 | 0.03284 |
| 2022 | 4 | 70-79 | 0.03421 | 0.02564 | 0.04564 |  | 0.02536 | 0.01885 | 0.03413 |
| 2023 | 4 | 70-79 | 0.03750 | 0.02701 | 0.05206 |  | 0.02836 | 0.02057 | 0.03909 |
| 2024 | 4 | 70-79 | 0.04129 | 0.02535 | 0.06724 |  | 0.03208 | 0.02015 | 0.05108 |
| 2003 | 1 | 80-89 | 0.00693 | 0.00154 | 0.03115 |  | 0.02480 | 0.00834 | 0.07377 |
| 2004 | 1 | 80-89 | 0.00843 | 0.00249 | 0.02859 |  | 0.02492 | 0.01054 | 0.05890 |
| 2005 | 1 | 80-89 | 0.01027 | 0.00395 | 0.02671 |  | 0.02522 | 0.01312 | 0.04849 |
| 2006 | 1 | 80-89 | 0.01250 | 0.00606 | 0.02577 |  | 0.02592 | 0.01587 | 0.04231 |
| 2007 | 1 | 80-89 | 0.01521 | 0.00886 | 0.02612 |  | 0.02723 | 0.01840 | 0.04031 |
| 2008 | 1 | 80-89 | 0.01853 | 0.01211 | 0.02833 |  | 0.02949 | 0.02048 | 0.04246 |
| 2009 | 1 | 80-89 | 0.02257 | 0.01546 | 0.03294 |  | 0.03315 | 0.02283 | 0.04812 |
| 2010 | 1 | 80-89 | 0.02750 | 0.01905 | 0.03969 |  | 0.03897 | 0.02688 | 0.05650 |
| 2011 | 1 | 80-89 | 0.03352 | 0.02368 | 0.04746 |  | 0.04806 | 0.03429 | 0.06736 |
| 2012 | 1 | 80-89 | 0.04083 | 0.03010 | 0.05539 |  | 0.06112 | 0.04604 | 0.08114 |
| 2013 | 1 | 80-89 | 0.04935 | 0.03777 | 0.06448 |  | 0.07845 | 0.06191 | 0.09941 |
| 2014 | 1 | 80-89 | 0.05870 | 0.04488 | 0.07677 |  | 0.09944 | 0.07960 | 0.12423 |
| 2015 | 1 | 80-89 | 0.06814 | 0.05080 | 0.09142 |  | 0.12183 | 0.09661 | 0.15364 |
| 2016 | 1 | 80-89 | 0.07658 | 0.05682 | 0.10322 |  | 0.14118 | 0.11211 | 0.17778 |
| 2017 | 1 | 80-89 | 0.08305 | 0.06379 | 0.10814 |  | 0.15277 | 0.12481 | 0.18699 |
| 2018 | 1 | 80-89 | 0.08843 | 0.06956 | 0.11242 |  | 0.15785 | 0.13150 | 0.18947 |
| 2019 | 1 | 80-89 | 0.09453 | 0.07261 | 0.12306 |  | 0.16066 | 0.13161 | 0.19612 |
| 2020 | 1 | 80-89 | 0.10374 | 0.07755 | 0.13878 |  | 0.16618 | 0.13339 | 0.20702 |
| 2021 | 1 | 80-89 | 0.11884 | 0.09057 | 0.15594 |  | 0.17891 | 0.14571 | 0.21969 |
| 2022 | 1 | 80-89 | 0.14120 | 0.11150 | 0.17882 |  | 0.19957 | 0.16707 | 0.23840 |
| 2023 | 1 | 80-89 | 0.17190 | 0.13086 | 0.22581 |  | 0.22794 | 0.18609 | 0.27921 |
| 2024 | 1 | 80-89 | 0.21184 | 0.14136 | 0.31746 |  | 0.26343 | 0.19518 | 0.35554 |
| 2003 | 5 | 80-89 | 0.01154 | 0.00451 | 0.02954 |  | 0.00858 | 0.00070 | 0.10509 |
| 2004 | 5 | 80-89 | 0.01350 | 0.00635 | 0.02872 |  | 0.01102 | 0.00147 | 0.08256 |
| 2005 | 5 | 80-89 | 0.01581 | 0.00879 | 0.02846 |  | 0.01410 | 0.00301 | 0.06614 |
| 2006 | 5 | 80-89 | 0.01856 | 0.01181 | 0.02918 |  | 0.01794 | 0.00585 | 0.05499 |
| 2007 | 5 | 80-89 | 0.02185 | 0.01517 | 0.03148 |  | 0.02260 | 0.01053 | 0.04853 |
| 2008 | 5 | 80-89 | 0.02583 | 0.01855 | 0.03595 |  | 0.02813 | 0.01683 | 0.04702 |
| 2009 | 5 | 80-89 | 0.03068 | 0.02206 | 0.04266 |  | 0.03447 | 0.02304 | 0.05159 |
| 2010 | 5 | 80-89 | 0.03665 | 0.02634 | 0.05100 |  | 0.04146 | 0.02813 | 0.06112 |
| 2011 | 5 | 80-89 | 0.04409 | 0.03232 | 0.06017 |  | 0.04884 | 0.03373 | 0.07073 |
| 2012 | 5 | 80-89 | 0.05337 | 0.04071 | 0.06998 |  | 0.05644 | 0.04083 | 0.07802 |
| 2013 | 5 | 80-89 | 0.06449 | 0.05060 | 0.08219 |  | 0.06413 | 0.04868 | 0.08450 |
| 2014 | 5 | 80-89 | 0.07702 | 0.05993 | 0.09897 |  | 0.07185 | 0.05524 | 0.09345 |
| 2015 | 5 | 80-89 | 0.09004 | 0.06834 | 0.11865 |  | 0.07956 | 0.06003 | 0.10544 |
| 2016 | 5 | 80-89 | 0.10206 | 0.07728 | 0.13478 |  | 0.08729 | 0.06550 | 0.11632 |
| 2017 | 5 | 80-89 | 0.11155 | 0.08728 | 0.14257 |  | 0.09521 | 0.07403 | 0.12245 |
| 2018 | 5 | 80-89 | 0.11905 | 0.09492 | 0.14933 |  | 0.10392 | 0.08388 | 0.12875 |
| 2019 | 5 | 80-89 | 0.12618 | 0.09804 | 0.16240 |  | 0.11435 | 0.09176 | 0.14249 |
| 2020 | 5 | 80-89 | 0.13507 | 0.10226 | 0.17841 |  | 0.12776 | 0.10035 | 0.16265 |
| 2021 | 5 | 80-89 | 0.14795 | 0.11412 | 0.19180 |  | 0.14570 | 0.11616 | 0.18276 |
| 2022 | 5 | 80-89 | 0.16534 | 0.13152 | 0.20785 |  | 0.16902 | 0.13947 | 0.20484 |
| 2023 | 5 | 80-89 | 0.18728 | 0.14291 | 0.24542 |  | 0.19830 | 0.16057 | 0.24490 |
| 2024 | 5 | 80-89 | 0.21356 | 0.14302 | 0.31887 |  | 0.23398 | 0.17105 | 0.32007 |
| 2003 | 3 | 80-89 | 0.02295 | 0.00847 | 0.06222 |  | 0.01517 | 0.00469 | 0.04910 |
| 2004 | 3 | 80-89 | 0.02423 | 0.01081 | 0.05432 |  | 0.01829 | 0.00713 | 0.04692 |
| 2005 | 3 | 80-89 | 0.02563 | 0.01357 | 0.04842 |  | 0.02204 | 0.01064 | 0.04566 |
| 2006 | 3 | 80-89 | 0.02723 | 0.01658 | 0.04474 |  | 0.02654 | 0.01534 | 0.04591 |
| 2007 | 3 | 80-89 | 0.02911 | 0.01946 | 0.04353 |  | 0.03192 | 0.02100 | 0.04852 |
| 2008 | 3 | 80-89 | 0.03137 | 0.02191 | 0.04492 |  | 0.03834 | 0.02699 | 0.05448 |
| 2009 | 3 | 80-89 | 0.03416 | 0.02410 | 0.04843 |  | 0.04598 | 0.03305 | 0.06398 |
| 2010 | 3 | 80-89 | 0.03767 | 0.02673 | 0.05308 |  | 0.05503 | 0.04005 | 0.07562 |
| 2011 | 3 | 80-89 | 0.04213 | 0.03061 | 0.05799 |  | 0.06563 | 0.04927 | 0.08740 |
| 2012 | 3 | 80-89 | 0.04783 | 0.03623 | 0.06315 |  | 0.07746 | 0.06063 | 0.09898 |
| 2013 | 3 | 80-89 | 0.05477 | 0.04261 | 0.07039 |  | 0.08980 | 0.07216 | 0.11176 |
| 2014 | 3 | 80-89 | 0.06273 | 0.04825 | 0.08155 |  | 0.10146 | 0.08119 | 0.12678 |
| 2015 | 3 | 80-89 | 0.07128 | 0.05336 | 0.09523 |  | 0.11083 | 0.08719 | 0.14087 |
| 2016 | 3 | 80-89 | 0.07973 | 0.05956 | 0.10675 |  | 0.11614 | 0.09143 | 0.14754 |
| 2017 | 3 | 80-89 | 0.08733 | 0.06754 | 0.11293 |  | 0.11673 | 0.09445 | 0.14425 |
| 2018 | 3 | 80-89 | 0.09429 | 0.07444 | 0.11944 |  | 0.11586 | 0.09531 | 0.14085 |
| 2019 | 3 | 80-89 | 0.10133 | 0.07795 | 0.13173 |  | 0.11784 | 0.09511 | 0.14600 |
| 2020 | 3 | 80-89 | 0.10945 | 0.08198 | 0.14612 |  | 0.12743 | 0.10081 | 0.16107 |
| 2021 | 3 | 80-89 | 0.11969 | 0.09141 | 0.15671 |  | 0.15045 | 0.12109 | 0.18694 |
| 2022 | 3 | 80-89 | 0.13233 | 0.10438 | 0.16777 |  | 0.19120 | 0.15940 | 0.22935 |
| 2023 | 3 | 80-89 | 0.14739 | 0.11139 | 0.19502 |  | 0.25521 | 0.21009 | 0.31001 |
| 2024 | 3 | 80-89 | 0.16476 | 0.10875 | 0.24960 |  | 0.34910 | 0.26252 | 0.46423 |
| 2003 | 2 | 80-89 | 0.00442 | 0.00119 | 0.01644 |  | 0.01974 | 0.00453 | 0.08599 |
| 2004 | 2 | 80-89 | 0.00710 | 0.00246 | 0.02048 |  | 0.01873 | 0.00581 | 0.06036 |
| 2005 | 2 | 80-89 | 0.01128 | 0.00496 | 0.02567 |  | 0.01794 | 0.00730 | 0.04408 |
| 2006 | 2 | 80-89 | 0.01760 | 0.00953 | 0.03250 |  | 0.01751 | 0.00885 | 0.03464 |
| 2007 | 2 | 80-89 | 0.02665 | 0.01694 | 0.04192 |  | 0.01758 | 0.01017 | 0.03038 |
| 2008 | 2 | 80-89 | 0.03881 | 0.02711 | 0.05555 |  | 0.01832 | 0.01111 | 0.03021 |
| 2009 | 2 | 80-89 | 0.05383 | 0.03870 | 0.07488 |  | 0.02001 | 0.01212 | 0.03306 |
| 2010 | 2 | 80-89 | 0.07041 | 0.05062 | 0.09794 |  | 0.02313 | 0.01409 | 0.03796 |
| 2011 | 2 | 80-89 | 0.08602 | 0.06261 | 0.11818 |  | 0.02837 | 0.01810 | 0.04447 |
| 2012 | 2 | 80-89 | 0.09772 | 0.07388 | 0.12925 |  | 0.03622 | 0.02486 | 0.05279 |
| 2013 | 2 | 80-89 | 0.10510 | 0.08235 | 0.13414 |  | 0.04693 | 0.03442 | 0.06399 |
| 2014 | 2 | 80-89 | 0.10955 | 0.08596 | 0.13962 |  | 0.06013 | 0.04533 | 0.07976 |
| 2015 | 2 | 80-89 | 0.11327 | 0.08694 | 0.14758 |  | 0.07428 | 0.05566 | 0.09913 |
| 2016 | 2 | 80-89 | 0.11893 | 0.09087 | 0.15566 |  | 0.08623 | 0.06481 | 0.11472 |
| 2017 | 2 | 80-89 | 0.12904 | 0.10161 | 0.16387 |  | 0.09270 | 0.07228 | 0.11888 |
| 2018 | 2 | 80-89 | 0.14381 | 0.11548 | 0.17909 |  | 0.09497 | 0.07628 | 0.11824 |
| 2019 | 2 | 80-89 | 0.16268 | 0.12767 | 0.20729 |  | 0.09646 | 0.07627 | 0.12199 |
| 2020 | 2 | 80-89 | 0.18459 | 0.14134 | 0.24108 |  | 0.10106 | 0.07799 | 0.13095 |
| 2021 | 2 | 80-89 | 0.20809 | 0.16220 | 0.26697 |  | 0.11252 | 0.08828 | 0.14342 |
| 2022 | 2 | 80-89 | 0.23304 | 0.18706 | 0.29032 |  | 0.13209 | 0.10756 | 0.16222 |
| 2023 | 2 | 80-89 | 0.25982 | 0.20024 | 0.33714 |  | 0.16063 | 0.12841 | 0.20092 |
| 2024 | 2 | 80-89 | 0.28905 | 0.19629 | 0.42564 |  | 0.19880 | 0.14295 | 0.27647 |
| 2003 | 4 | 80-89 | 0.00385 | 0.00077 | 0.01915 |  | 0.01007 | 0.00125 | 0.08077 |
| 2004 | 4 | 80-89 | 0.00540 | 0.00147 | 0.01986 |  | 0.01067 | 0.00198 | 0.05757 |
| 2005 | 4 | 80-89 | 0.00754 | 0.00272 | 0.02090 |  | 0.01141 | 0.00307 | 0.04239 |
| 2006 | 4 | 80-89 | 0.01050 | 0.00488 | 0.02259 |  | 0.01242 | 0.00465 | 0.03318 |
| 2007 | 4 | 80-89 | 0.01451 | 0.00826 | 0.02547 |  | 0.01387 | 0.00674 | 0.02853 |
| 2008 | 4 | 80-89 | 0.01986 | 0.01292 | 0.03053 |  | 0.01604 | 0.00923 | 0.02788 |
| 2009 | 4 | 80-89 | 0.02687 | 0.01848 | 0.03905 |  | 0.01938 | 0.01202 | 0.03125 |
| 2010 | 4 | 80-89 | 0.03585 | 0.02496 | 0.05148 |  | 0.02468 | 0.01577 | 0.03864 |
| 2011 | 4 | 80-89 | 0.04705 | 0.03333 | 0.06643 |  | 0.03318 | 0.02211 | 0.04980 |
| 2012 | 4 | 80-89 | 0.06058 | 0.04486 | 0.08181 |  | 0.04595 | 0.03261 | 0.06474 |
| 2013 | 4 | 80-89 | 0.07611 | 0.05899 | 0.09821 |  | 0.06351 | 0.04781 | 0.08437 |
| 2014 | 4 | 80-89 | 0.09277 | 0.07255 | 0.11863 |  | 0.08488 | 0.06547 | 0.11004 |
| 2015 | 4 | 80-89 | 0.10907 | 0.08347 | 0.14252 |  | 0.10630 | 0.08144 | 0.13875 |
| 2016 | 4 | 80-89 | 0.12297 | 0.09347 | 0.16177 |  | 0.12087 | 0.09280 | 0.15744 |
| 2017 | 4 | 80-89 | 0.13280 | 0.10405 | 0.16949 |  | 0.12268 | 0.09739 | 0.15453 |
| 2018 | 4 | 80-89 | 0.13984 | 0.11192 | 0.17472 |  | 0.11574 | 0.09400 | 0.14251 |
| 2019 | 4 | 80-89 | 0.14684 | 0.11482 | 0.18779 |  | 0.10726 | 0.08519 | 0.13504 |
| 2020 | 4 | 80-89 | 0.15725 | 0.11976 | 0.20649 |  | 0.10316 | 0.07977 | 0.13341 |
| 2021 | 4 | 80-89 | 0.17469 | 0.13538 | 0.22541 |  | 0.10740 | 0.08434 | 0.13678 |
| 2022 | 4 | 80-89 | 0.20028 | 0.16011 | 0.25053 |  | 0.11983 | 0.09727 | 0.14764 |
| 2023 | 4 | 80-89 | 0.23451 | 0.18032 | 0.30500 |  | 0.14002 | 0.11076 | 0.17700 |
| 2024 | 4 | 80-89 | 0.27751 | 0.18785 | 0.40996 |  | 0.16741 | 0.11856 | 0.23640 |

### Model estimates

| Model | Term | IRR_95_CI | Estimate_95_CI | p_value_formatted |
| --- | --- | --- | --- | --- |
| Female | AGEGROUP70-79 | 1.722 (0.286-17.049) | 0.543 (-1.250-2.836) | 0.583 |
| Female | AGEGROUP80-89 | 1.846 (0.196-22.295) | 0.613 (-1.630-3.104) | 0.596 |
| Female | cluster2 | 1.019 (0.024-23.071) | 0.019 (-3.710-3.139) | 0.991 |
| Female | cluster2#AGEGROUP70-79 | 0.988 (0.032-51.878) | -0.012 (-3.445-3.949) | 0.995 |
| Female | cluster2#AGEGROUP80-89 | 0.626 (0.016-40.127) | -0.469 (-4.134-3.692) | 0.807 |
| Female | cluster3 | 0.902 (0.054-13.841) | -0.103 (-2.925-2.628) | 0.938 |
| Female | cluster3#AGEGROUP70-79 | 1.108 (0.051-25.574) | 0.102 (-2.977-3.242) | 0.946 |
| Female | cluster3#AGEGROUP80-89 | 3.672 (0.149-103.122) | 1.301 (-1.905-4.636) | 0.422 |
| Female | cluster4 | 2.834 (0.198-45.155) | 1.042 (-1.619-3.810) | 0.435 |
| Female | cluster4#AGEGROUP70-79 | 0.417 (0.018-8.919) | -0.875 (-4.029-2.188) | 0.573 |
| Female | cluster4#AGEGROUP80-89 | 0.196 (0.006-6.007) | -1.629 (-5.124-1.793) | 0.35 |
| Female | cluster5 | 1.732 (0.190-20.146) | 0.549 (-1.660-3.003) | 0.625 |
| Female | cluster5#AGEGROUP70-79 | 0.658 (0.039-9.108) | -0.419 (-3.237-2.209) | 0.756 |
| Female | cluster5#AGEGROUP80-89 | 0.961 (0.051-16.779) | -0.040 (-2.974-2.820) | 0.978 |
| Female | ns(YEAR, df = knots_number)1#AGEGROUP70-79 | 0.842 (0.080-6.278) | -0.172 (-2.530-1.837) | 0.873 |
| Female | ns(YEAR, df = knots_number)1#AGEGROUP80-89 | 4.243 (0.351-42.319) | 1.445 (-1.048-3.745) | 0.222 |
| Female | ns(YEAR, df = knots_number)1#cluster2 | 1.958 (0.089-72.746) | 0.672 (-2.417-4.287) | 0.677 |
| Female | ns(YEAR, df = knots_number)1#cluster2#AGEGROUP70-79 | 0.473 (0.010-14.643) | -0.748 (-4.625-2.684) | 0.675 |
| Female | ns(YEAR, df = knots_number)1#cluster2#AGEGROUP80-89 | 0.965 (0.017-34.998) | -0.036 (-4.056-3.555) | 0.985 |
| Female | ns(YEAR, df = knots_number)1#cluster3 | 1.011 (0.063-16.902) | 0.011 (-2.767-2.827) | 0.994 |
| Female | ns(YEAR, df = knots_number)1#cluster3#AGEGROUP70-79 | 1.205 (0.049-29.200) | 0.187 (-3.021-3.374) | 0.906 |
| Female | ns(YEAR, df = knots_number)1#cluster3#AGEGROUP80-89 | 0.294 (0.011-7.444) | -1.225 (-4.534-2.007) | 0.453 |
| Female | ns(YEAR, df = knots_number)1#cluster4 | 0.592 (0.039-8.341) | -0.524 (-3.246-2.121) | 0.693 |
| Female | ns(YEAR, df = knots_number)1#cluster4#AGEGROUP70-79 | 2.269 (0.104-52.753) | 0.819 (-2.263-3.966) | 0.6 |
| Female | ns(YEAR, df = knots_number)1#cluster4#AGEGROUP80-89 | 4.998 (0.178-147.832) | 1.609 (-1.725-4.996) | 0.342 |
| Female | ns(YEAR, df = knots_number)1#cluster5 | 1.316 (0.095-15.528) | 0.275 (-2.351-2.743) | 0.827 |
| Female | ns(YEAR, df = knots_number)1#cluster5#AGEGROUP70-79 | 0.741 (0.040-15.651) | -0.299 (-3.231-2.751) | 0.842 |
| Female | ns(YEAR, df = knots_number)1#cluster5#AGEGROUP80-89 | 0.607 (0.030-13.095) | -0.500 (-3.522-2.572) | 0.744 |
| Female | ns(YEAR, df = knots_number)2#AGEGROUP70-79 | 1.620 (0.268-7.817) | 0.482 (-1.316-2.056) | 0.564 |
| Female | ns(YEAR, df = knots_number)2#AGEGROUP80-89 | 3.309 (0.493-19.747) | 1.197 (-0.708-2.983) | 0.191 |
| Female | ns(YEAR, df = knots_number)2#cluster2 | 0.839 (0.080-12.595) | -0.175 (-2.522-2.533) | 0.887 |
| Female | ns(YEAR, df = knots_number)2#cluster2#AGEGROUP70-79 | 1.756 (0.092-24.964) | 0.563 (-2.385-3.217) | 0.684 |
| Female | ns(YEAR, df = knots_number)2#cluster2#AGEGROUP80-89 | 2.217 (0.104-35.529) | 0.796 (-2.263-3.570) | 0.582 |
| Female | ns(YEAR, df = knots_number)2#cluster3 | 0.746 (0.088-6.581) | -0.293 (-2.433-1.884) | 0.782 |
| Female | ns(YEAR, df = knots_number)2#cluster3#AGEGROUP70-79 | 1.403 (0.115-16.806) | 0.339 (-2.166-2.822) | 0.785 |
| Female | ns(YEAR, df = knots_number)2#cluster3#AGEGROUP80-89 | 0.622 (0.047-7.828) | -0.475 (-3.068-2.058) | 0.711 |
| Female | ns(YEAR, df = knots_number)2#cluster4 | 0.205 (0.022-1.797) | -1.587 (-3.820-0.586) | 0.152 |
| Female | ns(YEAR, df = knots_number)2#cluster4#AGEGROUP70-79 | 4.253 (0.336-56.577) | 1.448 (-1.091-4.036) | 0.266 |
| Female | ns(YEAR, df = knots_number)2#cluster4#AGEGROUP80-89 | 10.226 (0.679-160.970) | 2.325 (-0.388-5.081) | 0.094 |
| Female | ns(YEAR, df = knots_number)2#cluster5 | 0.362 (0.047-2.511) | -1.016 (-3.065-0.921) | 0.306 |
| Female | ns(YEAR, df = knots_number)2#cluster5#AGEGROUP70-79 | 2.388 (0.234-26.414) | 0.870 (-1.451-3.274) | 0.466 |
| Female | ns(YEAR, df = knots_number)2#cluster5#AGEGROUP80-89 | 2.478 (0.225-28.267) | 0.907 (-1.491-3.342) | 0.456 |
| Female | ns(YEAR, df = knots_number)3#AGEGROUP70-79 | 2.789 (0.018-190.815) | 1.026 (-4.040-5.251) | 0.653 |
| Female | ns(YEAR, df = knots_number)3#AGEGROUP80-89 | 18.869 (0.083-2764.041) | 2.938 (-2.488-7.924) | 0.253 |
| Female | ns(YEAR, df = knots_number)3#cluster2 | 1.570 (0.002-4287.280) | 0.451 (-6.358-8.363) | 0.899 |
| Female | ns(YEAR, df = knots_number)3#cluster2#AGEGROUP70-79 | 1.828 (0.000-3836.750) | 0.603 (-7.964-8.252) | 0.88 |
| Female | ns(YEAR, df = knots_number)3#cluster2#AGEGROUP80-89 | 8.363 (0.001-25600.513) | 2.124 (-6.787-10.150) | 0.613 |
| Female | ns(YEAR, df = knots_number)3#cluster3 | 2.498 (0.005-1505.710) | 0.915 (-5.242-7.317) | 0.765 |
| Female | ns(YEAR, df = knots_number)3#cluster3#AGEGROUP70-79 | 0.422 (0.000-472.911) | -0.862 (-8.052-6.159) | 0.807 |
| Female | ns(YEAR, df = knots_number)3#cluster3#AGEGROUP80-89 | 0.046 (0.000-62.135) | -3.088 (-10.601-4.129) | 0.402 |
| Female | ns(YEAR, df = knots_number)3#cluster4 | 0.033 (0.000-16.504) | -3.418 (-9.903-2.804) | 0.281 |
| Female | ns(YEAR, df = knots_number)3#cluster4#AGEGROUP70-79 | 11.918 (0.008-20903.110) | 2.478 (-4.782-9.948) | 0.504 |
| Female | ns(YEAR, df = knots_number)3#cluster4#AGEGROUP80-89 | 177.490 (0.072-527199.794) | 5.179 (-2.634-13.175) | 0.197 |
| Female | ns(YEAR, df = knots_number)3#cluster5 | 0.072 (0.000-12.029) | -2.635 (-8.178-2.487) | 0.314 |
| Female | ns(YEAR, df = knots_number)3#cluster5#AGEGROUP70-79 | 6.378 (0.013-4302.699) | 1.853 (-4.350-8.367) | 0.562 |
| Female | ns(YEAR, df = knots_number)3#cluster5#AGEGROUP80-89 | 7.403 (0.011-5614.036) | 2.002 (-4.506-8.633) | 0.544 |
| Female | ns(YEAR, df = knots_number)4#AGEGROUP70-79 | 1.127 (0.304-3.921) | 0.120 (-1.190-1.366) | 0.855 |
| Female | ns(YEAR, df = knots_number)4#AGEGROUP80-89 | 2.220 (0.607-7.659) | 0.798 (-0.499-2.036) | 0.219 |
| Female | ns(YEAR, df = knots_number)4#cluster2 | 1.388 (0.306-6.773) | 0.328 (-1.183-1.913) | 0.675 |
| Female | ns(YEAR, df = knots_number)4#cluster2#AGEGROUP70-79 | 0.438 (0.071-2.565) | -0.825 (-2.647-0.942) | 0.369 |
| Female | ns(YEAR, df = knots_number)4#cluster2#AGEGROUP80-89 | 0.505 (0.083-2.910) | -0.684 (-2.493-1.068) | 0.45 |
| Female | ns(YEAR, df = knots_number)4#cluster3 | 0.534 (0.123-2.274) | -0.627 (-2.098-0.821) | 0.401 |
| Female | ns(YEAR, df = knots_number)4#cluster3#AGEGROUP70-79 | 1.776 (0.310-10.346) | 0.575 (-1.171-2.337) | 0.524 |
| Female | ns(YEAR, df = knots_number)4#cluster3#AGEGROUP80-89 | 0.778 (0.141-4.350) | -0.251 (-1.961-1.470) | 0.774 |
| Female | ns(YEAR, df = knots_number)4#cluster4 | 0.861 (0.187-3.932) | -0.150 (-1.679-1.369) | 0.848 |
| Female | ns(YEAR, df = knots_number)4#cluster4#AGEGROUP70-79 | 0.944 (0.153-5.881) | -0.058 (-1.880-1.772) | 0.951 |
| Female | ns(YEAR, df = knots_number)4#cluster4#AGEGROUP80-89 | 1.555 (0.260-9.367) | 0.441 (-1.346-2.237) | 0.631 |
| Female | ns(YEAR, df = knots_number)4#cluster5 | 0.917 (0.175-4.965) | -0.087 (-1.744-1.602) | 0.919 |
| Female | ns(YEAR, df = knots_number)4#cluster5#AGEGROUP70-79 | 0.806 (0.112-5.672) | -0.216 (-2.189-1.736) | 0.83 |
| Female | ns(YEAR, df = knots_number)4#cluster5#AGEGROUP80-89 | 0.773 (0.116-5.024) | -0.258 (-2.158-1.614) | 0.79 |
| Male | AGEGROUP70-79 | 0.961 (0.234-3.831) | -0.039 (-1.453-1.343) | 0.955 |
| Male | AGEGROUP80-89 | 2.573 (0.617-10.317) | 0.945 (-0.482-2.334) | 0.184 |
| Male | cluster2 | 0.872 (0.182-3.863) | -0.137 (-1.703-1.351) | 0.858 |
| Male | cluster2#AGEGROUP70-79 | 0.644 (0.076-5.546) | -0.441 (-2.572-1.713) | 0.685 |
| Male | cluster2#AGEGROUP80-89 | 0.912 (0.080-9.618) | -0.092 (-2.525-2.264) | 0.939 |
| Male | cluster3 | 0.671 (0.180-2.458) | -0.399 (-1.716-0.899) | 0.543 |
| Male | cluster3#AGEGROUP70-79 | 1.614 (0.200-12.706) | 0.479 (-1.608-2.542) | 0.649 |
| Male | cluster3#AGEGROUP80-89 | 0.912 (0.113-7.050) | -0.093 (-2.176-1.953) | 0.93 |
| Male | cluster4 | 0.433 (0.037-2.960) | -0.838 (-3.285-1.085) | 0.436 |
| Male | cluster4#AGEGROUP70-79 | 1.009 (0.071-18.403) | 0.009 (-2.643-2.913) | 0.995 |
| Male | cluster4#AGEGROUP80-89 | 0.938 (0.035-23.293) | -0.064 (-3.357-3.148) | 0.968 |
| Male | cluster5 | 0.836 (0.226-3.034) | -0.179 (-1.488-1.110) | 0.783 |
| Male | cluster5#AGEGROUP70-79 | 1.336 (0.187-9.657) | 0.290 (-1.677-2.268) | 0.773 |
| Male | cluster5#AGEGROUP80-89 | 0.414 (0.017-7.497) | -0.882 (-4.069-2.014) | 0.566 |
| Male | ns(YEAR, df = knots_number)1#AGEGROUP70-79 | 4.963 (1.131-22.448) | 1.602 (0.123-3.111) | 0.035 |
| Male | ns(YEAR, df = knots_number)1#AGEGROUP80-89 | 6.288 (1.450-28.362) | 1.839 (0.371-3.345) | 0.015 |
| Male | ns(YEAR, df = knots_number)1#cluster2 | 1.237 (0.247-6.454) | 0.213 (-1.397-1.865) | 0.797 |
| Male | ns(YEAR, df = knots_number)1#cluster2#AGEGROUP70-79 | 0.715 (0.077-6.559) | -0.336 (-2.567-1.881) | 0.766 |
| Male | ns(YEAR, df = knots_number)1#cluster2#AGEGROUP80-89 | 0.648 (0.061-7.297) | -0.434 (-2.789-1.987) | 0.72 |
| Male | ns(YEAR, df = knots_number)1#cluster3 | 0.875 (0.170-4.404) | -0.134 (-1.772-1.483) | 0.871 |
| Male | ns(YEAR, df = knots_number)1#cluster3#AGEGROUP70-79 | 1.118 (0.120-10.750) | 0.111 (-2.124-2.375) | 0.922 |
| Male | ns(YEAR, df = knots_number)1#cluster3#AGEGROUP80-89 | 1.458 (0.160-13.800) | 0.377 (-1.832-2.625) | 0.739 |
| Male | ns(YEAR, df = knots_number)1#cluster4 | 1.110 (0.116-13.661) | 0.104 (-2.158-2.615) | 0.93 |
| Male | ns(YEAR, df = knots_number)1#cluster4#AGEGROUP70-79 | 1.020 (0.051-18.386) | 0.020 (-2.983-2.912) | 0.989 |
| Male | ns(YEAR, df = knots_number)1#cluster4#AGEGROUP80-89 | 2.397 (0.096-67.578) | 0.874 (-2.341-4.213) | 0.594 |
| Male | ns(YEAR, df = knots_number)1#cluster5 | 1.851 (0.395-8.699) | 0.616 (-0.928-2.163) | 0.432 |
| Male | ns(YEAR, df = knots_number)1#cluster5#AGEGROUP70-79 | 0.365 (0.043-3.052) | -1.007 (-3.137-1.116) | 0.352 |
| Male | ns(YEAR, df = knots_number)1#cluster5#AGEGROUP80-89 | 0.739 (0.044-16.085) | -0.302 (-3.132-2.778) | 0.84 |
| Male | ns(YEAR, df = knots_number)2#AGEGROUP70-79 | 2.239 (0.704-7.273) | 0.806 (-0.351-1.984) | 0.174 |
| Male | ns(YEAR, df = knots_number)2#AGEGROUP80-89 | 2.932 (0.929-9.496) | 1.076 (-0.073-2.251) | 0.068 |
| Male | ns(YEAR, df = knots_number)2#cluster2 | 0.864 (0.217-3.516) | -0.146 (-1.530-1.257) | 0.836 |
| Male | ns(YEAR, df = knots_number)2#cluster2#AGEGROUP70-79 | 0.967 (0.154-6.030) | -0.033 (-1.869-1.797) | 0.972 |
| Male | ns(YEAR, df = knots_number)2#cluster2#AGEGROUP80-89 | 0.926 (0.136-6.548) | -0.077 (-1.992-1.879) | 0.938 |
| Male | ns(YEAR, df = knots_number)2#cluster3 | 0.317 (0.083-1.188) | -1.149 (-2.489-0.172) | 0.09 |
| Male | ns(YEAR, df = knots_number)2#cluster3#AGEGROUP70-79 | 1.427 (0.233-8.944) | 0.356 (-1.458-2.191) | 0.702 |
| Male | ns(YEAR, df = knots_number)2#cluster3#AGEGROUP80-89 | 2.443 (0.414-14.819) | 0.893 (-0.881-2.696) | 0.327 |
| Male | ns(YEAR, df = knots_number)2#cluster4 | 1.015 (0.194-6.570) | 0.014 (-1.641-1.882) | 0.987 |
| Male | ns(YEAR, df = knots_number)2#cluster4#AGEGROUP70-79 | 0.891 (0.092-7.769) | -0.116 (-2.381-2.050) | 0.917 |
| Male | ns(YEAR, df = knots_number)2#cluster4#AGEGROUP80-89 | 1.209 (0.108-14.491) | 0.190 (-2.225-2.674) | 0.877 |
| Male | ns(YEAR, df = knots_number)2#cluster5 | 0.866 (0.258-2.902) | -0.143 (-1.353-1.065) | 0.815 |
| Male | ns(YEAR, df = knots_number)2#cluster5#AGEGROUP70-79 | 0.985 (0.186-5.214) | -0.015 (-1.684-1.651) | 0.985 |
| Male | ns(YEAR, df = knots_number)2#cluster5#AGEGROUP80-89 | 1.718 (0.192-18.195) | 0.541 (-1.650-2.901) | 0.639 |
| Male | ns(YEAR, df = knots_number)3#AGEGROUP70-79 | 9.821 (0.343-294.566) | 2.285 (-1.070-5.686) | 0.183 |
| Male | ns(YEAR, df = knots_number)3#AGEGROUP80-89 | 8.174 (0.297-238.365) | 2.101 (-1.212-5.474) | 0.216 |
| Male | ns(YEAR, df = knots_number)3#cluster2 | 0.698 (0.016-34.215) | -0.360 (-4.142-3.533) | 0.852 |
| Male | ns(YEAR, df = knots_number)3#cluster2#AGEGROUP70-79 | 2.447 (0.013-460.155) | 0.895 (-4.367-6.132) | 0.737 |
| Male | ns(YEAR, df = knots_number)3#cluster2#AGEGROUP80-89 | 0.895 (0.003-277.173) | -0.111 (-5.726-5.625) | 0.969 |
| Male | ns(YEAR, df = knots_number)3#cluster3 | 2.437 (0.089-70.238) | 0.891 (-2.419-4.252) | 0.597 |
| Male | ns(YEAR, df = knots_number)3#cluster3#AGEGROUP70-79 | 0.238 (0.002-37.732) | -1.437 (-6.473-3.631) | 0.576 |
| Male | ns(YEAR, df = knots_number)3#cluster3#AGEGROUP80-89 | 2.000 (0.015-273.458) | 0.693 (-4.170-5.611) | 0.781 |
| Male | ns(YEAR, df = knots_number)3#cluster4 | 1.402 (0.008-500.739) | 0.338 (-4.793-6.216) | 0.902 |
| Male | ns(YEAR, df = knots_number)3#cluster4#AGEGROUP70-79 | 0.745 (0.001-567.451) | -0.294 (-7.306-6.341) | 0.932 |
| Male | ns(YEAR, df = knots_number)3#cluster4#AGEGROUP80-89 | 1.442 (0.001-2851.924) | 0.366 (-7.122-7.956) | 0.923 |
| Male | ns(YEAR, df = knots_number)3#cluster5 | 0.648 (0.022-19.654) | -0.434 (-3.822-2.978) | 0.801 |
| Male | ns(YEAR, df = knots_number)3#cluster5#AGEGROUP70-79 | 0.862 (0.006-118.803) | -0.148 (-5.085-4.777) | 0.953 |
| Male | ns(YEAR, df = knots_number)3#cluster5#AGEGROUP80-89 | 15.638 (0.021-18899.570) | 2.750 (-3.874-9.847) | 0.429 |
| Male | ns(YEAR, df = knots_number)4#AGEGROUP70-79 | 2.861 (1.115-7.289) | 1.051 (0.109-1.986) | 0.028 |
| Male | ns(YEAR, df = knots_number)4#AGEGROUP80-89 | 3.257 (1.289-8.171) | 1.181 (0.254-2.101) | 0.012 |
| Male | ns(YEAR, df = knots_number)4#cluster2 | 0.507 (0.156-1.614) | -0.678 (-1.857-0.479) | 0.255 |
| Male | ns(YEAR, df = knots_number)4#cluster2#AGEGROUP70-79 | 1.581 (0.374-6.796) | 0.458 (-0.984-1.916) | 0.536 |
| Male | ns(YEAR, df = knots_number)4#cluster2#AGEGROUP80-89 | 2.320 (0.553-9.962) | 0.842 (-0.592-2.299) | 0.254 |
| Male | ns(YEAR, df = knots_number)4#cluster3 | 0.575 (0.191-1.709) | -0.553 (-1.654-0.536) | 0.323 |
| Male | ns(YEAR, df = knots_number)4#cluster3#AGEGROUP70-79 | 0.990 (0.253-3.903) | -0.010 (-1.373-1.362) | 0.989 |
| Male | ns(YEAR, df = knots_number)4#cluster3#AGEGROUP80-89 | 1.885 (0.505-7.093) | 0.634 (-0.682-1.959) | 0.348 |
| Male | ns(YEAR, df = knots_number)4#cluster4 | 1.797 (0.451-7.949) | 0.586 (-0.796-2.073) | 0.421 |
| Male | ns(YEAR, df = knots_number)4#cluster4#AGEGROUP70-79 | 0.341 (0.056-1.977) | -1.076 (-2.886-0.682) | 0.236 |
| Male | ns(YEAR, df = knots_number)4#cluster4#AGEGROUP80-89 | 0.712 (0.124-3.932) | -0.339 (-2.087-1.369) | 0.699 |
| Male | ns(YEAR, df = knots_number)4#cluster5 | 2.409 (0.781-7.658) | 0.879 (-0.247-2.036) | 0.131 |
| Male | ns(YEAR, df = knots_number)4#cluster5#AGEGROUP70-79 | 0.307 (0.075-1.228) | -1.180 (-2.585-0.205) | 0.098 |
| Male | ns(YEAR, df = knots_number)4#cluster5#AGEGROUP80-89 | 0.419 (0.095-1.878) | -0.869 (-2.349-0.630) | 0.252 |
